# Increasing the Recycling of PVC Flooring Requires Phthalate Removal for Ensuring Consumers’ Safety: A Cross-Checked Substance Flow Analysis of Plasticizers for Switzerland

**DOI:** 10.1021/acs.est.4c04164

**Published:** 2024-10-07

**Authors:** Magdalena Klotz, Sarah Schmidt, Helene Wiesinger, David Laner, Zhanyun Wang, Stefanie Hellweg

**Affiliations:** † Chair of Ecological Systems Design, Institute of Environmental Engineering, ETH Zürich, 8093 Zürich, Switzerland; ‡ Center for Resource Management and Solid Waste Engineering, Institute of Water, Waste and Environmental Engineering, University of Kassel, 34125 Kassel, Germany; § Empa - Swiss Federal Laboratories for Materials Science and Technology, Technology and Society Laboratory, 9014 St. Gallen, Switzerland; ∥ National Centre of Competence in Research (NCCR) Catalysis, Institute of Environmental Engineering, ETH Zürich, 8093 Zürich, Switzerland

**Keywords:** plastics, circular economy, legacy additives, material flow analysis, PVC recycling

## Abstract

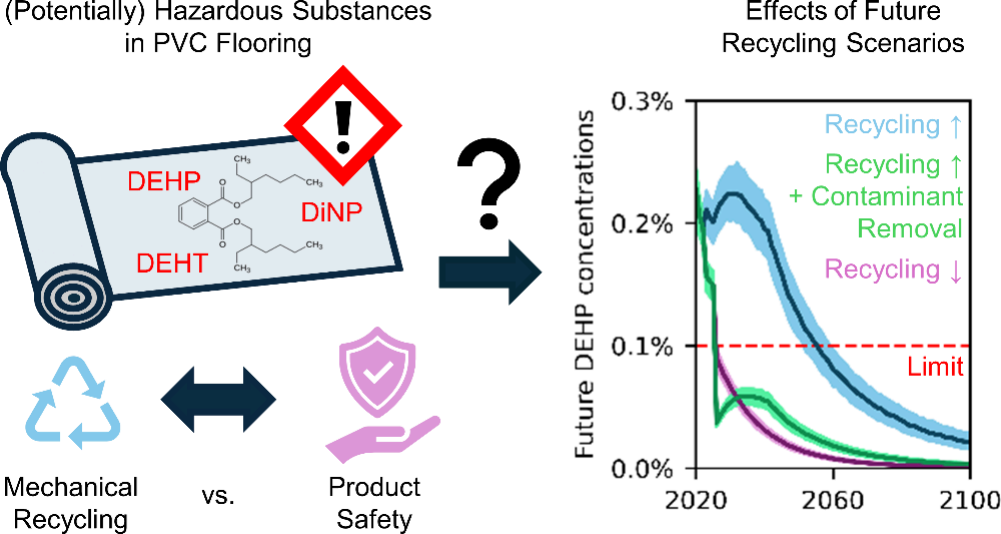

As our planet grapples with the severe repercussions of plastic pollution, mechanical recycling has been proposed as a potential remedy. However, increasing mechanical recycling may have unintended negative consequences. For example, recycling of PVC flooring containing hazardous plasticizers that were used in the past may lead to continued exposure. Here we propose measures to increase recycling while circumventing adverse health impacts caused by legacy additives. For this, we conduct a dynamic substance flow analysis for Switzerland and the time period from 1950 to 2100, focusing on three plasticizers: di(2-ethylhexyl) phthalate (DEHP), diisononyl phthalate (DiNP), and di(2-ethylhexyl) terephthalate (DEHT). We quantify the uncertainty of results, check their plausibility against measured concentrations in samples representative for the Swiss market, and compare them with modeled substance flows in Germany. Based on the cross-checked model, future average concentrations of DEHP in PVC flooring on the Swiss market are expected to be above the legal limit of 0.1 wt % for several decades if increased recycling rates are implemented without additional measures. Phasing out the potentially concerning DiNP, too, and preventing phthalates from entering recycling would lower their average market concentrations to values below 0.1 wt % and enable increasing recycling rates without compromising product safety. Analogous measures could help achieve this goal across other European countries and product groups.

## Abstract 2

Increased plastic recycling is strongly promoted by science and industry, but potentially related health risks are often not considered. This study highlights that legacy additives may exceed legal limits for several decades if recycling is increased without measures for contaminant removal.

## 1 Introduction

Poly(vinyl chloride) (PVC) flooring has been widely used in offices, residential spaces, hospitals, and schools due to different convenient characteristics.^1−4^ To achieve the performance required, a multitude of additives have been used in PVC, some of which were found to be hazardous.^5^ Flooring containing these substances is still in use and will continue to arise as waste in the future.^6^ While recycling this waste may reduce climate change impacts of PVC flooring,^7,8^ it can at the same time lead to prolonged consumer exposure to hazardous chemicals.^6,9,10^

Plasticizers are one mass-wise important group of additives used in flooring PVC.^5^ Up until now, predominantly *ortho*-phthalates (commonly simply referred to as phthalates) have been utilized for this purpose,^5^ some of which were shown to have harmful properties.^11^ PVC flooring has been identified as a major contributor to elevated concentrations of *ortho*-phthalates in indoor air and dust, and thus, is responsible for a large portion of total indoor exposure to these substances.^12−15^ Because of their hazardousness, some of the *ortho*-phthalates initially employed are no longer allowed for use without authorization^12,13^ or restricted in certain products such as toys and childcare articles^12,14^ in Europe and Switzerland. Alternative chemicals used as plasticizers include *tere*-phthalates, aliphatics, cyclohexanoates, benzoates, or trimellitates,^5,15^ for which the currently available data show less cause for concern.^16−18^ These substances are used in smaller amounts, but with increasing trends.^15,19^

A common method for investigating consumption, waste and recycling amounts of substances such as plasticizers within specific socio-economic systems over a certain time period is substance flow analysis.^20−24^ By this means, Tukker et al.^25^ have investigated the amounts of phthalates and other substances used in flooring and other PVC products in Sweden in 1994 and quantified their presence in the product stock and waste. Phthalate amounts corresponded to more than one-third of the PVC amount in floorings in use, waste, and stock. However, the study does not distinguish between different phthalates and refers to a past situation which has drastically changed by now.^5^ More recently, a dynamic substance flow analysis was conducted for estimating flows, stocks, and emissions of the *ortho*-phthalate di(2-ethylhexyl) phthalate (DEHP) in building materials and other product segments in Japan.^26^ Following an initial exponential increase, the total DEHP consumption in Japan leveled out after 1992, succeeded by an exponential decline after 2003. Building materials contained about one-fifth of the total DEHP stock. Almost one-third of industrial (including construction) waste was mechanically recycled in the recent past and about 12 wt% chemically. However, no flooring-specific data or data for other plasticizers than DEHP are available from the study. In another study, the amount of DEHP in flooring was investigated for the city of Stockholm in 2002,^27^ where the use of DEHP in flooring had already ceased by that time according to the major manufacturers and trade organizations.^27^

The considerable past consumption of DEHP and other potentially harmful plasticizers comes together with a general trend toward making our socio-economic material flows more circular,^28^ a situation that may lead to keeping hazardous substances in the product cycle. A means to achieve high recycling rates while avoiding exposure may be the removal of hazardous-substance-containing waste from mechanical recycling streams. This requires the identification of contaminated waste. Measurement options are available for identifying *ortho*-phthalates either as a group, e.g., by Fourier-transform infrared spectroscopy (FTIR), or individually, e.g., by gas chromatography–mass spectrometry (GC–MS).^29^ While FTIR tends to have a higher limit of detection (LOD), in contrast to GC–MS it is fast, portable and comparatively cheap,^29−32^ making it suitable for testing end-of-life floorings. Once identified, contaminated waste streams need to be conveyed to an alternative treatment, such as incineration, which limits the recycling rate. A technology allowing selective removal of plasticizers while recovering the PVC polymers is solvent-based recycling, although technical development is still needed for implementing complete separation of phthalates from PVC on a large scale.^29,33−38^ So far, it has not been quantitatively assessed to what extent these identification and treatment techniques may enhance the recycling of phthalate-containing PVC waste.

In the present study, we investigate to what extent and with which accompanying measures PVC flooring recycling may be increased without causing negative health impacts due to exposure to hazardous legacy additives. To achieve this, we conducted a dynamic substance flow analysis for selected plasticizers that have been used in PVC flooring, looking at the Swiss market during the time period from 1950 to 2100. We quantify the uncertainty of the model results by considering different realistic input parameter values in scenarios combined with Monte Carlo simulations. We cross-check our model using (1) the measured concentrations in samples representative for the Swiss market in 2021/2022 that we determined in a previous study^29^ and (2) the substance flows in Germany from another dynamic substance flow model. This allows us to make more robust forecasts of future substance concentrations for different scenarios of increased recycling considering a potential removal of phthalates.

## 2 Materials and Methods

### 2.1 Substance Scope of the Analysis

The consumption and recycling behavior of the following plasticizers in PVC flooring was investigated for the Swiss market: di(2-ethylhexyl) phthalate (DEHP; CAS number: 117–81–7), diisononyl phthalate (DiNP; CAS number: 68515–48–0), and di(2-ethylhexyl) terephthalate (DEHT; CAS number: 6422–86–2).

This choice of plasticizers was based on their mass-wise relevance, confirmed or suspected hazards, and legal restriction status: DEHP, previously widely used,^5^ remains present in PVC flooring waste due to the long lifespan of flooring, despite banned for use without authorization since 2015 in the European Union and Switzerland due to its toxicity for reproduction and endocrine disrupting properties.^12,13^ DEHP has been mainly substituted by another *ortho*-phthalate, DiNP,^5,17,29^ which is currently subject to regulatory evaluation by the European Chemicals Agency (ECHA) regarding its endocrine disrupting as well as persistent, bioaccumulative and toxic properties.^17^ DEHT, a *tere*-phthalate, considered a suitable alternative with yet no identified concerns^17^ and a similar functionality as DiNP in flooring,^17,29^ has become a main alternative plasticizer.^17,29^

### 2.2 Model Description

To model the consumption and recycling situation for the selected plasticizers in PVC flooring on the Swiss market, a dynamic material and substance flow analysis^20,21,23,39^ was conducted. The model, implemented in Python, calculates the consumption, waste, and recycling amounts of the material PVC flooring and the substances DEHP, DiNP, and DEHT from the year 1950 until the year 2100 (section S1.2).

Lifetime distributions for PVC flooring were used to calculate waste amounts over time from consumption amounts. Therefore, PVC flooring waste arising in a year is composed of different age cohorts with individual compositions. Waste arising in a year was assumed to be recycled according to temporally changing recycling rates and the secondary material was assumed to be put on the market in the following year. The recycling situation was modeled as a closed system, although, in practice, secondary material from Swiss PVC flooring waste is partially utilized abroad and in other products than flooring, and the secondary PVC used in Swiss flooring partially originates from PVC waste from other countries and products.^3,40−42^ The closed-system modeling entails the assumption that the secondary PVC used in Swiss flooring has the same composition as the secondary material produced from Swiss flooring waste. This was considered plausible due to an at least Europe-wide similar market and recycling situation and the assumed minor use of secondary PVC from other products in flooring (section S1.3.3).

#### 2.2.1 Input Data and Uncertainty Consideration

The uncertainties in the input data of the model (Tables 1 and 2) were taken into account by two means. For most uncertain parameters, different values were investigated in scenarios, allowing to connect results to specific parameter values. By contrast, for PVC flooring consumption amounts, which underlie all substance flow scenarios, a probability distribution was considered via a Monte Carlo analysis running 1,000 simulations (section 2.2.2). This allowed us to show results variations related to uncertainties in flooring consumption within the substance flow scenarios assessed. The average plasticizer concentration in PVC flooring, used as an input to the substance flow analysis, was also modeled as a distribution function within a Monte Carlo simulation (section 2.2.3). The assumed variance of this concentration is small (section 2.2.3). Therefore, different concentration scenarios would have led to similar results, and we used the same concentration distribution in all assessments instead.

**Table 1:**
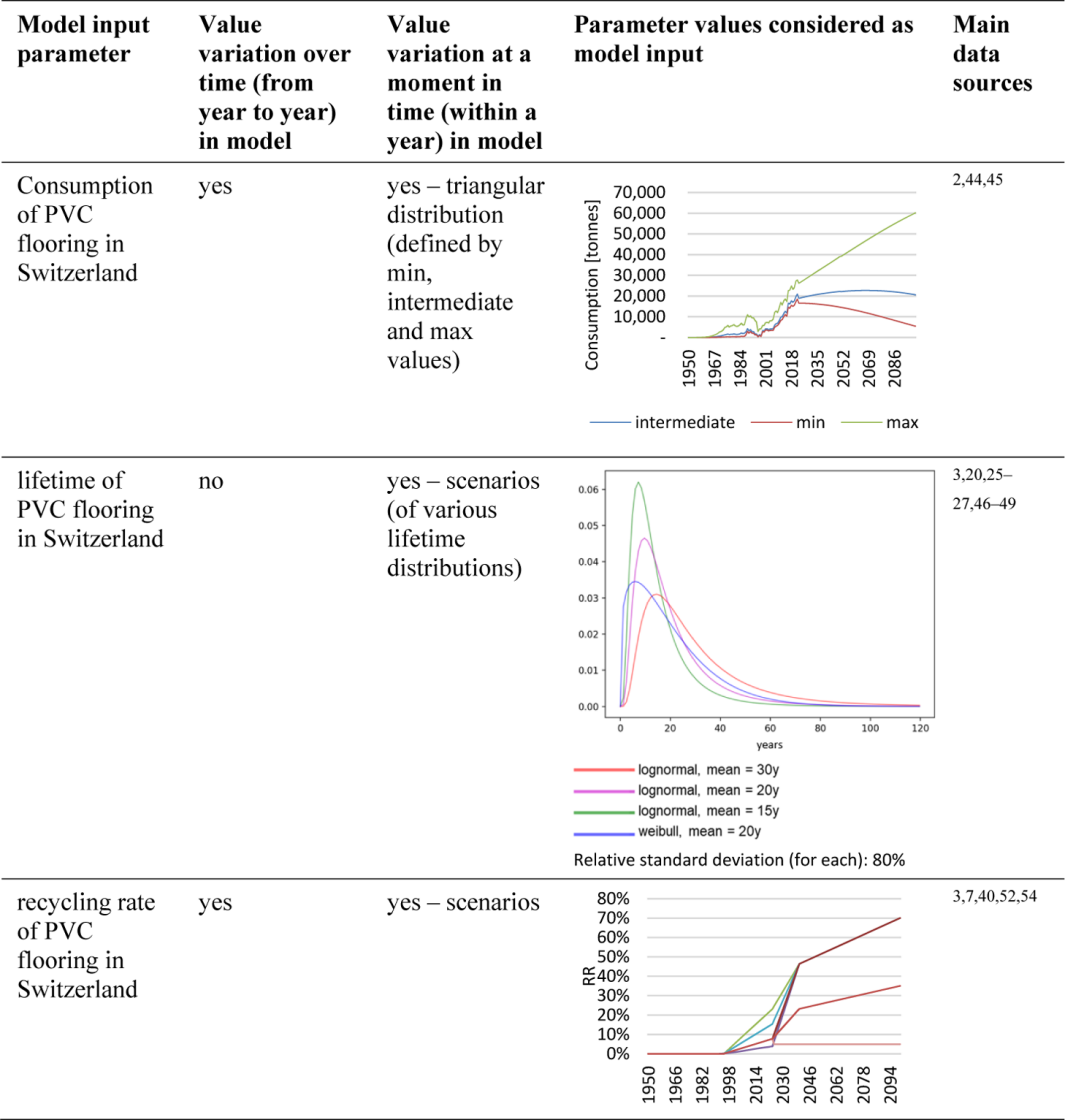
Model Input Parameters for the MFA^a^

a The values are provided in the Supporting Information.

**Table 2:**
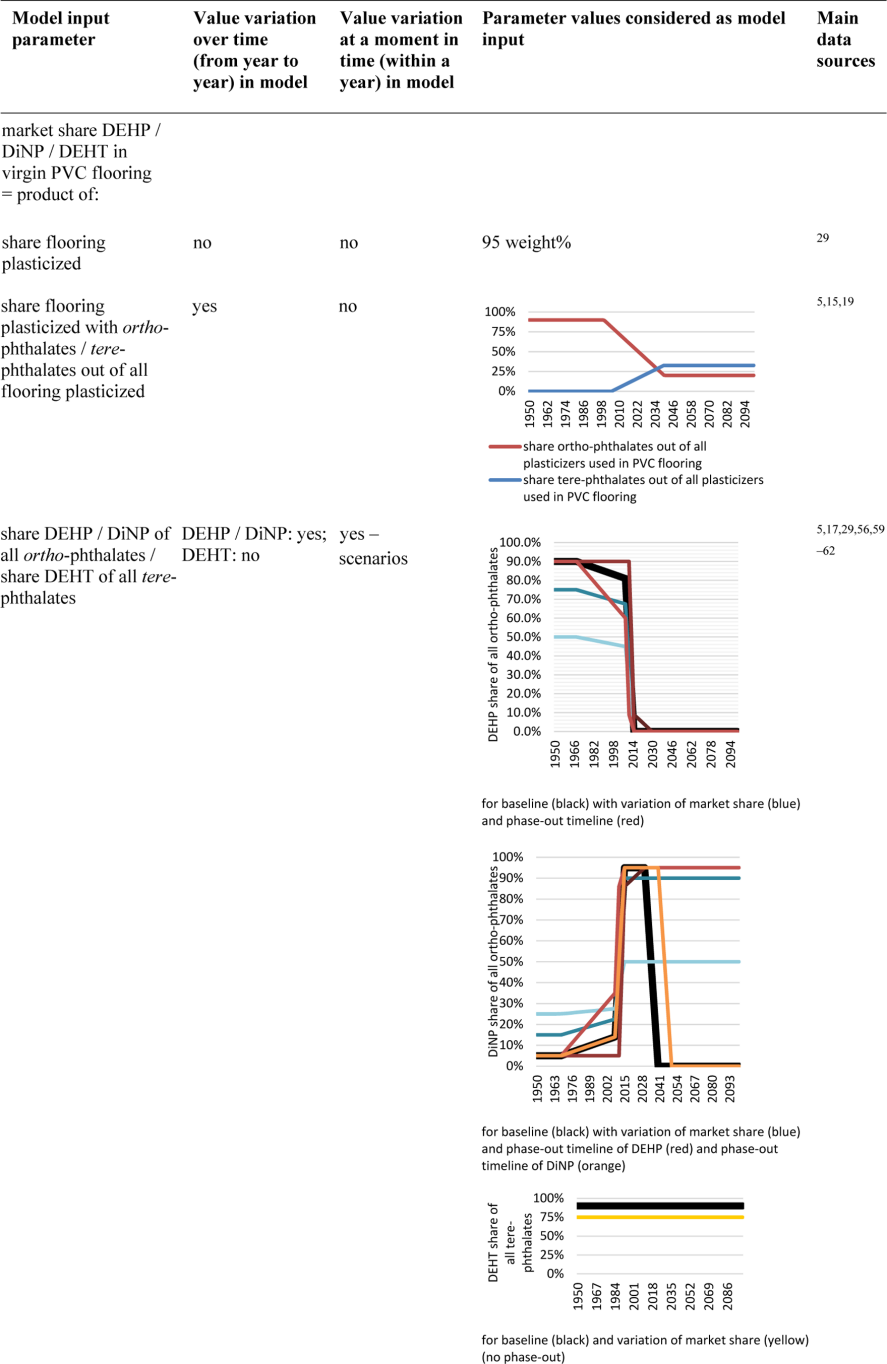

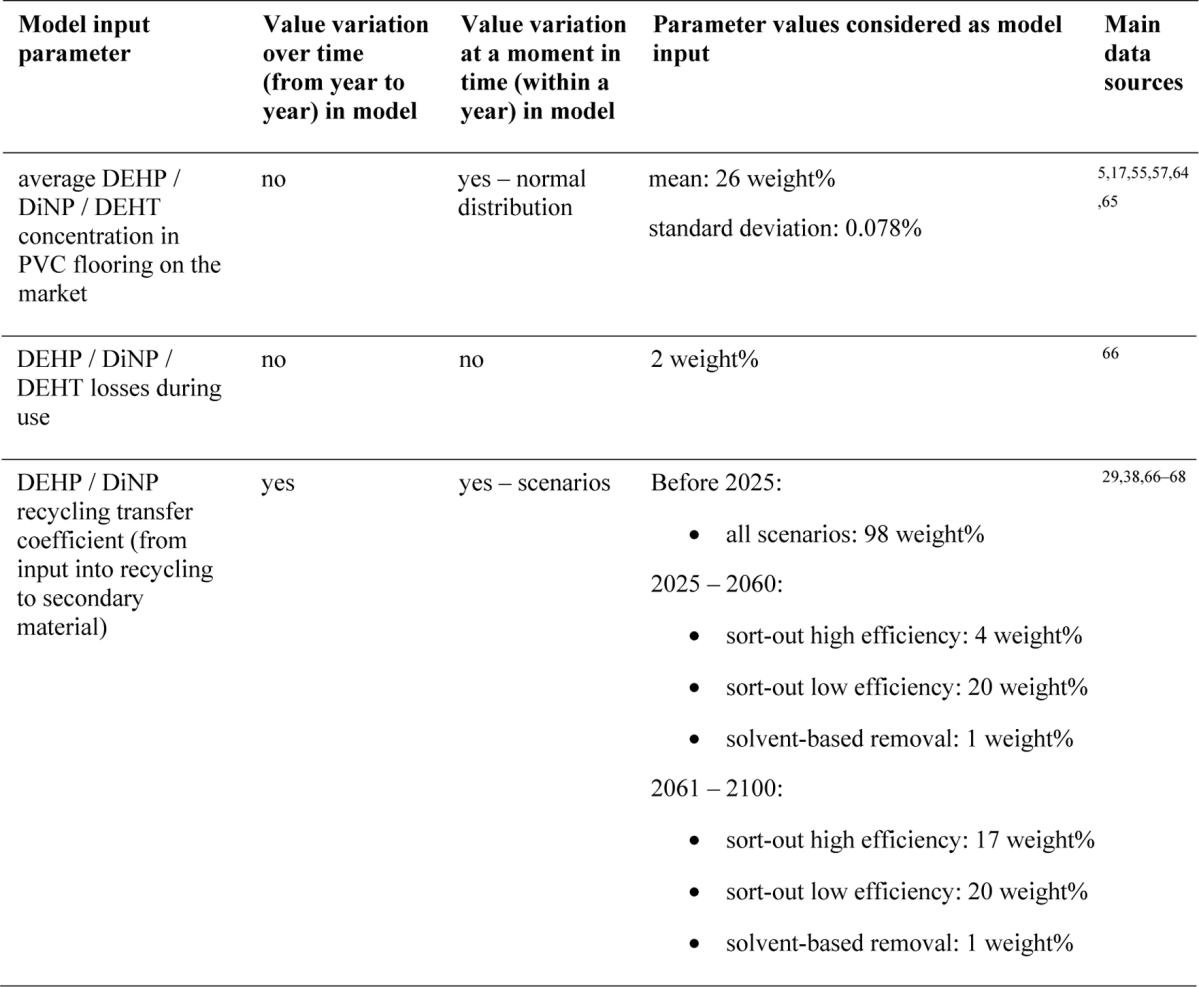
Model Input Parameters for the SFA^a^

a The values are provided in the Supporting Information.

#### 2.2.2 Material Flow Analysis (MFA)

For estimating consumption amounts of PVC flooring in Switzerland, different approaches were used for different time periods related to data availability (for details, refer to section S1.3.1). For the early modeling period between 1950 and 1987, Swiss PVC flooring consumption amounts were estimated based on a downscaling of the European PVC floor and wall covering consumption amounts.^43^ For the recent past (1988–2022), the trade data^44^ were used to estimate the Swiss production and net import amounts of PVC flooring, which sum up to the Swiss consumption. The PVC flooring production in Switzerland was estimated from the PVC net import and domestic production amounts over time. In doing so, we considered PVC and flooring production plant capacities over time, the shares of other PVC flooring components, PVC flooring gross export amounts, and the market share of imported floorings. For the prospective modeling period (years 2023–2100), the consumption amounts were projected based on constant, increasing, or decreasing per-capita consumption in the future along with scenarios of Swiss population development.^45^ Hereby, we aimed at showing the possible directions of development based on presently existing partially opposing trends. Over the whole model period, for the consumption amount of each year we estimated a high, an intermediate and a low value, based on which we modeled a triangular distribution (Table 1).

The waste amounts in individual years were calculated from the past consumption amounts and the lifetime distribution of PVC flooring^3,20,25–27,46–49^ (section S1.3.2). A share of the arising waste is recycled. The recycling amounts were calculated as the product of waste amount and recycling rate (RR). The latter was defined as the amount of secondary material used in PVC flooring on the Swiss market in relation to the Swiss PVC flooring waste amount, i.e., as the True Recycling Rate according to Klotz et al.^50,51^ The recycling rate varies over time, and we estimated trajectories based on the following points in time (details are provided in section S1.3.3). We assumed that no considerable amount of secondary PVC was used in the Swiss flooring before 1996, when the collection system for Swiss PVC flooring waste was established,^52^ which aligns with the European trend of PVC flooring recycling rates increasing after the year 2000.^42,53^ The RR in 2017 was estimated based on the recycling amounts of PVC flooring in that year,^40,54^ and the PVC flooring waste amount from the model, but we also considered scenarios of lower or higher RR values (Table 1). For the future, a maximum recycling scenario was modeled, based on an estimate of the maximum RR achievable for PVC flooring in the midterm future (46 wt% in 2040)^7^ and projecting a further potential RR increase up to 70 wt% in 2100 due to technical and socio-economic developments. We ascertained that the maximum recycling rates are compatible with the dismantlable share of end-of-life floorings and the viable recycled content in flooring (section S1.3.3). Besides, the recycling rates envisioned may be compromised by the measures for contaminant removal investigated in this study (section 2.2.3). Hence, to ensure scenario integrity and feasibility, we investigated the potential effects of the different contaminant removal strategies on the achievable recycling rates (sections 3.3 and S1.4.4). In addition to the maximum recycling scenario, we considered a scenario in which RRs would equal only half of the maximum scenario values. We also took into account a scenario of a constant RR of 5 wt %, similar to its estimated value today.

#### 2.2.3 Substance Flow Analysis (SFA)

The consumption amounts of plasticizers were estimated in relation to PVC flooring as follows (details in section S1.4.1). The largest share of PVC flooring is plasticized.^29^ The market shares of *ortho*- and *tere*-phthalates of all plasticizers were mainly based on data for the European plasticizer market from 2005 to 2020.^15^ Before 2005, *ortho*-phthalates dominated,^5,55^ and no *tere*-phthalates were assumed to be used.^15^ For the future, a continuation of the trend of decreasing *ortho*-phthalate and increasing *tere*-phthalate use^56^ was assumed, with constant shares after 2040.

The shares of DEHP and DiNP within the *ortho*-phthalates group were estimated considering that DEHP was predominantly used in the past^5,57^ (assuming 50–90 wt% of all *ortho*-phthalates before start of phase-out) and DiNP was the second most important *ortho*-phthalate^5,56,58^ (assuming shares of 20–3 wt% before start of DEHP phase-out). DEHP use was assumed to have slightly decreased after the first scientific findings regarding potential hazards in 1968,^59^ followed by a more severe decline after having been put on the EU candidate list of substances of very high concern (SVHC) in 2008.^60^ The major shift to alternative plasticizers was assumed to have happened between addition of DEHP to the EU REACH authorization list in 2011^61^ and the sunset date in 2015.^62,63^ Different phase-out scenarios with higher or lower degrees of reduction in the earlier and later phases were considered (section S1.4.1). This included a scenario in which 10 wt % of virgin floorings on the Swiss market still contained DEHP in 2015, with a reduction of this noncompliant market share to ultimately zero in 2030. The assumed potentially continued presence of DEHP in virgin floorings after sunset was based on the high efforts related to effective market surveillance, especially as 95% of Swiss floorings are imported.^3^

DEHP was mainly substituted by DiNP,^5,17,29,57^ the use share of which within the *ortho*-phthalates group, therefore, increased over time (making up for one-third of the share reduction of DEHP in the model). Given that DiNP also shows some reason for concern,^17^ scenarios of future phase-out of DiNP were considered. DEHT is the most common *tere*-phthalate^29,56^ (assumed shares of 90–75 wt%). Up to now no potential hazards have been identified,^17^ so no phase-out was modeled for DEHT.

The concentration of plasticizers in PVC flooring was based on typical formulations^5,55^ (section S1.4.2). The range of possible concentrations was determined considering the varying shares of plasticizers as well as fillers in PVC, which constitute a majority of the total flooring mass.^5^ The estimated average plasticizer concentration of 26 wt% is consistent with previous studies^25,42^ and measurements for the Swiss market.^29^ The average concentration of phthalates in flooring on the market was assumed to be normally distributed with a small standard error, based on the central limit theorem.^64^ While the concentration range was estimated for DEHP, the same values were assumed for all the three plasticizers, because both DiNP and DEHT can substitute DEHP on an close to one-to-one basis due to the similar properties and densities of these three plasticizers.^17,57,65^

The losses of plasticizers to the environment during use and recycling were quantified based on Fantke et al.^66^ (section S1.4.3). The plasticizers contained in secondary materials are transferred to products made from the latter. However, two ways of removing DEHP and DiNP from recycling streams were considered (section S1.4.4). One operational way is to identify phthalate-containing waste with FTIR and to convey it to an alternative treatment. The related maximum removal efficiency (high-efficiency scenario) was estimated based on an FTIR sensitivity of 97.2% associated with a limit of detection (LOD) corresponding to the legal limit for DEHP of 0.1 wt%^12,29^ (Table 2). The resulting removal efficiency varies over time as it depends on the concentration and distribution of phthalates in flooring waste. In a low-efficiency scenario, we assumed that not all waste is tested for contained phthalates. Another potential future option is selective extraction of phthalates from PVC flooring waste by means of solvents, which has achieved high removal efficiencies in lab-scale facilities.^38,67,68^

### 2.3 Model Cross-Check

The model results were cross-checked to, on one hand, address the issue of input data gaps and select only plausible values for model inputs (section 2.3.1). This can contribute considerably to an accurate representation of material flows, as shown by Buchner et al.^69^ On the other hand, we investigated the geographical validity of our results by comparison with another European country (section 2.3.2).

#### 2.3.1 Comparison of Modeled and Measured Concentrations for the Swiss Market

Quantitative concentration data for DEHP and DiNP in PVC flooring samples from the Swiss market in 2021 and 2022 were available from chemical analyzes conducted by Wiesinger et al.^29^ Based on the measurement data, we calculated a weighted average concentration for the Swiss market (section S1.5.1). Against this concentration, we compared the average concentrations of DEHP and DiNP in PVC flooring for the years 2021 and 2022 estimated with the model using different input parameter combinations. For the future scenarios, we only considered system configurations leading to a mean DEHP concentration in PVC flooring lying within plus/minus two-third of the weighted average measured concentration for the years 2021/2022 (section 3.2.1).

#### 2.3.2 Comparison of the Model Results with Modeled Substance Flows in Germany

For investigating the wider applicability of the results from the Swiss model, a comparison with the flows of DEHP in PVC flooring in Germany was conducted (section S1.5.2). The German situation was modeled using the dynamic material flow model ODYM,^70^ which was adapted to allow for consideration of the recursive recycling dynamics. The model input data on the PVC flooring consumption, product portfolio (reflected in the plasticizer concentrations), and past recycling rates in Germany were different from those for Switzerland. For several other parameters, in contrast, there was no reason to assume differences between the two countries. These parameters include the lifetime distribution of PVC flooring, relative share of DEHP among plasticizers, plasticizer losses during use and recycling, and qualitative trends regarding future recycling rates. The related input data, in both models corresponded to the values from Tables 1 and 2.

## 3 Results and Discussion

### 3.1 Overview of the Material and Substance Flows

**Figure 1:**
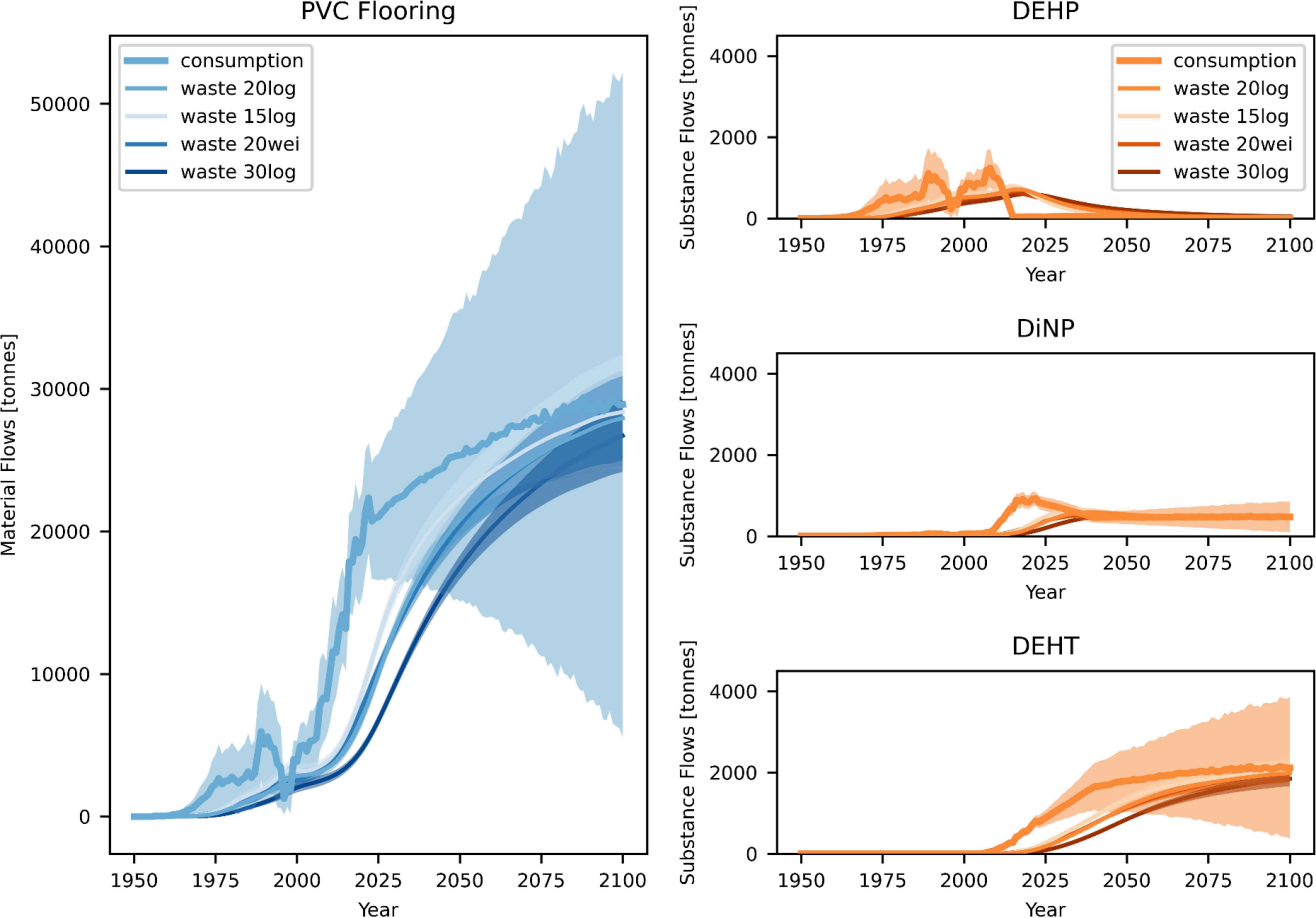
Overview of the material flows of PVC flooring as well as the substance flows of DEHP, DiNP, and DEHT in Switzerland. The waste flows are provided for different lifetime distributions (15log: log-normal distribution, mean = 15 years, 20log: log-normal distribution, mean = 20 years, 20wei: Weibull distribution, mean = 20 years, 30log: log-normal distribution, mean = 30 years). The substance flows are provided for the scenario with high market share and early phase-out of DEHP, with corresponding market share and no phase-out of DiNP, high market share of DEHT and low PVC flooring recycling rates in the past and future. The colored areas correspond to the mean flows plus/minus twice the standard deviation.

The PVC flooring consumption amounts in Switzerland have increased over time, following an exponential trend until today (Figure 1), similar to other plastic products.^71^ In the time period between 1990 and 1995, however, consumption temporarily declined, followed by the cease of PVC production in Switzerland.^72−74^ Subsequently, also two Swiss flooring manufacturing plants closed down around 1998,^74^ which is reflected in the flooring export data (Figure S2). The sharp rise in consumption after the year 2000 may be attributable to a trend favoring the use of vinyl flooring over other materials coupled with a general increase in wealth, while population growth was similar to that of the previous time period.

The relative uncertainty related to the flooring consumption amounts is higher in the more distant past than that for periods closer to today. This increased uncertainty is due to a lack of trade data as well as changes in the Swiss PVC flooring production landscape that made an accurate estimate for individual years difficult (section S1.3.1). Today, only a small share of all PVC floorings used in Switzerland are produced locally (approximately 5%),^3^ which is why it was possible to estimate the consumption amounts for recent years rather accurately via import statistics. The future scenarios range from a drastic reduction of PVC flooring consumption, a scenario that has been investigated by the European Chemicals Agency (ECHA),^17^ to a possible simultaneous increase in the Swiss population and per-capita PVC flooring consumption, which may lead to a tripling of today’s consumption by 2100.

DEHP consumption amounts increased along with the PVC flooring consumption but do not show the same steep increase after the year 2000, as during that time the overall phthalates market share was already decreasing (Table 2). Even after phase-out in 2015,^12^ DEHP remained present in PVC flooring on the market due to recycling, and concentrations are expected to yet increase in the case of increasing recycling rates (section 3.3).

DiNP, used in lower amounts than DEHP in the past, substituted for the latter when its consumption was reduced.^5^ Therefore, DiNP exhibited a sharp consumption increase between 2008 and 2015. Since then, consumption has decreased despite increasing flooring use amounts due to the falling market share of *ortho*-phthalates but will remain considerable in the future if no phase-out of DiNP occurs (Figure 1). Even in the case of a phase-out as early as possible (section S1.4.1), however, DiNP concentrations are expected to remain above the legal limit for DEHP until the end of the model period in the case of high future recycling rates (Figure S27).

DEHT, in contrast, was not used in the past, but has been increasingly utilized since 2005, as it currently shows no reason for concern.^17^ Consumption is expected to level out at some point along with the flooring material flow (Figure 1).

The waste amounts of floorings and plasticizers are each in a similar range for the different mean lifetimes and distributions considered. The reduced uncertainty in comparison to the consumption is due to compensation of the consumption fluctuations in positive and negative direction (section S2.1).

### 3.2 Validity of the Model Results Based on Cross-Checking

#### 3.2.1 Plausibility of the Model Results Based on Measured Concentrations for Switzerland

**Figure 2:**
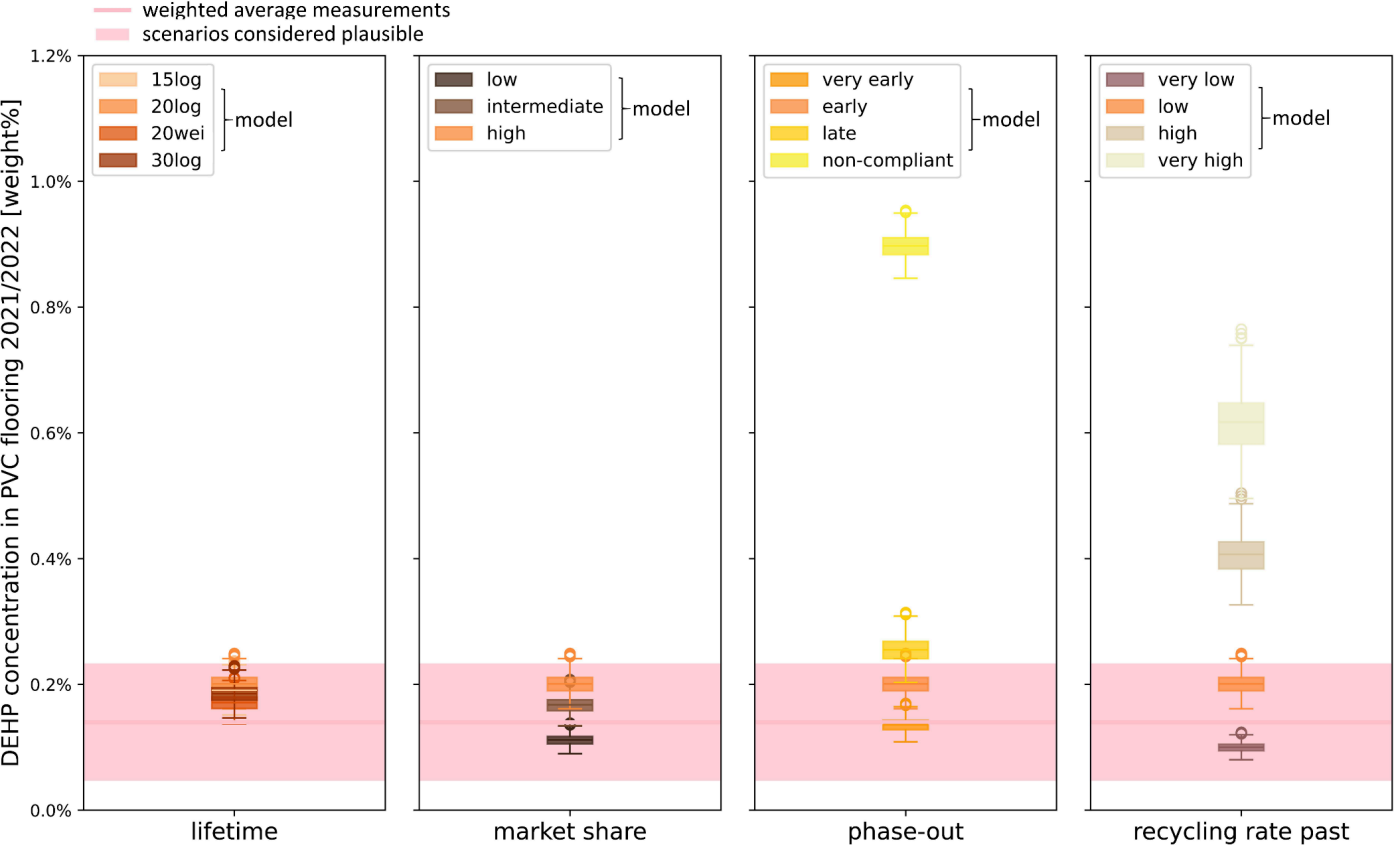
Modeled versus measured concentrations of DEHP in PVC flooring in Switzerland in 2021/2022. Departing from the best-estimate model configuration (log-normal lifetime distribution of PVC flooring with mean of 20 years, high DEHP market share, early DEHP phase-out, low past recycling rates of PVC flooring; orange in all subplots), one parameter was varied at a time. The exact parameter values for the modeled system configurations are available from sections 2.2.2 and 2.2.3. The shaded area depicts the concentration range considered plausible based on the measurement data, extending to plus/minus two-thirds of the weighted measurement average (section 2.3.1). 15log: log-normal distribution, mean = 15 years, 20log: log-normal distribution, mean = 20 years, 20wei: Weibull distribution, mean = 20 years; 30log: log-normal distribution, mean = 30 years.

The market average DEHP concentrations for the years 2021 and 2022 estimated with the model were close to the weighted average of measured concentrations (0.14 wt %) for most parameter configurations assessed (Figure 1, section S1.4.5). While the measurement data also entails some uncertainties due to incomplete sampling (though related to a much larger market share than is the case for most other studies),^29^ this validates the model assumptions, i.e., most input parameter value ranges considered appear plausible. For the assessment of future scenarios (section 3.3), only model configurations leading to realistic concentrations in 2021 and 2022 (shaded value range in Figure 2) were regarded.

It appears likely that the past recycling rate has been below 10 wt %; as for the “high” recycling rate scenario (recycling rate curve with a value of 12 wt % in 2017), the estimated DEHP concentration for the years 2021 and 2022 is about three times as high as the weighted average of the measurements (Figure 2). Recycling rates below 10 wt%, rather around 3 wt% (which would be similar to the “very low” scenario), are also in line with the estimate by the provider of the Swiss PVC flooring waste collection system.^3^ Still, even these minor past recycling practices were likely the reason for the average concentration of DEHP in PVC flooring on the Swiss market in 2021/2022 lying above the legal limit of 0.1 wt % (based on model and measurements). Another cause for this exceedance may have been the noncompliance of virgin materials, which, however, seems to have been limited: Based on the model, less than 10 wt% (section 2.2.3) of the floorings on the market were noncompliant, otherwise the DEHP concentrations in floorings would have been far higher than those measured. Phase-out appears to have occurred even earlier than legally required (Figure 2), which is in line with the information from manufacturers that in the European flooring production, phase-out of DEHP took place from the early 2000s.^75^ For DiNP, the model concentrations for 2021/2022 lie above the measurements, while they are still comparatively close (Table S2, section S2.2.2). This indicates that possibly DiNP substituted only for a lower proportion of phased-out DEHP and/or DiNP constituted only a lower share of all non-DEHP *ortho*-phthalates in the past than assumed in the model. It may also be that the plasticizer concentration in floorings has been slightly overestimated or decreased over time (section S1.4.2).

#### 3.2.2 Relevance of the Model Results on a European Scale

**Figure 3:**
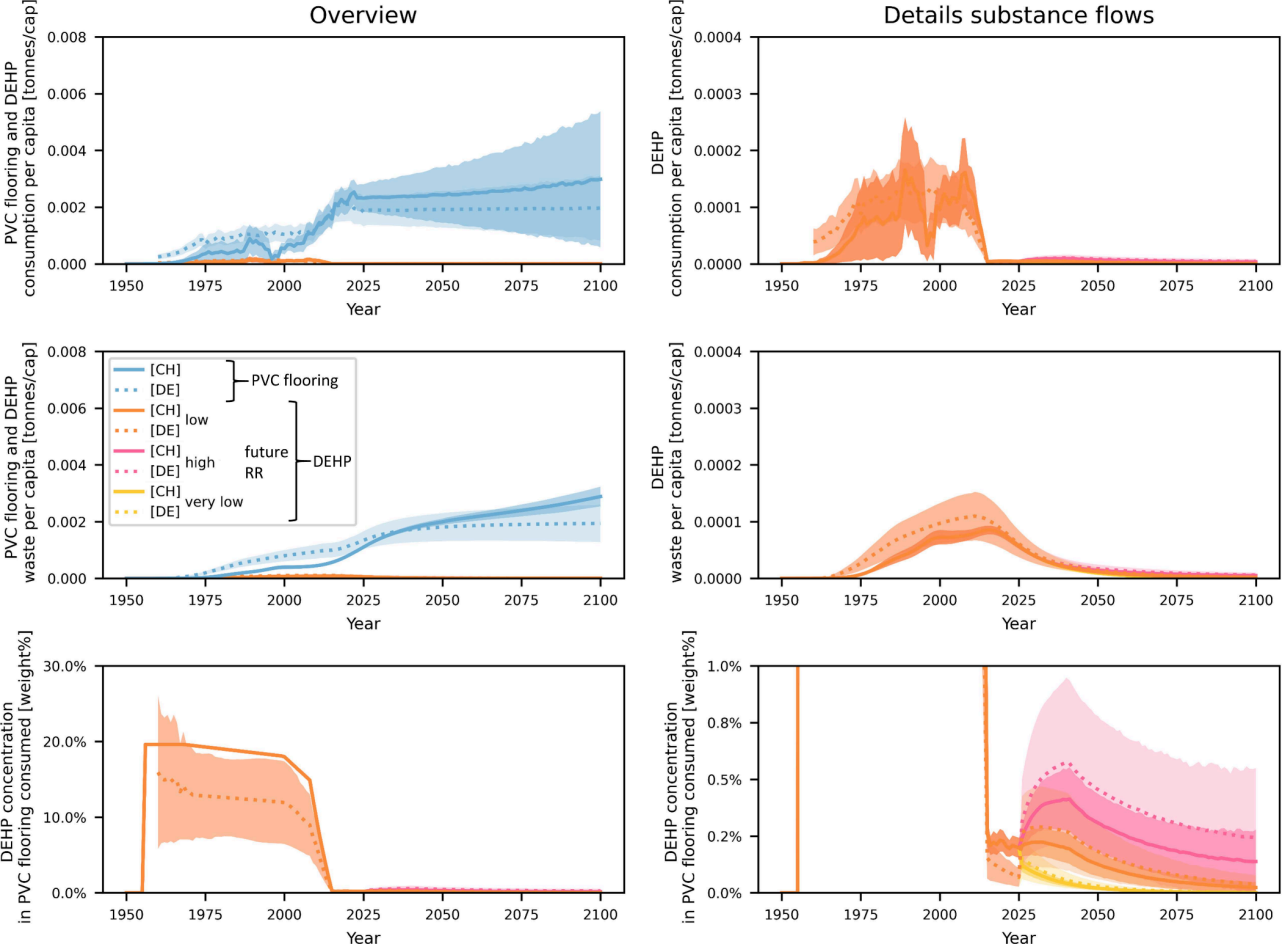
Modeled PVC flooring and DEHP consumption and waste amounts as well as DEHP concentrations in PVC flooring for Switzerland in comparison to Germany. The left and right plots in each row show the same substance flows, with the scaling in the right plots being larger. The shaded areas show the mean plus/minus twice the standard deviation. CH: Switzerland, DE: Germany, RR: recycling rate, cap: capita.

In comparison with the results of the German flow model, similar flow levels and trends can be observed for PVC flooring and DEHP in Switzerland and Germany. The per-capita consumption amounts of PVC flooring and DEHP estimated for Switzerland and Germany are close to each other for the entire time period assessed (Figure 3). In both countries, an initial growth in PVC flooring consumption was followed by a stagnation, with subsequent sharp increase until today. At the same time, the comparison indicates that the down and up of the Swiss consumption between 1990 and 2000, while to some extent representing the Swiss-specific production landscape, may partly be a model artifact owing to the difficulty of representing the dynamic Swiss PVC flooring production situation in that time period (sections 3.1 and S1.3.1). In the time period before 2010, the PVC flooring consumption in Germany tended to be higher than the one in Switzerland. This may have been due to a strong PVC industry in Germany, being one of the earliest industrial producers of this plastic.^1,76^ In the recent past, the per-capita consumption of the two countries has been very similar, with little uncertainty attached.

DEHP use per capita is in good agreement between the two country models, as the slightly higher PVC consumption estimates for Germany compensate for slightly lower DEHP concentration estimates. The German DEHP concentration is an average for different PVC flooring types (section S1.5.2), for which individual plasticizer shares were considered (9–20 wt%^77−80^). The Swiss model had a lower resolution, representing average PVC flooring (section S1.1) with an average plasticizer concentration of 26 wt%.

In the years after the phase-out of DEHP in both countries in 2015,^12,13^ its concentrations in floorings on the market were lower for Germany based on the models, which is because of the lower assumed recycling rates. Future DEHP concentrations, however, assuming the same trends in recycling (section 2.3.2), are very close to each other for the two countries. The slightly higher prospective concentrations in the German model are related to lower future PVC flooring consumption estimates combined with similar per-capita DEHP waste amounts as those for Switzerland.

Overall, the comparison of the two models shows similar per-capita flow levels and concentrations of DEHP in PVC flooring for both countries, with small differences related to deviations in the consumption patterns and recycling rates. This demonstrates the transferability of the results obtained for Switzerland to a neighboring country with similar consumption and recycling conditions (Germany). Likewise, the results may even be transferable to a broader European context in which, due to political alignments and a joint market, similar recycling situations may occur.

### 3.3 Future Scenarios

**Figure 4:**
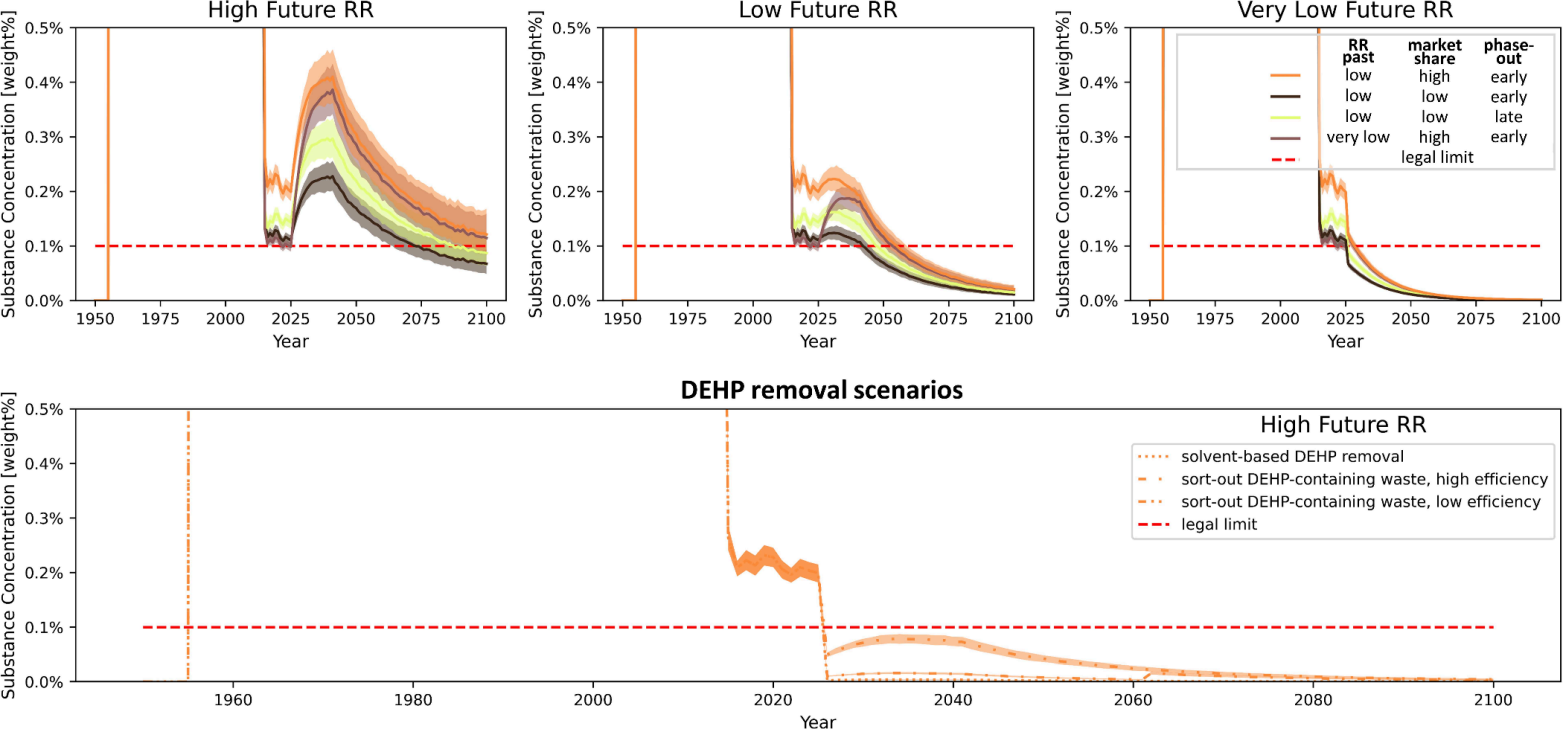
Future DEHP concentrations in PVC flooring in the case of different future recycling rates for selected plausible model scenarios (section 3.2.1). Three of the scenarios shown are also represented in Figure 2 and have the same colors in both figures, while an additional plausible scenario is included in this figure (green color). DEHP removal scenarios are shown for a high future PVC flooring recycling rate, for the scenario with a low past PVC flooring recycling rate, high DEHP market share, and early DEHP phase-out (best-estimate scenario; orange), i.e., for the scenario leading to the highest future DEHP concentrations of all selected scenarios. RR: recycling rate.

According to different plausible model scenarios (section 2.3.1, Figure 2), even after phase-out of DEHP in 2015, its average concentration in PVC flooring on the Swiss market has continuously remained above the legal limit of 0.1 wt%^12^ (Figure 4). The main influencing factors for the concentrations during the time after phase-out were the recycling rates in that period and the past DEHP market share.

**Figure 5:**
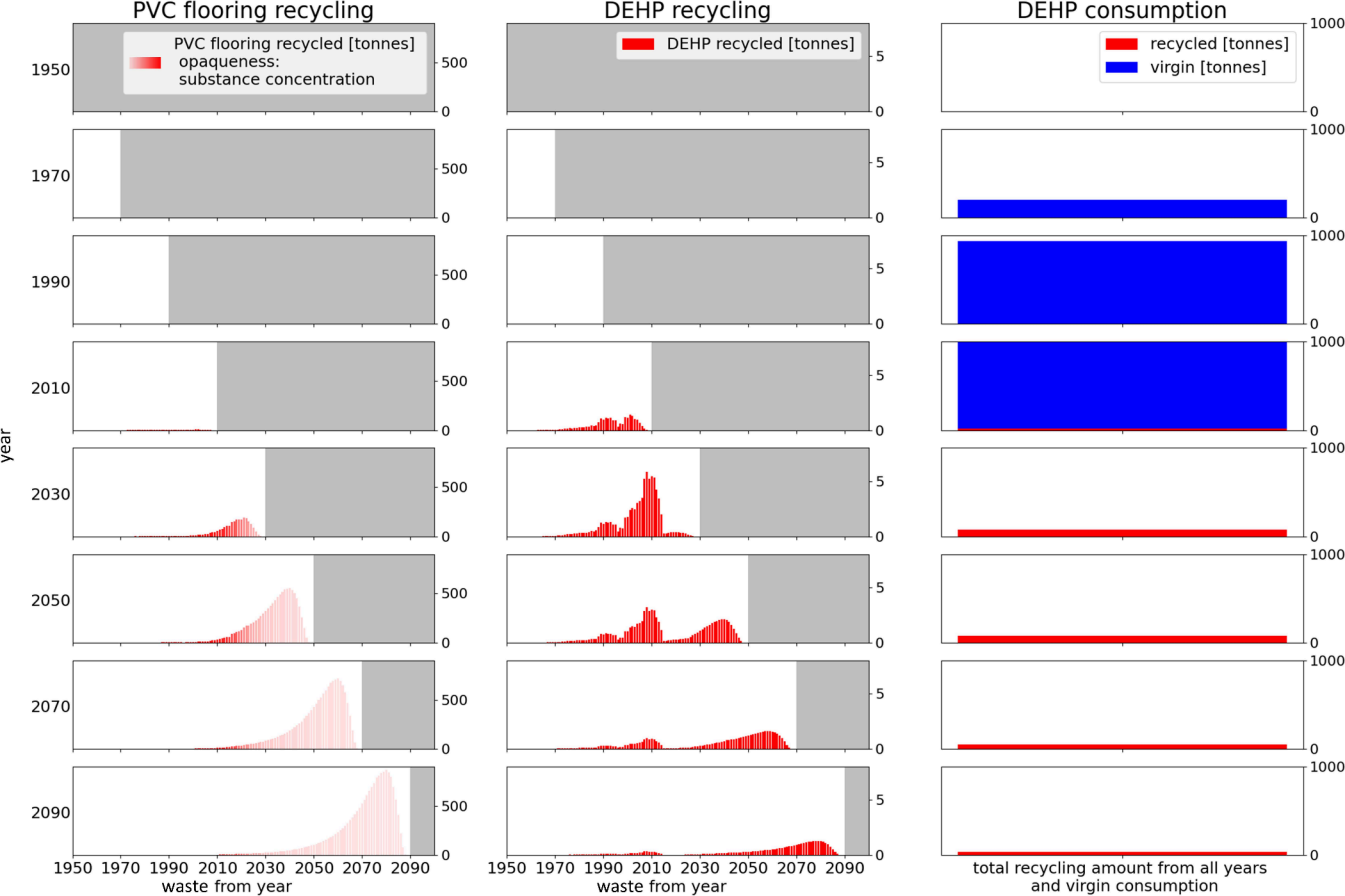
Insights into substance recycling mechanisms: Temporal origin and destination of recycling amounts of PVC flooring and DEHP over time and resulting total DEHP recycling amounts for a low recycling rate in the past and a high recycling rate in the future without substance removal at recycling. The opaqueness of the bars in the first subplots indicates the DEHP concentration in the recycled waste (higher opaqueness corresponds to higher concentration). The gray areas cover the time periods that are in the future relative to the reference year. For comparison, the same figures for a high recycling rate in the past and a low recycling rate in the future, as well as contaminant removal at recycling are shown in Figure S22.

The future DEHP concentrations are mostly determined by the future recycling rates along with the past DEHP market share. The cross-checked model shows that if we want to implement high recycling rates for PVC flooring in the future, the average DEHP concentrations will likely remain above the legal limit if no countermeasures are taken, potentially considerably and for a long time (upper left and center plots in Figure 4). For the scenario of maximum recycling rates, concentrations would considerably rise in the future compared to today, which is due to higher recycling amounts combined with waste still containing considerable shares of DEHP (Figure 5). However, even if the recycling rate in the future is lower, increasing to only 35 wt% in 2100 (section 2.2.2), concentrations are expected to exceed the allowed value until around 2050. Only for a constantly very low future recycling rate of 5 wt % would concentrations comply with the legal limit, in Switzerland and presumably similarly in other European countries (upper right plot in Figure 4, bottom right plot in Figure 3).

DEHP removal from recycling streams, however, could reduce its average concentration in floorings on the market to values below the legal limit already from 2025 onward, even at high recycling rates (lower plot in Figure 4). This applies regardless whether DEHP removal is achieved via simply sorting out contaminated waste or utilizing solvent-based recycling. For DiNP, removal appears sensible along with a phase-out and could, after elimination of DiNP in virgin material, quickly reduce its concentrations below 0.1 wt %, as well (Figure S27). Implementing the modeled removal scenarios in a recycling context^81^ seems feasible, as identification of contaminated PVC flooring waste with FTIR is already being practiced,^82^ with further trials^53^ and evaluations^83^ ongoing. Besides FTIR, further suitable analytical techniques may exist for identifying phthalates (section S1.4.4). Moreover, a pilot plant for solvent-based recycling of PVC flooring is under development, using the CreaSolv process, which in lab scale achieved phthalate removal efficiencies above 99.9 wt% and recovered PVC with mechanical properties allowing for reutilization in flooring.^37,38,82^ Solvent-based recycling will, however, probably not be practicable at full scale for the coming years, rendering the dependent scenarios rather relevant for time periods further in the future.

If contaminated flooring waste is conveyed to solvent-based recycling, then nearly the complete PVC amount may be recovered.^29,33−37^ Due to the removed phthalate amounts, higher but still feasible collection rates are required for achieving the target recycling rates of the scenarios (Figure S12). Recovered phthalates may potentially even be transformed into nonhazardous chemicals and utilized.^38^ However, the sort-out of complete floorings that contain phthalates is also theoretically compatible with high recycling rates, provided that early action is taken: Phthalates have only been used in a (decreasing) share of the floorings on the market (section 2.2.3). Therefore, the share of waste not containing phthalates may be sufficiently large for achieving the maximum recycling rate curve (Figure S21). The distribution of phthalates in waste, however, depends on recycling practices. If recycling distributes the phthalates contained in waste to a large number of floorings on the market, then a lot of flooring material needs to be sorted out later, lowering the recycling rates achievable. So far, while a wide range of recycled contents are common in floorings,^3^ dilution may still be limited as recycling rates were low in the past. For ensuring high phthalate-free waste shares in the future, it seems, therefore, of utmost importance to start removing contaminated waste from recycling as early as possible. In addition, phase-out of DiNP may be required (section S1.4.4).

In synthesis, the maximum recycling scenario from the model, being potentially compatible with contaminant removal, as well as with feasible collection rates and recycled contents (section 2.2.2), seems to be ambitious but theoretically achievable. High recycling rates are also supported by the political will in Europe, which is striving to foster a circular economy in the context of urgently needed greenhouse gas reductions.^84^ Increased plastic recycling rates are specifically aspired to,^85^ also particularly for construction.^86^ Still, the increase in recycling required by the maximum recycling rate scenario is drastic. What argues against such a strong increase are prevailing challenges related to postconsumer PVC recycling. While issues related to contamination from glue or cement are expected to decrease with the increasing use of glueless installation methods,^87,88^ challenges such as the identification of varying material compositions and a lack of profitability are likely to persist.^89^ Against this background, future targets for the recycled content in PVC flooring often rely on the use of recycled preconsumer waste or other recycled materials, such as calcium carbonate used as a filler.^53^ Therefore, the scenario of low future recycling rates may be more probable to occur.

### 3.4 Implications

To avoid a burden shift from climate change to health impacts when increasing the recycling of long-lived products contaminated with legacy substances, such as PVC flooring, only clean materials should be recycled.^90^ This requires that either (a) hazardous additives are removed from recycled waste streams or (b) the entire material stock is clean, which can be ensured by strictly regulating the virgin materials employed:

a. Increasing the PVC flooring recycling rates in a European context without taking measures to remove legacy plasticizers is expected to lead to DEHP concentrations above the legal limit of 0.1 wt% for several decades in the future. Fast chemo-analytical methods for screening PVC flooring waste streams for DEHP exist and can be implemented in the recycling context. It is crucial to employ these methods as early as possible for removing contaminated waste, to avoid a dispersion of legacy additives to a wide range of products by increased recycling. An alternative would be direct removal, e.g., by means of solvents, of legacy substances in product waste, the feasibility of which, however, is highly uncertain due to its limited technology readiness level (5–6).^91,92^ While for *ortho*-phthalates suitable screening techniques exist, not all concerning substances can be detected in a quick and reliable manner. For instance, organotins are widely utilized in PVC flooring as stabilizers and several are known to be neurotoxic and/or endocrine-disrupting at very low concentrations.^93,94^ However, with screening techniques like X-ray fluorescence, only the tin content of samples can be detected and only at comparatively high concentrations, while the presence of specific hazardous organotins at low levels cannot be ascertained.^29,94^ Thus, the potential presence of such substances may render efforts for contaminant identification and removal too high for an environmentally and economically efficient recycling.
b. A safe material stock (within a product category) could be required as part of regulations that aim at increasing material circularity, e.g., the European Circular Economy Action Plan within the Green Deal.^84^ Considering the precautionary principle,^95^ regulatory requirements and concentration limits (as existing for DEHP) should extend to substances with known hazards (such as DiNP) and those with insufficient hazard data. However, the PVC flooring industry can also show self-responsibility by aiming at ensuring a safer material stock through agreement on a positive list of nonhazardous additives to be used. This has, for example, been implemented for PET bottle recycling.^96^ For such an industry cooperation, sufficient similarity between the material qualities required by different product manufacturers is needed. PVC flooring used in Switzerland is largely produced abroad and recycled separately from other products but together with PVC floorings from other European countries. Therefore, cooperation and harmonization of products would have to occur at least on the level of the European market and include all major producers. The European PVC industry has already shown the ability to closely cooperate, e.g., in the transition away from lead- and cadmium-based stabilizer systems,^93,97^ and thus other concerning substances such as DiNP may be proactively restricted.

Besides phthalates in PVC flooring, several further legacy additives are known to be present in plastics waste.^98^ Considering this, and given the generally very limited knowledge about the identity and properties of additives used in plastics,^99^ the recycling challenges posed by hazardous additives found in plastic waste, as exemplified in our analysis of plasticizers in PVC flooring, could potentially apply to a wide range of plastic products. Prior to increasing plastic recycling rates, we must, therefore, ensure that recyclates are clean or contaminants are removed.

The trade-off between climate benefits and health impacts of recycling extends beyond the realm of plastics. Paper products, for instance, similar to plastics, consist of a matrix material (fibers) complemented with substances fulfilling certain functions such as coloring, through which hazardous components happen to be introduced.^23^ Paper waste can also contain DEHP, introduced via inks or glues, besides other contaminants such as bisphenol A and mineral oil hydrocarbons.^23^ For reducing the presence of hazardous substances in new paper products, similar measures were proposed as in this study. Phasing-out contaminants was identified as the most effective measure, which alone, however cannot prevent further contaminant presence due to recycling over several decades.^23^ This aligns with the findings of this study, where the rate of concentration reduction is similar. The reason for this is that the average lifespan of paper products is shorter than that for PVC flooring, while recycling rates are higher. Removing pollutants by sorting out contaminated waste is only compatible with high recycling rates when contaminants are narrowly distributed among products, which is less likely the case if products contain different contaminants.^23^ Deinking of paper, in contrast, analogous to solvent-based PVC recycling, enables simultaneous contaminant removal and material recycling.^23^ It does, however, also reduce the recycling yield to a certain degree.^23^ Metals, as well, usually contain other elements besides the main carrier material (e.g., nickel in steel or magnesium in aluminum), providing for specific material properties such as increased strength or corrosion resistance.^100,101^ Alloy elements may be released, depending on the environment, composition, or surface finish, partly in concerning levels and forms.^102,103^ Some hazardous elements were used in the past in connection with metals, such as hexavalent chromium used for plating^104^ or lead used in metal paints,^105^ and have in the meantime been restricted.^12^ Still, health impacts have so far been a subordinate recycling concern, while studies focused on how these additive substances compromise recycling in several other ways.^100,106,107^ Wood products may be contaminated by organic or inorganic substances, such as polychlorinated biphenyls contained in floor finishes, polycyclic aromatic hydrocarbons used in adhesives for particleboards or heavy metals present in wood preservation agents.^108,109^ The trade-off between quantity and quality of recycled wood has been highlighted.^108^ Removing contaminant-containing wood wastes from recycling streams intended for particleboard production is being practiced, using contamination classes, while advanced separation by means of, for instance, labeling has been suggested.^108^ In synthesis, while each material group shows specific properties regarding composition and compound, deviating hazardous waste streams from recycling and substituting hazardous substances in the first place are generally applicable principles for enabling a circular economy while avoiding health impacts.

## Supporting Information Available

Details on methods, additional results (consumption, waste and concentrations for all plasticizers in different scenarios) and further discussion of uncertainties (PDF)
Model input data (XLSX)
