# Supplementary material for: Increasing the Recycling of PVC Flooring Requires Phthalate Removal for Ensuring Consumers’ Safety: A Cross-Checked Substance Flow Analysis of Plasticizers for Switzerland
Source: Environ Sci Technol. 2024 Oct 7;58(42):18686–700. doi: 10.1021/acs.est.4c04164 (PMC11500398; doi:10.1021/acs.est.4c04164)
Supplement: Supplementary file 1 — es4c04164_si_001.pdf [file es4c04164_si_001.pdf]

Supporting Information File 1

to

Increasing the recycling of PVC flooring requires phthalate removal for ensuring consumers' safety: A cross-checked substance flow analysis of plasticizers for Switzerland

Magdalena Klotz, Sarah Schmidt, Helene Wiesinger, David Laner, Zhanyun Wang, Stefanie Hellweg

Number of pages:	47
Number of figures:	28
Number of tables:	2

Contents

S1 Detailed methods description ... 3
S1.1 Materials ... 3
S1.2 Computation model design and implementation ... 3
S1.3 Material flow analysis (MFA) ... 4
S1.3.1 Consumption ... 4
S1.3.2 Waste ... 10
S1.3.3 Recycling rate ... 12
S1.4 Substance flow analysis (SFA) ... 14
S1.4.1 Market shares ... 14
S1.4.2 Concentration ... 17
S1.4.3 Plasticizer losses during use and recycling ... 18
S1.4.4 Contaminant removal ... 19
S1.4.5 Scenario overview ... 28
S1.5 Validation ... 29
S1.5.1 Comparison measurement data ... 30
S1.5.2 Comparison Germany ... 31
S2 Further results and discussion ... 33
S2.1 Further MFA uncertainty discussion ... 33
S2.2 Validation ... 34
S2.2.1 Measurement comparison DEHP ... 34
S2.2.2 Measurement comparison DiNP ... 34
S2.3 Insights into recycling patterns ... 35
S2.4 Further results for other substances ... 38
S2.4.1 DiNP ... 38
S2.4.2 DEHT ... 41
S3 References ... 42

S1 Detailed methods description

S1.1 Materials

We considered three plasticizers – di(2-ethylhexyl) phthalate (DEHP), diisononyl phthalate (DiNP) and di(2-ethylhexyl) terephthalate (DEHT) – used in PVC flooring as described in the main paper.

Regarding PVC flooring, the following types are common: homogeneous vinyl flooring, heterogeneous vinyl flooring, luxury vinyl tiles and cushioned vinyl flooring[1]. We did not differentiate between different flooring types for the following reason. While plasticizer concentrations in individual flooring types[2–5] and consumption shares of these flooring types[6] were known, usage patterns of different plasticizers in individual flooring types were not known. The latter may also play an important role regarding the usage of individual plasticizers in individual flooring types, which is why we decided that the data availability was not sufficient for increasing the level of detail.

S1.2 Computation model design and implementation

The model used for computation was implemented in Python and follows a similar logic and structure as the Open Dynamic Material Systems Model ODYM[7]. The material flow amounts of PVC flooring refer to average PVC flooring on the Swiss market including all additives contained. The shares of plasticizers contained in PVC flooring used for the calculation refer to the total material amount of PVC flooring containing all additives. The time frame starts from the year 1950, when the mass production of PVC began to ramp up[8,9]. By modelling the substance flows until the year 2100, both mid- and long-term future situations can be regarded. Virgin material consumption was calculated as the difference between the estimated PVC flooring consumption over time and the available recycling amounts. Production losses were neglected. This does not affect the concentrations of plasticizers in products, which were the scope of the analysis, while production losses (and their recycling) would be relevant if environmental impacts were to be assessed.

We conducted mass balance checks for the material and substances assessed by using test cases in which consumption ceases early enough so that the whole material and substance amounts consumed arise as waste within the model time period. For the substances, the losses during the use phase (Section 2.2.3 of the main paper) had to be considered.

We tested the model functionality with extreme parameter value configurations (Section S1.4.5, “implausibly high” / “implausibly low”), for which, as to be expected, the concentrations calculated with the model were far off from the plausible concentrations based on measurement comparison (Section S2.2.1). In addition, we modelled a scenario involving a fixed PVC flooring lifetime (instead of a lifetime distribution), resulting in waste amounts corresponding to the consumption amounts simply shifted towards the future, with the same uncertainty.

The calculation model was implemented in Python. The packages used for the calculations, the connection to the database with the input data, parallel computation and results plotting include numpy, pandas, scipy, sqlalchemy, multiprocessing and matplotlib. The dynamic

substance flow model developed can be flexibly used to investigate other materials and substances. It is available under *https://github.com/ecological-systems-design/sfa_flooring*.

S1.3 Material flow analysis (MFA)

In the following, the derivation of the values for the three MFA input parameters over time, namely consumption, waste and recycling rate of PVC flooring, is described.

S1.3.1 Consumption

Overview

Consumption of PVC flooring over time was considered assuming a triangular distribution, the minimum, peak and maximum values of which are given in Figure S1. The underlying data are provided in the SI2 file. The derivation of the distribution parameters is described in the following.

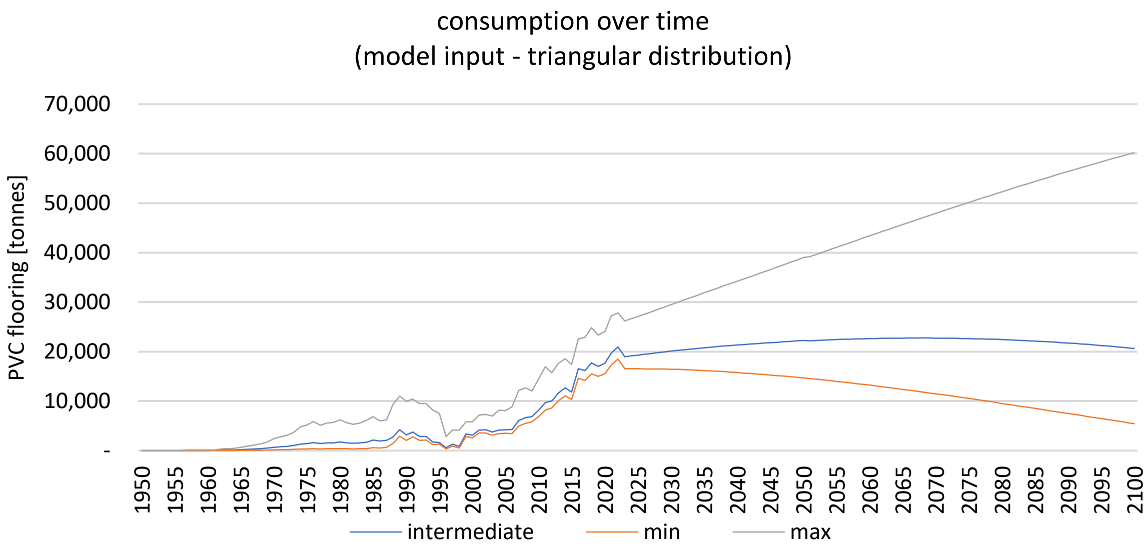

Figure S1: Minimum, maximum and peak values of the triangular distribution assumed for consumption of PVC flooring in Switzerland over time

Model period from 1950 to 1987

The data basis for the model period between 1950 and 1987 were values for the consumption of floor and wall coverings made of PVC in Europe[10]. These were scaled down to Switzerland based on the relation of the consumption of PVC in building and construction applications in Switzerland compared to Europe in 2014[11], amounting to 2%, which is in line with the relation of the population numbers in the two regions, also amounting to 2% in 1990[12,13]. For the peak value of the triangular distribution over time (best estimate), we considered this ratio for scaling down the European values, along with the median consumption value from Kawecki et al.[10]. For the maximum and minimum values of the triangular distribution, we considered the upper and lower quartiles of consumption from Kawecki et al.[10], respectively, along with a ratio of Swiss to European consumption of 4% and 1%, respectively, i.e. double or half as high as in

the best estimate. We estimated that flooring constitutes 67–80% of the consumption values from Kawecki et al.[10] referring to floor and wall coverings, based on PE Europe GmbH et al.[14]. We used the average value of 73% for the calculation of the peak curve of the triangular distribution, and the minimum and maximum estimates for the minimum and maximum of the triangular distribution.

European consumption data for PVC rigid and flexible films from 1960–2016 was also compiled by Ciacci et al.[15]. However, no data related to PVC film consumption in individual years in the time period before 1991 was provided by the study. Therefore, we could not do a direct comparison with the data from Kawecki et al.[10] that we utilized with this study. The total PVC consumption amounts in Europe from Ciacci et al.[15] are higher than in Kawecki et al.[10]. This may indicate that the PVC flooring consumption in Switzerland estimated via data from Kawecki et al.[10] is slightly too low. The same appears to be potentially the case when looking at the transition period of data sources and calculation approach from 1987 to 1988, resulting in tendentially higher consumption estimates after 1988 than before.

Model period from 1988 to 2022

Consumption of PVC flooring in Switzerland between 1988 and 2022 was estimated based on PVC flooring production in Switzerland and PVC flooring net import. The reason why we did not use this data for earlier years is that the Harmonized System trade categories, which list PVC floor and wall coverings as an individual category, was only introduced in 1988[16].

Trade amounts of PVC floor and wall or ceiling coverings were available from the trade statistics (Harmonized System tariff heading 3918.10 - Floor coverings, whether or not self-adhesive, in rolls or in the form of tiles, and wall or ceiling coverings "in rolls with a width of >= 45 cm, consisting of a layer of plastics fixed permanently on a backing of any material other than paper, the face side of which is grained, embossed, coloured, design-printed or otherwise decorated", of polymers of vinyl chloride[17]; Figure S2). The share of these trade amounts constituted by flooring was estimated to amount to 67–80% based on PE Europe GmbH et al.[14]. The average value of 73% was used as value for the calculation of the peak of the triangular distribution, and the minimum and maximum estimates for the minimum and maximum of the triangular distribution.

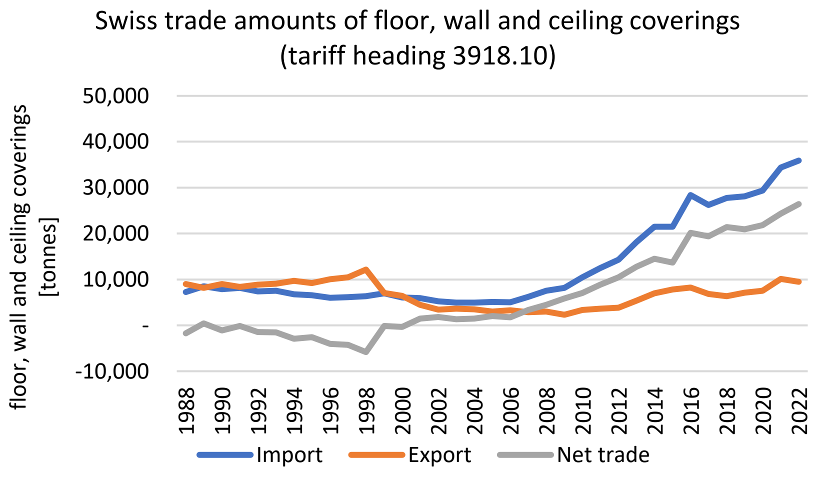

Figure S2: Swiss trade amounts of floor, wall and ceiling coverings (tariff heading 3918.10)[17]. The underlying data are given in the SI2 file.

Swiss PVC flooring production amounts were estimated based on the total PVC conversion in Switzerland, under consideration of additional mass-wise important constituents of PVC flooring such as plasticizers and fillers.

Domestically in Switzerland, PVC was produced until 1995 (Figure S3), with a capacity of 40,000–50,000 tonnes per year[18–20]. This is visible in the gross export amounts dropping strongly from 1995 to 1996 (Figure S3). In the years before 1995, PVC exports increased steadily. Based on this, we assumed that the plant capacity utilization was increased linearly in the years before closure from 80% until 1992 to 110% in 1995, which is a typical behavior. Since 1995, PVC has no longer been produced in Switzerland[11,20]. Export amounts of PVC after 1995 are to the largest extent compounds made with imported PVC and plasticizers or other additives (see export amounts of respective tariff headings in the SI2 file) and to a small extent direct re-exports.

The imported PVC amount was calculated as the sum of the PVC amounts imported under the following tariff headings[17]: 3904.10 - Poly"vinyl chloride", in primary forms, not mixed with any other substances; 3904.21 - Non-plasticised poly"vinyl chloride", in primary forms, mixed with other substances; 3904.22 - Plasticised poly"vinyl chloride", in primary forms, mixed with other substances. Trade amounts of vinyl chloride copolymers (traded under other tariff headings) have been minor.

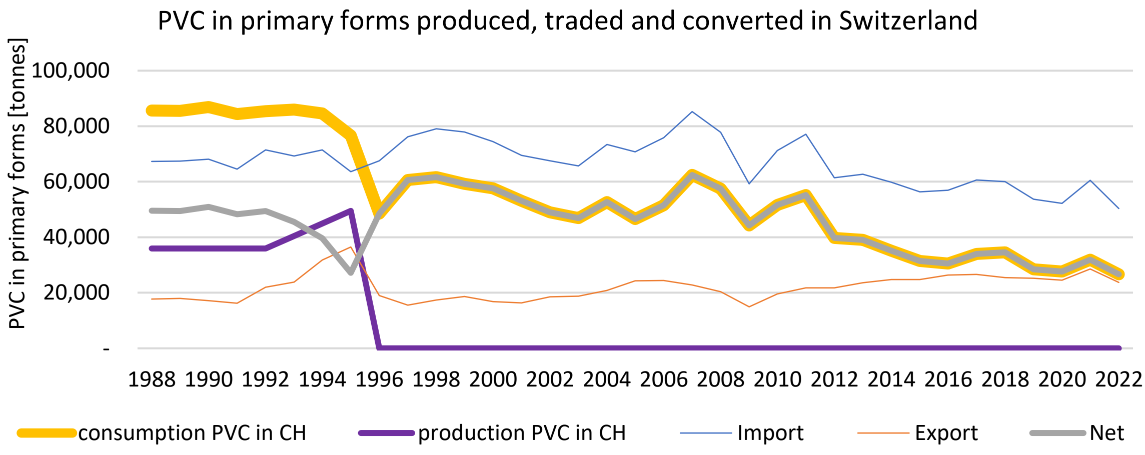

Figure S3: PVC production, trade and consumption in Switzerland. The consumption amount corresponds to the PVC amount converted to products in Switzerland and is the sum of Swiss production and net import. CH: Switzerland.

Of all PVC used for plastic product manufacturing in Switzerland, a share is used for flooring production. Swiss production statistics are not detailed enough so to allow to derive PVC flooring production amounts[21]. Therefore, we had to estimate the share of all PVC converted in Switzerland used for flooring production. For this, we departed from today's situation, knowing that currently around 95% of the PVC floorings used in Switzerland are imported[22]. Knowing the net imported flooring amounts, we derived the amount of PVC flooring (including additives) produced in Switzerland, for the three import values considered for the triangular distribution in the year 2022. Based on the flooring production in Switzerland (using PVC and additives), we derived the share of PVC converted to flooring by assuming a PVC content in

flooring of 30% based on Schiller[23], which is in line with information obtained in personal communication[20]. In a minor part of the total PVC imported, additives are already contained (see import amounts of individual tariff headings in the SI2 file), which we neglected for the sake of convenience, assuming that the complete PVC imports are pure PVC. We then assumed different scenarios for the development of the share of the total PVC converted in Switzerland used for flooring production over time. Namely, we kept the share constant or assumed a linear increase or decrease from the year 1999 to the year 2022 (for which we had estimated the share). In doing so, we considered flooring export amounts (Figure S4), knowing that re-export of floorings is rare[20], meaning that exported flooring amounts are usually produced in Switzerland. Especially, the scenario of an increasing share of PVC converted to flooring over time was considered based on the increasing export amounts. Prior to the year 1999, in 1998, two Swiss PVC flooring production plants shut down[20]. Therefore, we assumed that from 1998 to 1999, the share of PVC converted in Switzerland used for flooring production dropped, so that the difference of PVC flooring production between these two years roughly corresponds to the capacity of the two plants that closed down. This capacity amounted to approximately 20% of the total PVC flooring production capacity in Switzerland[20]. We assumed that the two plants increased capacity utilization in the year before closure, which is in line with the PVC flooring gross export amounts from Switzerland increasing before 1998 and then dropping to 1999 (Figure S2). Specifically, this leads to a drop in PVC flooring production by 1,400-2,900 tonnes from 1998 to 1999 (for the low, intermediate and high estimates). In 1995, PVC production in Switzerland ceased (see above). We assumed that PVC flooring production did not equally decrease in that year, as plants likely try to keep production rather constant. Therefore, we adapted the share of the total PVC converted in Switzerland used for flooring production accordingly, assuming that in 1995 and 1996 roughly the same amounts of PVC flooring were produced in Switzerland. Constant production may have been achieved despite lower total PVC amount consumed in Switzerland because of stored material amounts. We do, however, also consider a high production scenario in which we keep the share of PVC converted to flooring constant despite shut-down of Swiss PVC production. For the time period from 1995 back to 1988, we assumed either a constant or an increasing share of total PVC used for flooring production over time. We did not consider a decreasing share over time, as this would have led to PVC flooring consumption amounts in 1988 much higher than the estimate for 1987, derived via a different calculation approach (as we calculated backwards, i.e. estimated the share for 1988 departing from the share in 1995). The resulting shares of all PVC converted in Switzerland used for flooring production over time are similar to the share of PVC flooring production in Switzerland out of all PVC product manufacturing in Switzerland in 2017 (7%[24] vs 0.9–7.5% in the present study). In this, the value used for comparison refers to PVC including additives, which may affect the relation of produced PVC amounts and produced PVC flooring amounts, as PVC flooring, for instance, contains a large amount of plasticizers, in contrast to other PVC products. Nevertheless, when assuming that only 30% of the PVC material used for flooring is PVC (and the rest are additives), and 100% of the PVC material used for other products is PVC, a share of 2.1% results, which is also within the range of shares used in this study. Also when doing a comparison with the share of PVC used in flooring out of all PVC used in 2017, for a quick estimate assuming that 30% of all flooring is PVC and the remaining products are made of 100% PVC, a similar value as the mentioned shares used in this study results (4%[24]). All values along with further details are available in the SI2 file.

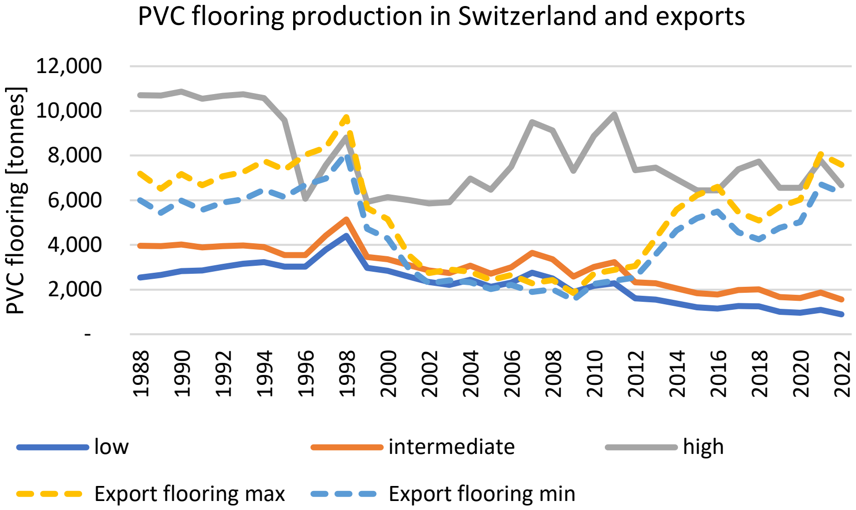

Figure S4: Production of PVC flooring in Switzerland over time (low, intermediate and high estimates) and gross export amounts. The minimum and maximum curves for exported floorings consider shares of 67–80% floorings of floor and wall coverings. The amounts include PVC and additives used in floorings.

The resulting PVC flooring consumption in the time period from 1988 to 2022 overlaps with an estimate based on the European consumption amounts as done for the time period prior to 1988. It overlaps as well with the imported flooring amounts, which are usually not re-exported, but used in Switzerland[20] (Figure S5).

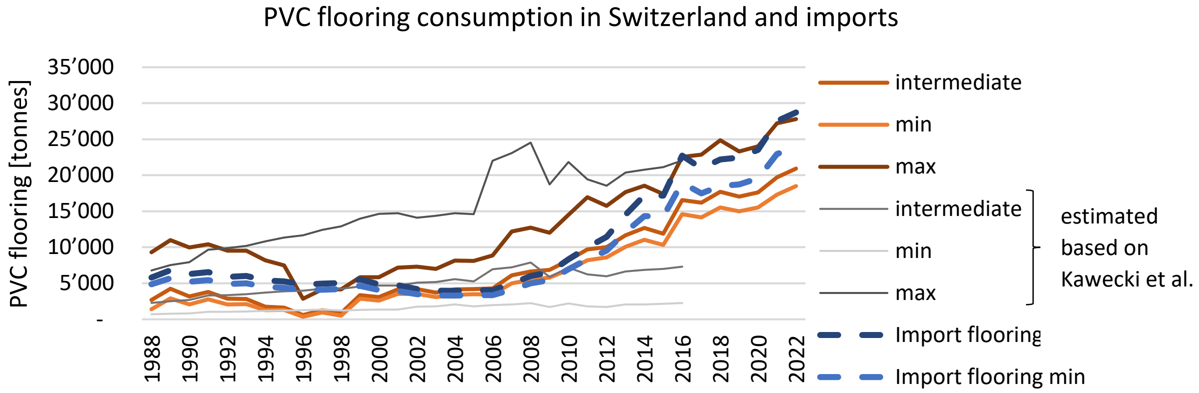

Figure S5: Consumption of PVC flooring in Switzerland (low, intermediate and high estimates) and gross imports. The minimum and maximum curves for imported floorings consider shares of 67 and 80% floorings of floor and wall coverings, respectively. The grey curves represent consumption estimates based on data from Kawecki et al.[10]

Model period from 2023 to 2100

Future consumption (starting from the year 2023) was forecasted based on per-capita consumption amounts and population development. For the latter, scenarios were available until the year 2050[25], which were extrapolated, fitting the past trend with a polynomial curve of second order (Figure S6). Three scenarios were available – a reference scenario along with a high and a low scenario, which lead to a maximum population number around 2070, a

continuous increase of population until 2100, or a maximum population number around 2045, respectively.

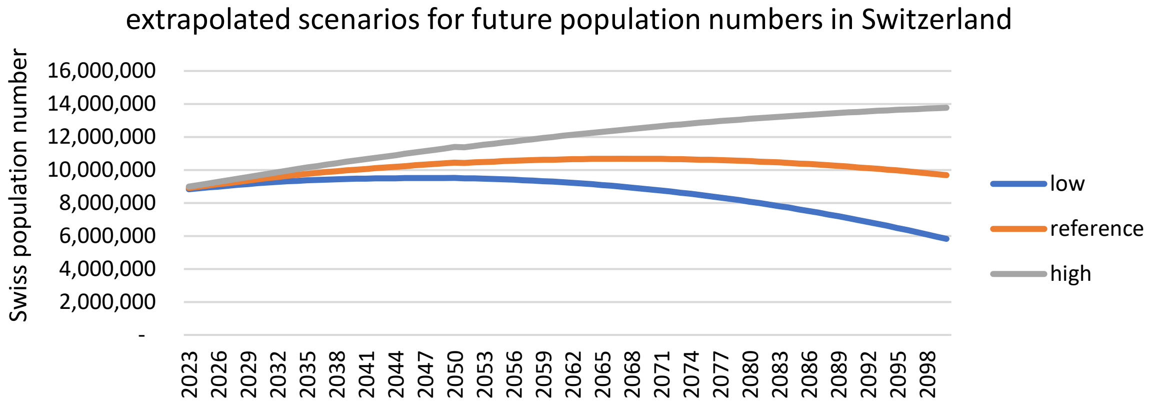

Figure S6: Extrapolated scenarios for future population numbers in Switzerland

Regarding the per-capita consumption of flooring, we departed from the average per-capita consumption within the last five years before 2023 (2018 – 2022). As consumption had been steadily increasing in this time period, our modelling approach leads to a small drop in consumption in 2023. For the development of the per-capita consumption, we considered three cases. In one case, we assume the per-capita consumption remains constant. In the two other cases it either decreases or increases. This was assumed, as there are both indications that may lead to either an increase or a decrease in consumption in the future: on the one hand, a decrease may occur due to efficiency increase, sufficiency or the trend towards reducing fossils consumption (cf. UNEP[26], IPCC[27] or ECHA[28]); on the other hand, an increase may also happen, if the living area per capita increases or more frequent renovation occurs due to increasing wealth, or PVC is cheaper than alternatives and substitutes for floorings made of other materials (cf. UNEP[29], Bakker et al.[30] or Fortune Business Insights[31]). Specifically, for the decreasing per-capita consumption scenario, we assume a per-capita consumption linearly reducing so to halve until 2100. For the increasing per-capita consumption scenario, we assume the per-capita consumption linearly increases until an increase by 50% in 2100. There are arguments supporting a linear development of the per-capita consumption. Certain economic estimates for past and future development of PVC flooring consumption show or assume such a trend (increasing for Germany, considering the comparatively stable population number[32–34]; increasing worldwide, considering linear population growth during the relevant time period[35]). Exponential growth seems implausible, due to several factors limiting the PVC flooring consumption: the flooring area is generally limited, thus, the demand for it is satiable (unlike, for instance, the demand for textiles or packaging, where space considerations are less of a restriction, cf. Wiprächtiger et al.[36], Zink and Geyer[37]). PVC flooring may be exchanged more frequently at increasing wealth, but due to related organizational effort, there seems to be a limit also in this respect. Last, while it is possible that floorings of other materials are substituted by PVC flooring, the overall space limitation still applies. Based on the mentioned considerations, an asymptotic consumption development seems also plausible. We did not consider such a development, as its direction would be between the linear development and the constant consumption scenario that we modelled. Predicting the future trajectory of consumption accurately is difficult, so we aimed

at covering the realistic option space by assessing the range of possible developments derived from different (partially opposing) existing trends. Note that our model illustrates possible substance flow situations that would occur in case of certain developments (what-if scenarios).

S1.3.2 Waste

For calculating the waste amounts resulting from consumption over time, we used a lifetime-based calculation approach[38]. We selected appropriate lifetime distributions based on literature. Commonly, lognormal distributions are used for representing the lifetime of products in material flow analyzes[39,40]. Besides, also the use of Weibull distributions has been suggested[39,41–44], which have been shown to provide a good fit to different types of lifetime data[45,46]. We, therefore, considered lognormal distributions with the probability density function

$$f_Y(y) = \begin{cases} 0 & y \le 0 \\ \frac{1}{\sqrt{2\pi}\sigma y} \exp\left\{-\frac{1}{2}\left(\frac{\log(y)-\mu}{\sigma}\right)^2\right\} & y > 0, \end{cases}$$

and the mean

$$\mathbb{E}[Y] = e^{\mu+\sigma^2/2} \ ,$$

as well as a Weibull distribution with the probability density function

$$f(x;\lambda,k) = \begin{cases} \frac{k}{\lambda}\left(\frac{x}{\lambda}\right)^{k-1} e^{-(x/\lambda)^k}, & x \ge 0, \\ 0, & x < 0, \end{cases}$$

and the mean

$$\mathrm{E}(X) = \lambda\Gamma\left(1+\frac{1}{k}\right) .$$

For setting the parameters of the distributions, we first estimated the mean lifetime of PVC flooring. Depending on the application, flooring may be used for 7 to 60 years before being exchanged[22]. For instance, in shops in Switzerland it is common for flooring to be exchanged every 7 years independent of its condition, while in hospitals flooring may last for 20-40 years and in schools even for 40-60 years based on information from a Swiss professional seller of floorings[22]. It may also happen that old flooring is not replaced, but remains under new flooring. As the mean lifetime of PVC flooring, scientific studies have used 5–15 years (flooring as part of this lifetime category[47]), 13.8 years (for building materials[41]), 15 years (based on educated guess[42]) or 20 years[48]. Reports have assumed lifetimes of 10–20 years for most floorings[49] or 2–40 years[14]. Data was also available from tenant protection organizations (e.g. expected lifetime of 20 years[50]) or guarantee documents (e.g. guarantee for 10 years[51]). Based on the information mentioned, we assumed mean lifetimes of 15, 20 or 30 years (Table S1).

We chose the distribution parameters related to the variance (k for Weibull and sigma for lognormal) so that the same relative standard deviation of 80–81% (in relation to the mean) resulted for all distributions. This value resulted from assuming a failure rate increase over time for the Weibull distribution, meaning that the k value of the probability density function is larger than one (assumed 1.25; Table S1). Anyways, changes in the standard deviation of the lifetime were shown to potentially only have a low influence on the results of dynamic flow models[39]. The second parameter of the distributions (lambda for the Weibull distribution, mu for the lognormal distributions) was then calculated so that the distribution mean corresponded to the estimated mean lifetimes.

Table S1: Lifetime distributions assumed for PVC flooring in Switzerland (independent of consumption year)

**distribu-tion shape**	**mean**	**σ / k ***	**μ / λ ****	**scale *****	**standard deviation**	**standard deviation relative to mean**
lognormal	20	0.7	2.8	15.7	16	80%
lognormal	15	0.7	2.5	11.7	12	80%
lognormal	30	0.7	3.2	23.5	24	80%
Weibull	20	1.25	21.5	21.5	16.1	81%

* σ for lognormal, k for Weibull
** *μ* for lognormal, λ for Weibull
*** scale is the input parameter used in the scipy Python package

The resulting probability density functions are shown in Figure S7.

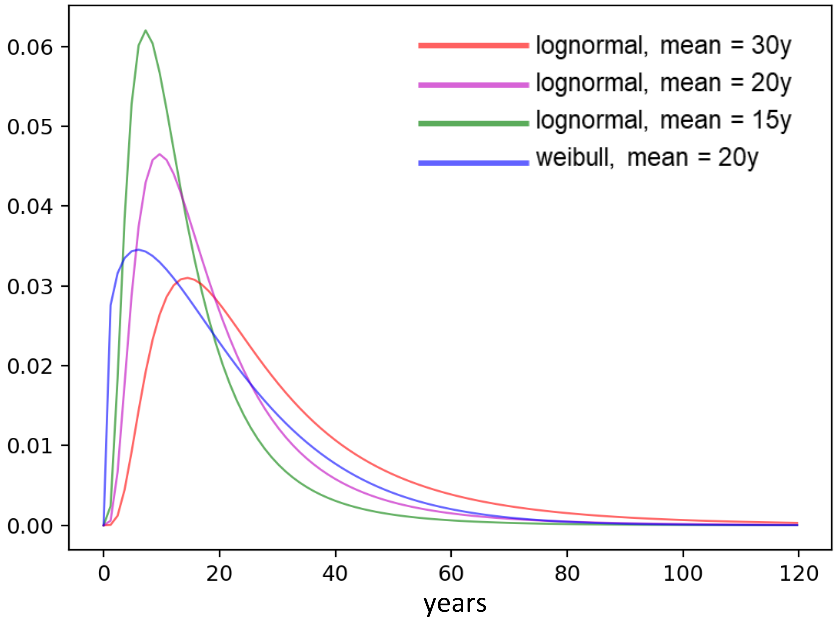

Figure S7: Probability density functions of the lifetime distributions considered for PVC flooring

S1.3.3 Recycling rate

For the calculation of recycled waste amounts, we used curves of recycling rates (RR; see main paper for definition) over time. These curves were based on estimated recycling rate values for the following points in time, between which linear transitions were assumed. We assumed no recycled material was utilized in PVC flooring consumed in Switzerland before 1996, when the collection system for Swiss PVC flooring waste was established[52]. This is in line with the European PVC flooring recycling quantities ramping up from very small amounts around the year 2000, when the organization today responsible for recycling Vinyl Plus was founded[53,54]. By assuming that globally, PVC recycling was not more advanced than in Europe, it was estimated that before 1996, no considerable amount of secondary PVC was used in the Swiss flooring.

The next point used to define the recycling rate curve was the year 2017. For this year, estimates for collected amounts of Swiss PVC flooring waste were available, amounting to 625 t[24] and about 100 t[55]. We assumed that a material amount amounting to the average of these two values was collected and determined the recycled amount considering a recycling efficiency of 87%[56]. We then calculated the recycling rate based on the waste amount in 2017 calculated with the model (average for the four different lifetime distributions; 5400 t), resulting in 5.8%. To account for uncertainties, both values half and double as high were considered, covering a range of realistic recycling rate values based on the Swiss PVC flooring collection organization[22], as well as an extreme value three times as high as the best-estimate. As a comparison, we estimated the recycling rate in Europe in 2022 (1,772 t of post-consumer PVC flooring recycled by Revinylfloor within the Vinyl Plus framework[54]; 410'926 t of PVC flooring waste in Europe based on the Swiss waste amount from the model and the population numbers for Europe[57] and Switzerland[12], resulting in a very low recycling rate of 0.4%).

The curves were extrapolated with the same inclination until the year 2024, resulting in a RR value of 7.7% in 2024 for the best-estimate curve. In the year 2025, based on the Vinyl Plus 2030 Commitment, 900,000 tonnes of recycled PVC shall be used in new products[54]. If all PVC products were equally recycled, with two thirds of all PVC being used in building and construction and 14% thereof being used in flooring, about 10% of all recycled material would correspond to flooring in case of similar lifetimes of all PVC products. This would correspond to an amount of 90,000 tonnes (including secondary material from pre-consumer waste). With waste amounts in 2025 from the model scaled to Europe via the relation of population numbers, this would correspond to a recycling rate of 18%, which is similar to the value in 2025 for the curve depicting a high past and high future recycling rate in the model (Figure S8). Another target, by the flooring producer Gerflor France, is to recycle 55,000 tonnes of PVC flooring pre- and post-consumer waste per year closed-loop from 2025 onwards[58]. If this refers to the recycling input and the recycling efficiency is 87%[56], this would correspond to a recycling rate of 65% when referring to French waste (estimated via Swiss waste amounts scaled with population relation). However, the waste amounts used as a reference should possibly be higher, as Gerflor may recycle waste from other countries than France as well. Besides, the difference to our model is that the latter only considers post-consumer waste, and not pre-consumer waste, which tends to be much easier to recycle.

For the year 2040, we assumed a maximum recycling rate achievable of 46% based on Klotz et al.[59]. We assumed that in the long-term future, potentially the recycling rate may be further increased due to technological developments, up to 70% in 2100. This value seems

theoretically possible to achieve, as 90% of PVC flooring in buildings could be stripped[22] and separately collected. Stripping of flooring may, however, be effortful in older flats, in contrast to industrial buildings, where flooring removal can be done by machine[60]. Therefore, economic feasibility and environmental efficiency of a separate collection need to be considered. High future recycling rates also seem to be compatible with the maximum secondary material shares utilizable in flooring. The maximum recycled content resulting for the future scenario involving high recycling (67 weight% in the year 2100; Figure S24) is in line with the estimated possible recycled PVC share in flooring (maximum 63 weight%[61]) and is also already achieved in individual floorings (69 weight%[5]). The recycled content needing to be achieved across all floorings, though, lies at the upper boundary of feasibility estimates. Due to the mentioned and potential other constraints, besides the maximum recycling scenario, a lower future recycling rate scenario was also considered with recycling rates only half as high as in the maximum scenario. Besides, a scenario of a constant future recycling rate of 5%, similar to its current best-estimate value, was considered. Notably, an EU project investigating how to increase the recycling of PVC flooring considers as little as 8% of all flooring waste to be collectable[62], which would only allow to achieve the very low future recycling rate scenario.

The recycling rate curves resulting from the modelling approach described are shown in Figure S8, with the underlying data (values for individual years) given in the SI2 file. All waste arising in a year (independent of its age cohort) was assumed to be recycled to an equal degree (defined by the recycling rate in that year).

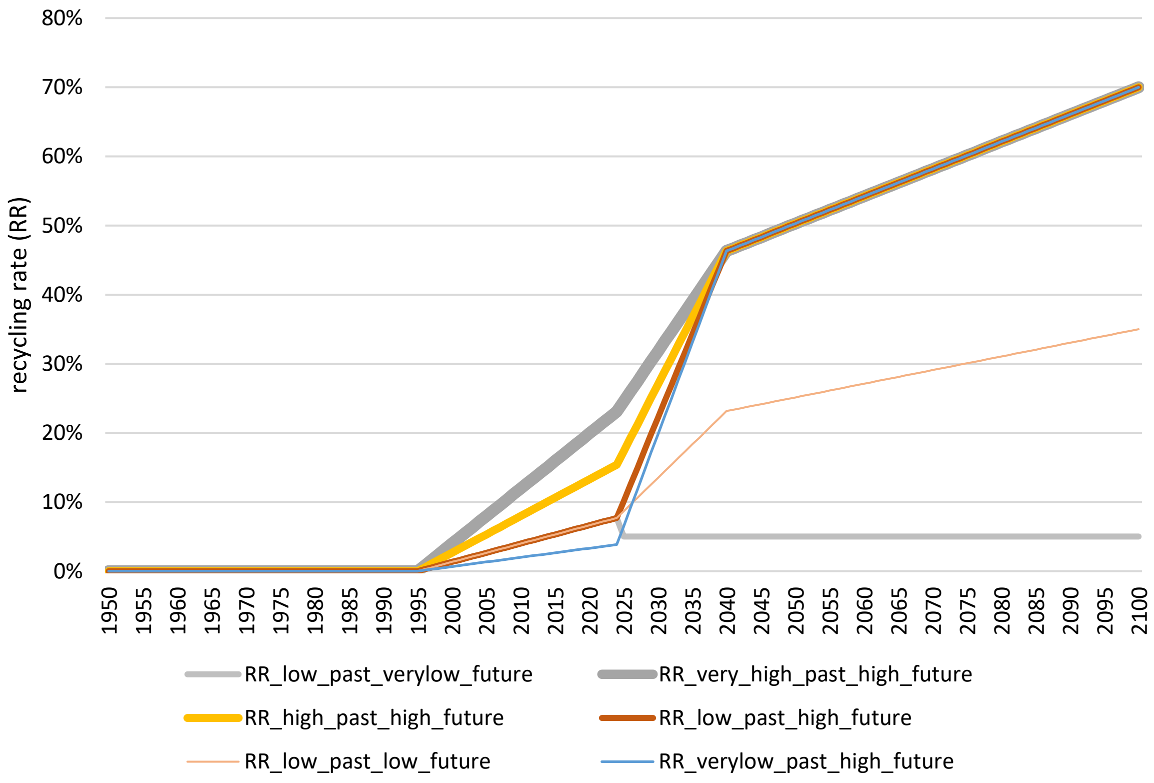

Figure S8: Recycling rate curves considered in the MFA-SFA model. RR: recycling rate.

The geographically closed-loop modelling approach for recycling, as described in Section 2.2 of the main paper, seems suitable for different reasons. Secondary material from Europe likely

has a similar composition to the secondary material from Swiss waste, because there are reasons for assuming that the PVC flooring markets in the two regions are similar. For instance, similar product safety regulations apply[63,64]. In addition, the recycling of Swiss PVC flooring waste takes place in a German plant[24], i.e., the recycling technology applied is the same as for German waste. Possibly, PVC flooring recycling technologies are harmonized on a European scale, as a European recycling organization[54] exists. Besides, a large share of PVC flooring consumed in Switzerland stems from China, via which secondary material may be introduced to Switzerland. In China, however, strong economic growth happened later than in Europe, meaning that PVC waste amounts today are relatively lower and potentially also recycling rates. This may lead to the conclusion that most recycled PVC used in Switzerland stems from Europe (and, therefore, has a similar composition as secondary material from Swiss waste).

Regarding the closed-loop modelling on a product level, while secondary PVC stemming from other applications than flooring is utilized in flooring, these applications seem to be limited[24,53]. One product which is recycled into flooring are cables[53], which in Switzerland are not allowed for recycling[65], so the use of secondary material from cables in flooring may not be of high relevance for Switzerland. If the share of secondary material utilized in flooring stemming from other than flooring waste is indeed limited, and the concentrations of plasticizers in this secondary material from other products are not very different from the flooring secondary material (which may be the case, as possibly waste from other plasticized products is utilized), the closed-loop modelling approach is deemed to represent the actual situation well. In turn, recycled PVC from floorings is, besides closed-loop recycling, also used in other construction products[24,66,67]. This can have the consequence that recycled material shares in flooring are lower (if not made up for by recycled PVC from other products used in flooring), however, it can lead to legacy plasticizer distribution to other products. With our model, we show the case of all secondary material produced from flooring being re-utilized in flooring.

S1.4 Substance flow analysis (SFA)

The composition of virgin PVC flooring over time was estimated as described in the following. We assumed the composition of virgin material used in flooring manufacturing to be independent from the usage of recycled material. This assumes that only for the virgin PVC amount plasticizers are added, which seems plausible, as recycled PVC is likely known by the manufacturers to already contain plasticizers. For the scenario of solvent-based removal of plasticizers, alternative plasticizers would need to be added to the secondary PVC. If DEHT was used as such an alternative plasticizer, DEHT consumption would be to some extent higher than in the model (this was not considered). If other available alternatives (see Section 1 of the main paper) were used to substitute for phthalates removed during recycling, this would not affect the substance flows from this model.

S1.4.1 Market shares

A high share of PVC flooring is plasticized based on the measurements by Wiesinger et al.[68], who found plasticizers in about 90% of all flooring on the market, with methods being able to detect most common plasticizers. Standards were used for *ortho*-phthalates, *tere*-phthalates, trimellitates, benzoates, cyclohexanoates and aliphatics. Chlorinated paraffines may not have

been detected. We, therefore, assumed that half of the samples in which no plasticizers were found did indeed not contain any plasticizers, while the other half contained a plasticizer that was not detected, i.e. we assumed a share of 95% of flooring being plasticized. Besides, the samples of Wiesinger et al.[68] were mostly flexible based on own judgement, and flexible PVC usually contains plasticizers[69], while even semi-rigid PVC may contain plasticizers[70].

Of all plasticizers used, a share are *ortho*-phthalates – to which DEHP and DiNP belong – and another share are *tere*-phthalates – to which DEHT belongs. We conducted one estimate for joint timelines of the market shares of *ortho*-phthalates and *tere*-phthalates (Figure S9). This estimate was mainly based on data for plasticizer utilization on the European market (across different products, whereby 85% of plasticizers are employed in flexible PVC applications)[71], assuming that the Swiss is similar to the European market. We only regarded plasticizers relevant for flooring (including high molecular weight *ortho*-phthalates, *tere*-phthalates and other plasticizers among which trimellitates, benzoates, cyclohexanoates, aliphatics and others, but not low molecular weight *ortho*-phthalates). Based on this data, in 2005, 81% of all plasticizers were (high molecular weight) *ortho*-phthalates, while in 2020 their market share was only 55%. We assumed a linear decrease between these two data points. For the time period before 2005, we extrapolated the linear trend backwards until an *ortho*-phthalates market share of 90%, which is reached in the year 2000. This market share corresponds to the current worldwide market share of *ortho*-phthalates (about 87%[23]), which we assumed applied to Europe and Switzerland as well before consumption reduction of *ortho*-phthalates related to hazardousness (Section 1 of the main paper). We assume that before the year 2000, the market share was constant at 90%. After 2020, we assume a further decrease of *ortho*-phthalate use[72], assuming a continuation of the linear trend until a market share of 20% is reached in 2040. We assume that this market share then remains constant until the year 2100, while phase-out scenarios for DiNP are considered (see below). With this modelling approach, from 2040 onwards, about half of the plasticizer market is constituted by *ortho*- and *tere*-phthalates (see below).

The *tere*-phthalates market share amounted to 0% in 2005 and 14% in 2020[71]. We assumed that before 2005, no *tere*-phthalates were used. Between 2005 and 2020, we assumed a linear market share increase, which we extrapolated till 2040, i.e. the *tere*-phthalates share increases as long as the *ortho*-phthalates share decreases. The resulting *tere*-phthalates market share in 2040 amounts to one third, meaning that the ratio of *tere*-phthalates to plasticizers other than *ortho*- and *tere*-phthalates (trimellitates, benzoates, cyclohexanoates, aliphatics and others; 50%) is approximately the same as in 2020[71].

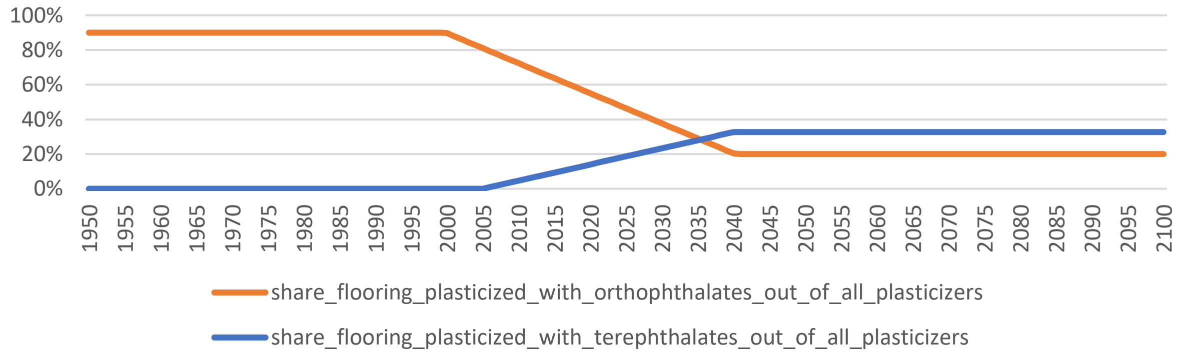

Figure S9: Market shares of ortho*-phthalates and* tere*-phthalates (out of all plasticizers) over time*

We assumed shares of DEHP and DiNP of *ortho*-phthalates that change over time only due to phase-out. Before start of phase-out, we assumed DEHP shares of all *ortho*-phthalates of 90% as the best estimate, as well as 75%, 50% in different scenarios, knowing the DEHP was the dominant *ortho*-phthalate[23,73]. For DiNP, which was the second most important *ortho*-phthalate[23,72,74], we assumed shares of 3%, 10% and 20%, respectively, in the different DEHP scenarios. These shares correspond to one third or slightly more of all non-DEHP *ortho*-phthalates. In addition, we assumed that one third of all DEHP was substituted by DiNP when phased-out, specifically considering that one third of the reduction in DEHP share of *ortho*-phthalates was made up for by DiNP (in addition to the DiNP share of *ortho*-phthalates mentioned). We also modelled scenarios of future phase-out of DiNP (described below).

A consumption reduction and eventually phase-out of DEHP was considered in line with scientific and legislative developments. Specifically, the phase-out timelines of DEHP (Figure S10; values are provided in the SI2 file) were based on events that likely affected DEHP utilization. These events are:

- 1968: first publication pointing to a potential hazard of DEHP[75];
- 2008: SVHC list inclusion[76];
- 2011: authorization list inclusion[77];
- 2015: authorization sunset[78].

The different phase-out scenarios assume measures of different severity taken as a consequence of the events mentioned, leading to a reduction to different extents during the individual time periods considered (Figure S10).

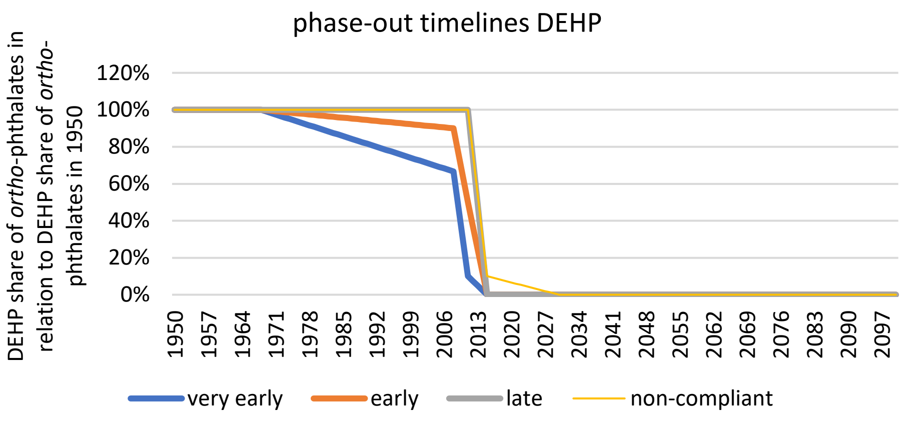

Figure S10: Phase-out timelines of DEHP considered. The percentages refer to the reduction of the DEHP share of all ortho-*phthalates, with the DEHP share of all* ortho-*phthalates in 1950 as a reference.*

For DiNP phase-out, we considered an early and a late scenario (Figure S11). The early scenario was based on the timeline of DEHP restriction, which took approximately 10 years (from SVHC list inclusion in 2008 to sunset date in 2015) and assumes that due to preparation needed, a reduction of the DiNP share of *ortho*-phthalates may start in 2030 earliest. In the late phase-out scenario, phase-out starts only ten years later in 2040 and takes 10 years as well.

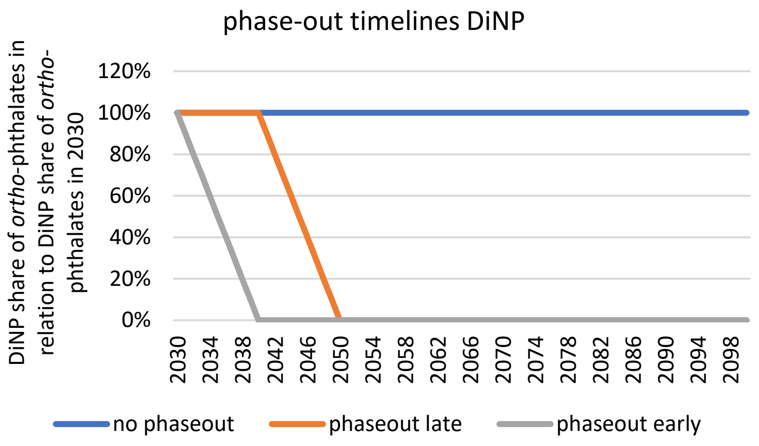

Figure S11: Phase-out timelines of DiNP considered. The percentages refer to the reduction of the DiNP share of all ortho-*phthalates, with the DiNP share of all* ortho-*phthalates in 2030 as a reference.*

A possibility that we did not consider was a temporary increase in consumption before the sunset date due to a potential depletion of stocks. We do, however, not assume that such a potential temporary increase would have been of such a magnitude that it would have affected future concentrations considerably.

We assumed a constant share of DEHT of all *tere*-phthalates, as there are currently no reasons justifying a phase-out[28]. DEHT is the main *tere*-phthalate[68,72]. Therefore, we considered a high value of 90% of all *tere*-phthalates being constituted by DEHT (Figure 1 of the main paper) as well as a lower value of 75% of all *tere*-phthalates being constituted by DEHT (Figure S28).

S1.4.2 Concentration

We estimated the concentration of plasticizers in flooring based on typical formulations of PVC flooring. In doing so, we considered varying shares of plasticizers as well as fillers, which are the two constituents of PVC flooring that besides the PVC resin constitute considerable shares of the total mass[23]. For 100 parts of PVC resin, we considered 10–100 parts of plasticizer based on data related to DEHP in flooring (10–30 parts and 88 parts in flooring for wiring[70]), plasticizers in PVC flooring (40–70 parts[23]) and main plasticizers in PVC (5–180 parts[70]). We varied the filler content from 0[23] to 300 parts[23]. We considered the lowest plasticizer share along with highest filler share as minimum plasticizer mass share in PVC flooring (2%) and the highest plasticizer share along with lowest filler share as highest plasticizer concentration (49%). Based on this, we modeled the concentration in individual floorings as a normal distribution with the mean lying in the middle of the concentration range (26%) and chose the standard deviation so that 99.73% of floorings lie within the minimum and maximum concentration estimate (i.e. corresponding to one third of the difference between mean and minimum/maximum). Based on the concentration distribution in individual floorings, we estimate the distribution of the average concentration of plasticizers in floorings on the Swiss market, used as a model input. Assuming that the concentration distribution is the same for all floorings on the market, the central limit theorem can be applied[79]. According to this theorem, the average concentration is (independently from the distribution of the concentration in

individual samples with equal concentration distribution) normally distributed with the same mean as for the concentration in individual floorings and a standard deviation amounting to the standard deviation of the individual samples divided by the number of floorings on the market. For the latter, we used a number of 100 based on a rough estimate of 10 sellers with each 10 different products. The resulting standard deviation of the average concentration, due to the high number of floorings on the market, amounts to only 0.08%. Note that in the described calculations, the mean concentration in the samples was assumed to correspond to the value lying in the middle of the determined concentration range. This value of the mean is uncertain, while the central limit theorem gives the distribution of the average concentration of a number of samples with known mean. Therefore, a larger uncertainty than derived via the central limit theorem as described may have been more appropriate for use in the SFA.

We assume that DiNP is used in similar concentrations as DEHP due to similar properties[73]. This is in line with substitution factors for DiNP utilization instead of DEHP close to 1:1 mentioned (1.06[73]). Also the densities of DEHP (0.986[73]) and DiNP (0.972[73]) are similar.

DEHT, as well, has a similar density as DEHP (0.984[73]) and can substitute for DEHP in a ratio close to 1:1 (1.03[73]). It is also mentioned to be used in similar mass shares (15-30%[80]). Therefore, we assumed the same distribution of the average concentration as for DEHP for DEHT as well.

It may be that the plasticizer concentration in PVC flooring has decreased over time. This is indicated by the concentrations used in the German model (Section S1.5.2), which are based on data from Environmental Product Declarations regarding current floorings, being to some extent lower than the concentration estimated for the Swiss model based on plasticizer handbooks. The Swiss estimate is also very similar to concentrations mentioned in an older source[53]. One development that may have led to such a decrease in concentration is the increased use of luxury vinyl tiles[6], which often contain a non-PVC layer[20] and, thus, a lower share of phthalates in relation to the total mass. Such a potential change in concentration over time was not considered in the model.

S1.4.3 Plasticizer losses during use and recycling

We assumed that a share of plasticizers is lost over the use phase as well as in the course of the recycling process. We estimated the losses during the use phase to 2% based on Fantke et al.[81]. For the sake of convenience, we assumed that the loss occurs immediately when a flooring enters the use phase, as losses are small enough to not considerably affect the plasticizer amounts in flooring. The recycling losses were approximated with the losses during the use phase, because of a shorter process duration, however, combined with a higher temperature. For DiNP and DEHT, we assumed the same loss value, based on their similar properties to DEHP[73].

S1.4.4 Contaminant removal

Sort-out of contaminated waste

One way for phthalate removal from recycling streams is sorting out contaminated waste, identified via Fourier-transform infrared spectroscopy (FTIR) measurements.

Other identification techniques such as gas chromatography−mass spectrometry (GC−MS), GC with flame ionization detector (GC−FID) or electron capture detector (GC−ECD) involve time-consuming sample pre-treatment[68,82]. The latter may be avoided by using, for instance, direct analysis in real time (DART)[83]. However, DART−GC−MS is expensive compared to FTIR measurments[84]. Meanwhile, further suitable analytical methods may exist for identifying phthalates.

Determination of phthalate removal efficiency achievable with sort-out

For calculating the maximum share of phthalates that can be removed via sorting out waste containing phthalates, identified with FTIR measurements, we assumed that all waste is tested for phthalate-content with FTIR, and all waste identified via FTIR as phthalate-containing is removed from recycling. What remains in recycling streams are phthalates contained in waste in concentrations below the limit of detection (LOD) of FTIR (0.1 weight%[68]), as well as waste with phthalate concentrations above the LOD, which is not identified as such due to the limited measurement sensitivity (97.2%[68]; false negatives). These remaining phthalate amounts were calculated, assuming that the phthalate concentrations in samples below the LOD amount to half of the LOD. Putting the remaining phthalate amount in relation to the phthalate amount present in waste before sort-out of contaminated waste, the recycling transfer coefficient (TC) for phthalates was calculated.

The resulting removal efficiency varies over time, as it depends on the concentration and distribution of phthalates in flooring waste. Specifically, at a decreasing share of waste containing decreasing shares of phthalates, the removal efficiency decreases: When phthalate concentrations are high in a large share of flooring waste – a situation that will prevail in the near future due to high DEHP and DiNP use amounts in the past – achievable removal efficiencies of around 97% (close to the FTIR sensitivity) result. With decreasing phthalate amounts in waste (lower concentrations in fewer waste streams), however, as it will be the case for the more distant future, the removal efficiency decreases. This is because a higher share of phthalates in waste remain undetected due to concentrations below the LOD, and the undetected phthalate amounts become more relevant in relation to the total phthalate amount originally present in the waste. However, the resulting increase in transfer coefficient of phthalates from waste to new products is less relevant due to the overall lower phthalate amounts present.

Therefore, the variation of TC was considered in a simplified manner: The highest removal efficiency achievable with FTIR measurements and removal of all identified waste samples containing phthalates amounts to approximately 97% (TC of 3%). We modelled a sort-out scenario with a constant TC of 3% applying from 2025 onwards, achieving the best-case removal, i.e. the lowest concentrations of phthalates in future waste. We use these best-case future phthalate concentrations for calculating TCs that we then utilize as model input. As the scenario considered (constant TC of 3%) involves the lowest phthalate concentrations achievable, the resulting TCs are the highest (TCs are higher for lower phthalate

concentrations in waste as described in the previous paragraph), i.e. we model a worst-case scenario regarding phthalate removal achievable with FTIR. For the scenario with constant TC of 3%, we determined the total phthalate concentration in waste (Figure S12), as FTIR measures the total phthalates amount, not DEHP and DiNP individually. The total phthalate concentration is the sum of the concentrations of DEHP and DiNP, which were considered in the model (Figure S12). The total phthalate amount in waste can be distributed to individual waste streams in different ways, depending on phthalate market shares and recycling practices. We considered two rather extreme cases of 90% or 10% of all waste containing phthalates, respectively, and calculated the corresponding phthalate concentrations in those waste streams that contain phthalates over time (Figure S13), based on the overall concentration of phthalates in waste (Figure S12). Then, we calculated the TCs resulting for the two scenarios considered (phthalates in high share of waste in lower concentrations and phthalates in low share of waste in higher concentrations) over time. As a model input, we divided the time period from today till 2100 in half and considered a constant TC for each time period. Specifically, we assumed a constant TC for the time period until 2060 of 4% for both DEHP and DiNP, corresponding to the average of the TCs calculated for the years 2020, 2030, 2040, 2050 and 2060 for waste shares of 90% and 10% containing either DEHP or DiNP. In the same manner, we assumed a constant TC for the time period from 2061 to 2100 of 17% for both DEHP and DiNP, calculated based on the TCs for the years 2070, 2080, 2090 and 2100. Note that it may happen that more DiNP than DEHP is removed or vice versa, depending on the distribution of these phthalates in removed waste, therefore, the TCs for DEHP and DiNP may still be different in reality.

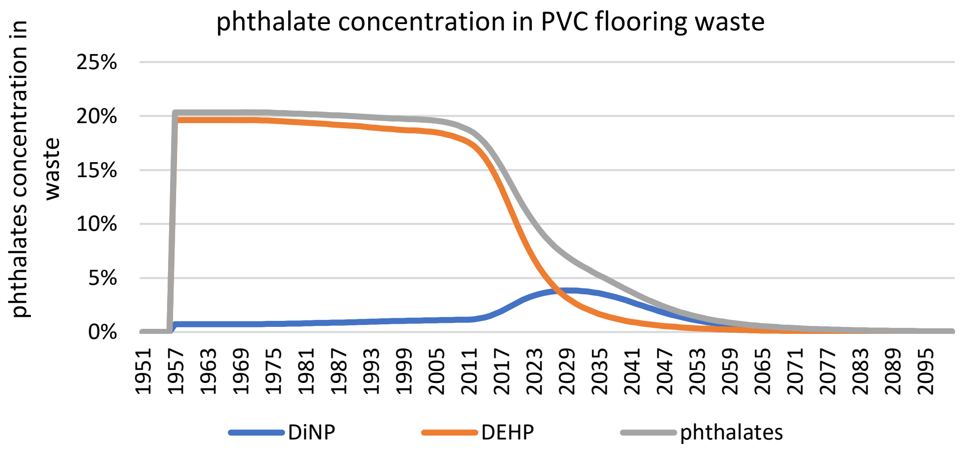

Figure S12: Concentration of DEHP and DiNP, as well as total phthalates (sum of DEHP and DiNP) in PVC flooring waste over time, in a scenario of removing phthalate-containing waste from recycling streams with high efficiency, considering a constant transfer coefficient of 3%, and early phase-out of DiNP

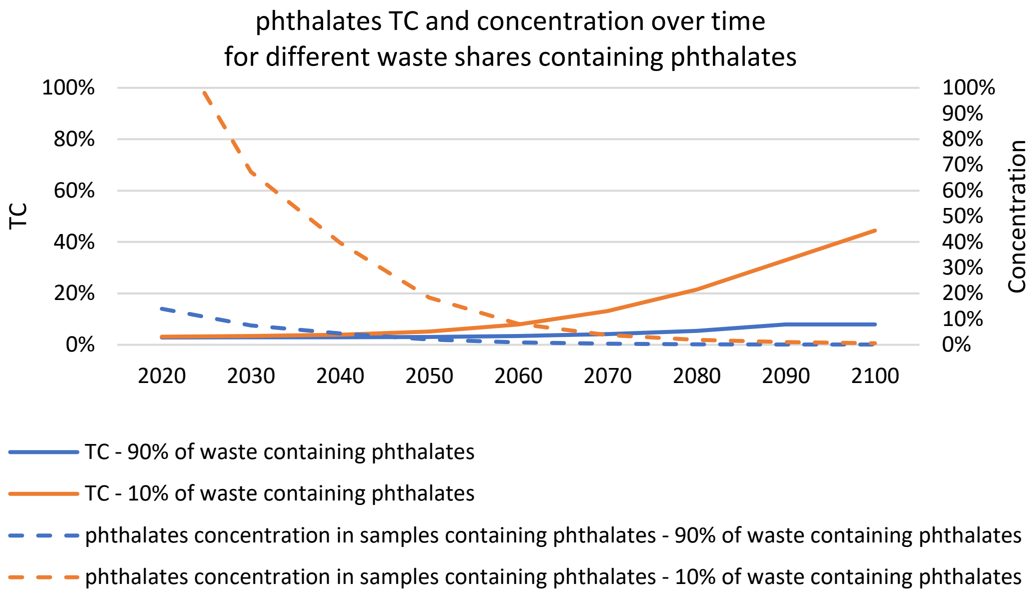

Figure S13: Concentration of phthalates in phthalate-containing waste share, for hypothetical cases of waste shares of 10% and 90% containing phthalates, and resulting transfer coefficients for phthalate removal using FTIR measurements for identification. TC: transfer coefficient.

The TC that would result if only DEHP or DiNP were contained in waste are visible in Figure S14 for DEHP and Figure S15 for DiNP. In this case, the TCs for DEHP and DiNP would amount to 6% and 4%, respectively, for the time period until 2060 and 33% and 21% for the time period after 2060. The lower TCs resulting for DiNP are because DiNP is contained in higher concentrations in waste in the future because of higher use amounts in more recent year. Anyways, the same TCs were applied to both substances as described above, as FTIR measures the total *ortho*-phthalate amount.

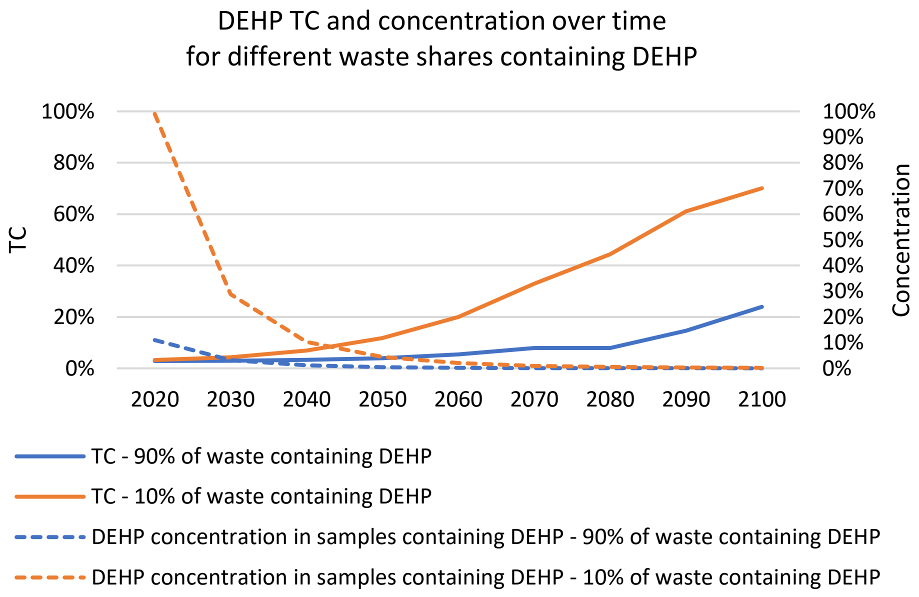

Figure S14: Concentration of DEHP in DEHP-containing waste share, for hypothetical cases of waste shares of 10% and 90% containing DEHP, and resulting transfer coefficients for DEHP removal using FTIR measurements for identification. TC: transfer coefficient.

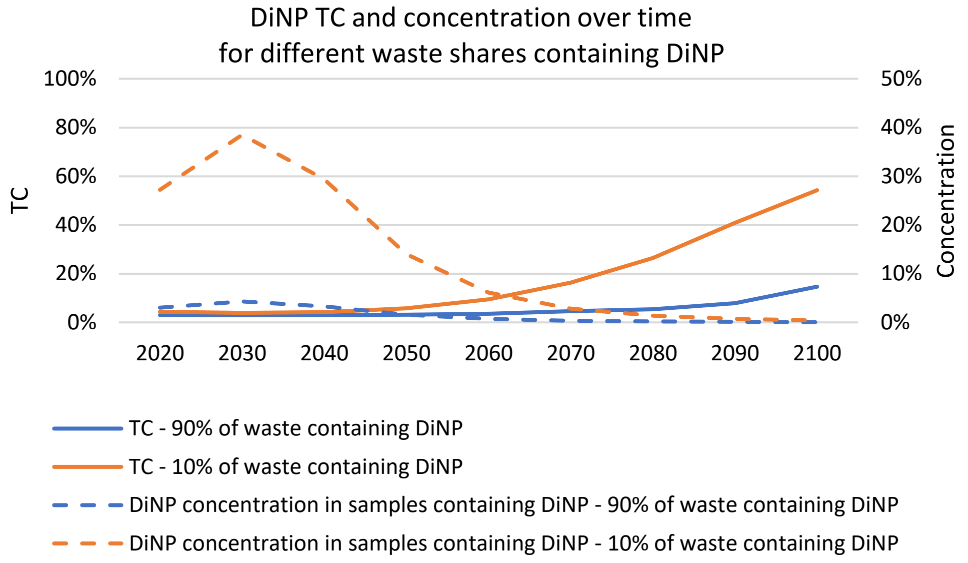

Figure S15: Concentration of DiNP in DiNP-containing waste share, for hypothetical cases of waste shares of 10% and 90% containing DiNP, and resulting transfer coefficients for DiNP removal using FTIR measurements for identification. TC: transfer coefficient.

To give further insights, we also plotted the TCs for the hypothetical cases of a linearly decreasing share of waste containing phthalates along with decreasing or different constant phthalate concentrations (Figure S16, Figure S17 and Figure S18).

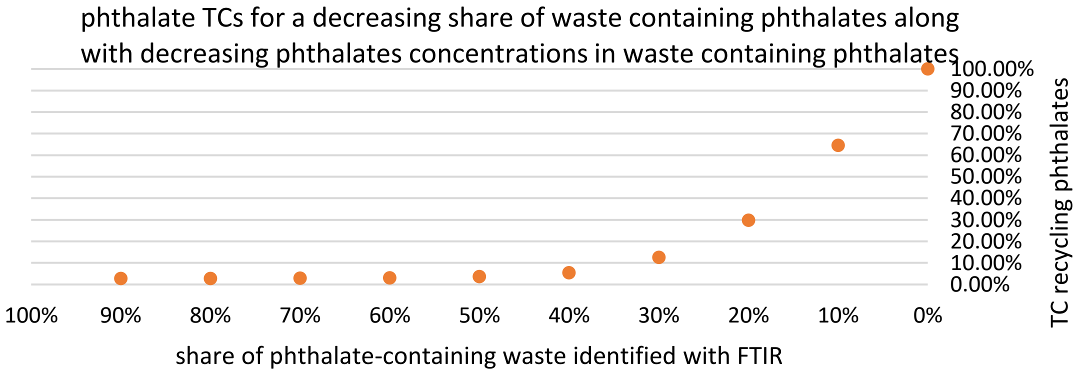

Figure S16: Phthalate recycling TCs for a hypothetical case of a decreasing share of waste containing phthalates along with decreasing phthalates concentrations in waste containing phthalates

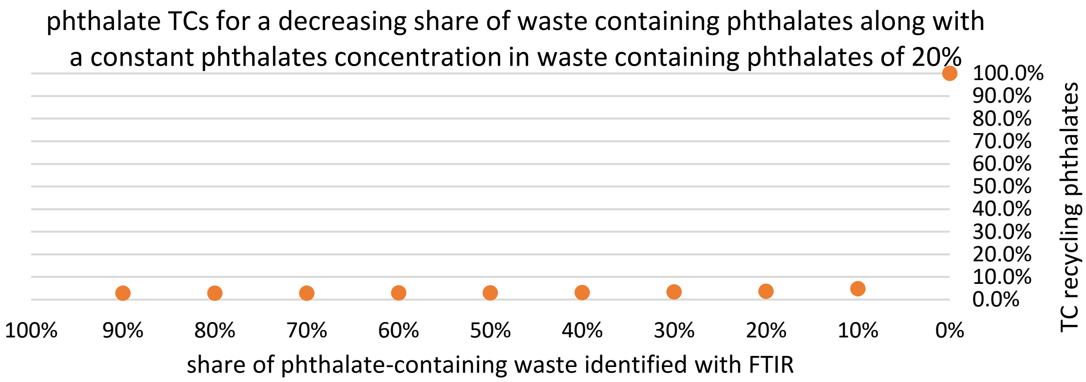

Figure S17: Phthalate recycling TCs for a hypothetical case of a decreasing share of waste containing phthalates along with a constant phthalates concentration in waste containing phthalates of 20%

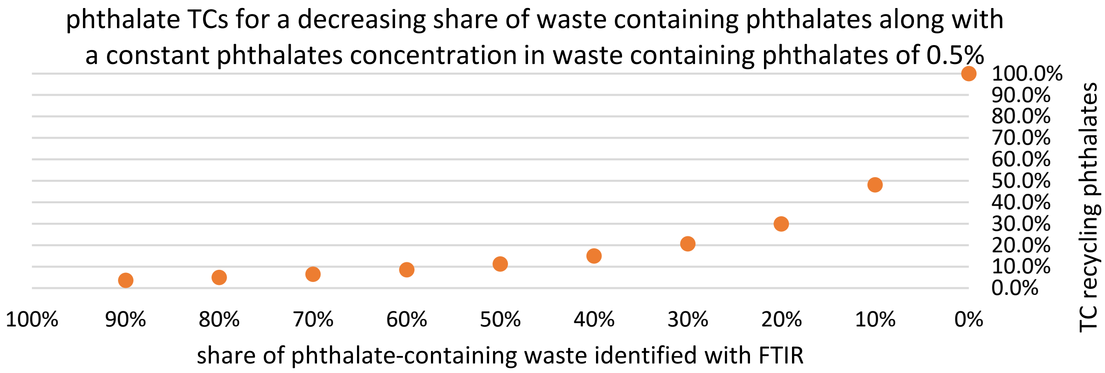

Figure S18: Phthalate recycling TCs for a hypothetical case of a decreasing share of waste containing phthalates along with a constant phthalates concentration in waste containing phthalates of 0.5%

For DiNP, sort-out seems only sensible in case a phase-out is implemented. If no phase-out occurs, DiNP concentrations remain considerable despite sort-out (Figure S27). This situation will occur in case sort-out using FTIR is implemented for the sake of removing DEHP, as then DiNP will be removed as well, because FTIR measures all phthalates. For investigating DiNP sort-out along with phase-out, we consider early phase-out of DiNP (Section S1.4.5; Figure S27). In this case, sort-out still starts before the reduction of the DiNP market share due to phase-out, as we assume sort-out can be implemented faster. This seems sensible to do in case it is confirmed that DiNP poses hazards, for reducing DiNP concentrations in the product cycle as early as possible.

Investigation of recycling rate achievable with sort-out

Sorting out a share of the waste limits the recycling rate achievable. The maximum share of waste allowed to contain phthalates, i.e. either DEHP or DiNP, for being able to achieve the low or high future recycling rate curves is given in Figure S19. These curves consider recycling losses, i.e. that a higher share of waste has to be separately collected and can therefore not contain phthalates than can be actually recycled. They also consider the specificity of the FTIR device, i.e. more waste results to contain phthalates and is therefore sorted out than actually contains phthalates.

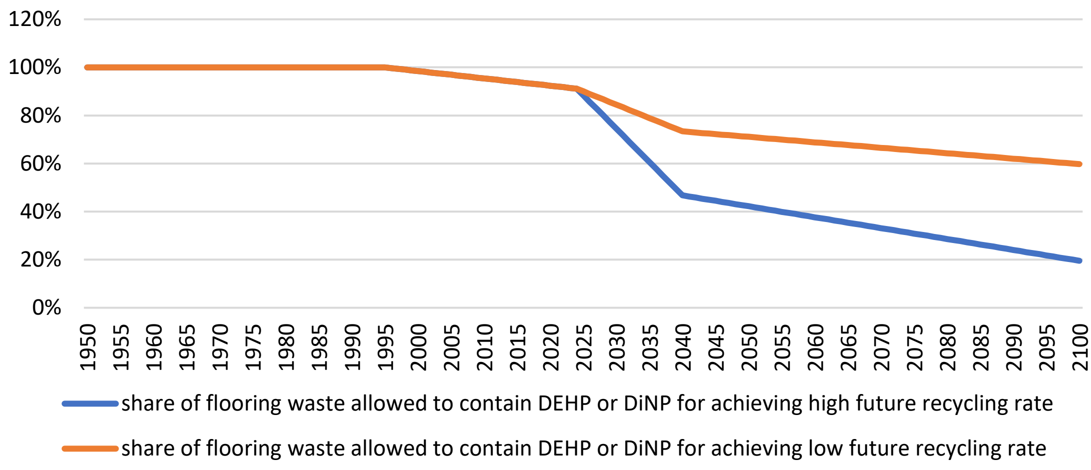

Figure S19: Share of flooring waste over time allowed to contain either DEHP or DiNP for being able to achieve different future recycling rate scenarios

As mentioned, the share of waste that is sorted out depends on the share of waste containing phthalates, the specificity of the measurement device (80.9%[68]; more waste results as positive, i.e. as containing phthalates, than actually contains phthalates) and recycling losses (13%[56]). Which share of waste contains phthalates depends on the phthalates market share in past consumption and the recycling patterns over time. The concentration of phthalates in waste overall will decrease in future based on the model (Figure S12). If the phthalate concentration in waste was not altered by recycling (average phthalate concentration of 26% in phthalate-containing waste as in new flooring), i.e. the waste share containing phthalates only depended on the market shares of phthalates in consumption, the waste share not containing phthalates would be sufficient to achieve the high recycling rate curve (Figure S20). This is as the overall phthalate market share decreases, which matches the increasing recycling rates. If, however, recycling distributes phthalates to a larger number of samples, so that the average phthalate concentration in phthalate-containing waste would only amount to 13%, the situation for achieving high future recycling rates is already tight. Sorting out contaminated waste already from 2025 onwards enables a higher phthalate-free waste share, which makes it easier to achieve high recycling rates (Figure S20 B)).

As a consequence, sort-out shall start as early as possible to avoid distribution of phthalates to large share of floorings, as recycling is likely to dilute phthalates due to blending of recycled and virgin material, which is common. This leads to a higher share of waste containing phthalates and, therefore, needing to be sorted out. Besides, it is anyways desirable to start sort-out as soon as possible to avoid increasing phthalate concentrations in products in the coming years due to higher recycling rates combined with a high share of waste containing phthalates (Figure 5 of the main manuscript).

We did not model situations in which sort-out starts later than in 2025, as our model cannot depict the distribution of phthalates in waste (only their average concentration). Therefore, the model would not be able to depict the effect of a later start of sort-out on the phthalate dilution caused by recycling.

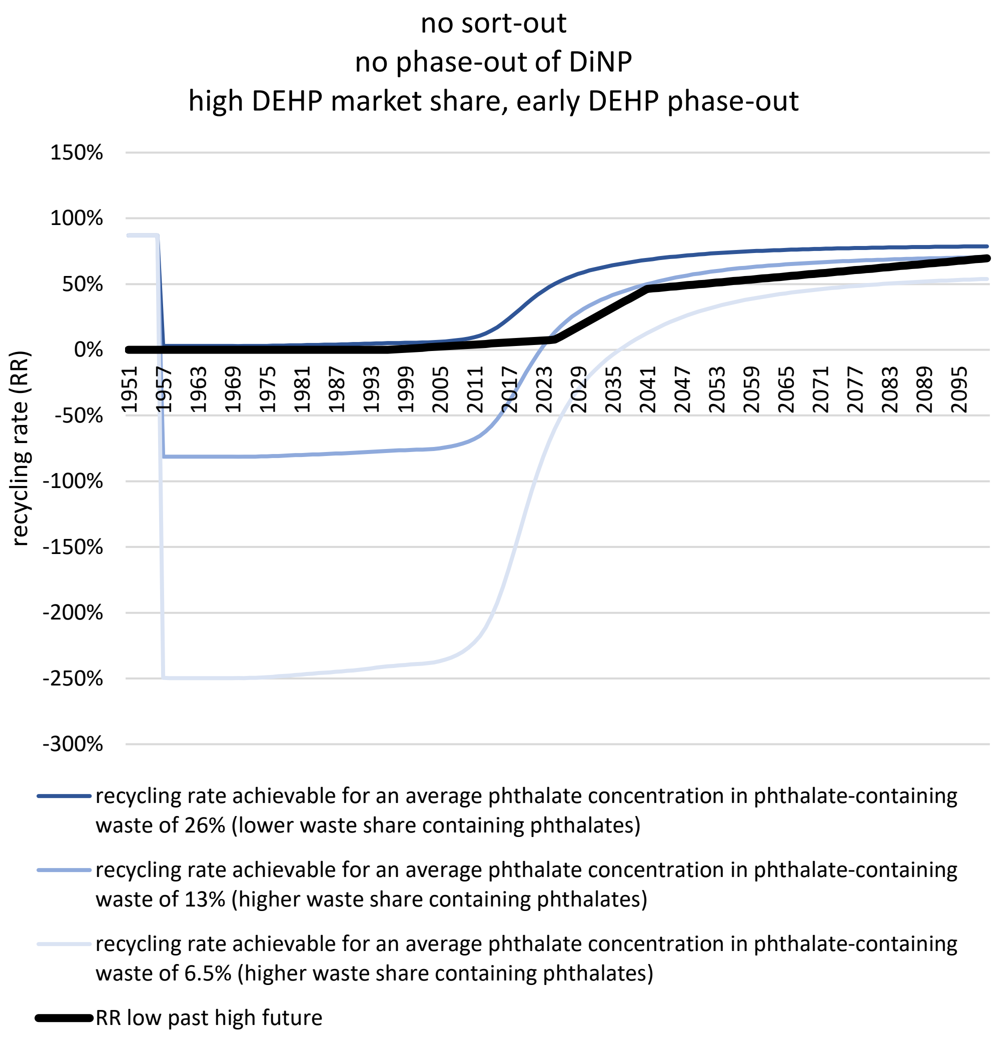
no sort-out
no phase-out of DiNP
high DEHP market share, early DEHP phase-out
150%
100%
50%
0%
-50%
-100%
-150%
-200%
-250%
-300%
recycling rate (RR)
1951
1957
1963
1969
1975
1981
1987
1993
1999
2005
2011
2017
2023
2029
2035
2041
2047
2053
2059
2065
2071
2077
2083
2089
2095
recycling rate achievable for an average phthalate concentration in phthalate-containing waste of 26% (lower waste share containing phthalates)
recycling rate achievable for an average phthalate concentration in phthalate-containing waste of 13% (higher waste share containing phthalates)
recycling rate achievable for an average phthalate concentration in phthalate-containing waste of 6.5% (higher waste share containing phthalates)
RR low past high future

A)

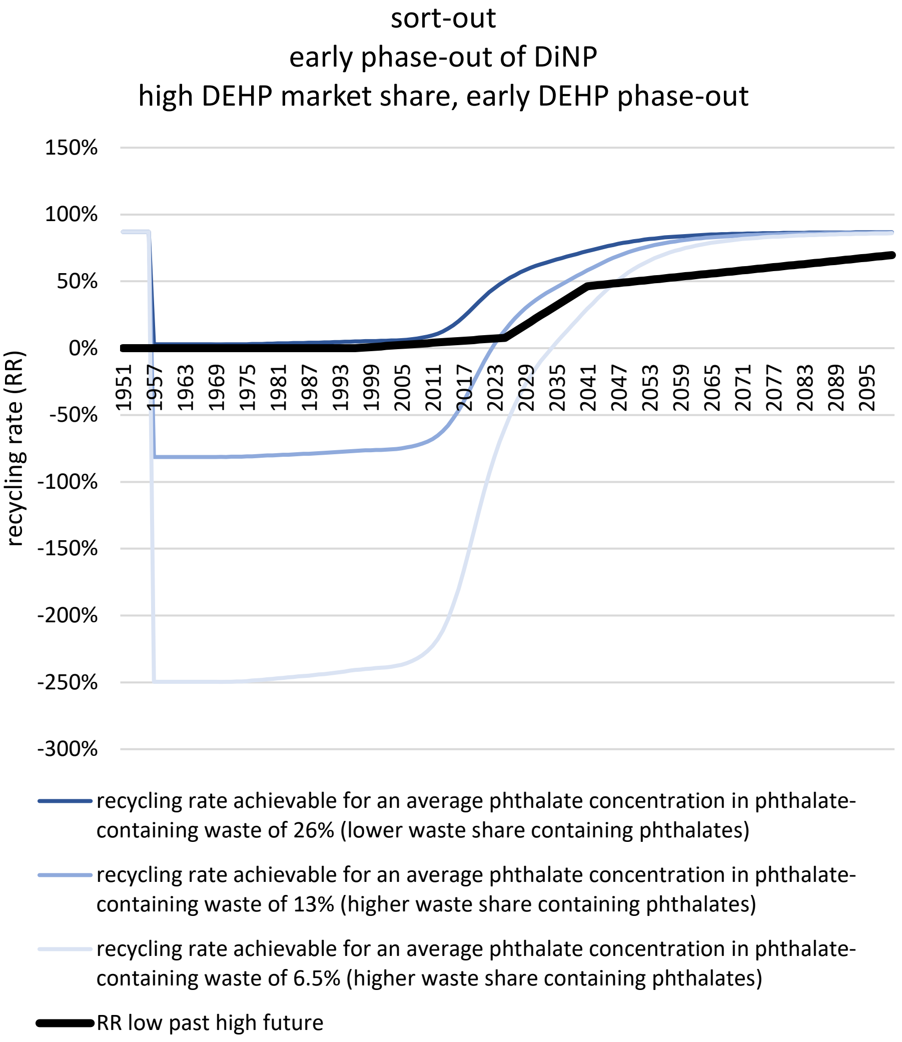

B)

Figure S20: Recycling rates achievable based on the overall phthalate concentration in waste over time from the model, for different phthalate distributions (shares of waste containing phthalates). The share of waste containing phthalates, in one case, is given for a concentration of phthalates in phthalate-containing waste of 26%, which corresponds to the phthalate concentration in new floorings. For a second case, a concentration of phthalates in phthalate-containing waste of only 13%, i.e. half the value for new floorings, is considered, for investigating the effect of a potential dilution caused by recycling. A concentration of only 6.5% was considered as well. The achievable recycling rates were calculated considering that more waste than actually containing phthalates is sorted out (due to measurement specificity) and a higher waste amount needs to be phthalate-free for being collected than is actually recycled due to recycling losses. An average phthalate concentration of 13% or 6.5% in phthalate-containing waste is physically impossible for the past due to high overall phthalate concentrations in waste and results in physically impossible negative recycling rate values. The recycling rates achievable are given for A) the scenario with no sort-out and no phase-out of DiNP and B) a scenario with sort-out and early phase-out of DiNP. Both cases consider high future recycling rates, as well as a high market share of DEHP and early DEHP phase-out.

Selective phthalate removal

Another option for removing phthalates from recycling streams that we considered is solvent-based recycling. This recycling method might be able to remove more than 99% of the phthalates in PVC waste[62,85,86]. We considered 1% of the originally contained phthalates remaining in PVC for solvent-based recycling, i.e. a recycling transfer coefficient (TC) of 1% (from recycling input to secondary material).

Due to the removed phthalates, a considerable share of collected waste may not be recycled in the coming years (as only the PVC is recycled). For achieving a certain recycling rate, therefore, higher collection rates are needed to make up for the phthalate amounts removed in the course of recycling (Figure S21). The required collection rates seem, however, achievable[22,59]. Besides, solutions for transforming removed phthalates into non-hazardous chemicals, which can be recovered, have been developed[62].

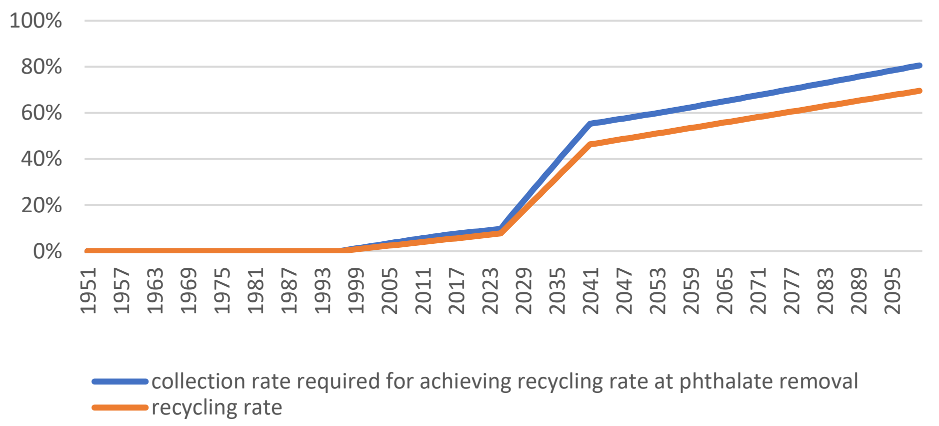

Figure S21: Collection rate required for achieving high future recycling in case of selective phthalate removal from waste (reducing recycled amounts), for the case of a high market share and early phase-out of DEHP, no phase-out of DiNP, solvent-based DEHP and DiNP removal starting in 2025, and low past recycling. The required collection rates are based on the phthalate shares in waste over time and consider recycling losses.

Solvent-based recycling plants will likely not be operational on full industrial scale in the coming years. Therefore, assuming high phthalate removal rates by means of solvent-based recycling in the 2020s is quite optimistic. For the sake of convenience, in our solvent-based recycling scenarios, phthalate removal starts already from 2025 onwards, same as for sort-out of contaminated waste via FTIR. Readers can, however, simply consider the related results for years lying further in the future, as solvent-based recycling is able to immediately reduce phthalate concentrations to very low levels and separate phthalates from PVC. This means that any phthalate distribution to a larger waste share potentially taking place in the coming years would not considerably affect concentrations in the years further ahead.

S1.4.5 Scenario overview

Scenarios DEHP

	**lifetime mean and distribution**	*past* **DEHP concentration**	**DEHP phase-out**	**DiNP concentration**	**DiNP phase-out**	**DEHT concentration**	**RR past**	**plausible? (Section 2.3.1)**	*future* **RR future**	**DEHP removal**
best estimate	*20y lognorm*	high	early				low	x	very low	none
	20y lognorm	high	early				low	x	low	none
	20y lognorm	high	early				low	x	high	none
different lifetimes	*15y lognorm*	high	early				low	x	low	none
	30y lognorm	high	early				low	x	low	none
	20y weibull	high	early				low	x	low	none
different phase-out timeline	*20y lognorm*	high	very early				low	x	high	none
	20y lognorm	high	late				low		high	none
	20y lognorm	high	non-compliance				low		high	none
different market shares	*20y lognorm*	intermediate	early				low	x	high	none
	20y lognorm	low	early				low	x	very low	none
	20y lognorm	low	early				low	x	low	none
	20y lognorm	low	early				low	x	high	none
different recycling rate past	*20y lognorm*	high	early				very low	x	very low	none
	20y lognorm	high	early				very low	x	low	none
	20y lognorm	high	early				very low	x	high	none
	20y lognorm	high	early				high		high	none
plausibility check (very) high recycling rate past	*20y lognorm*	low	early				high		high	none
	20y lognorm	high	early				very high		high	none
plausibility check non-compliance	*20y lognorm*	low	non-compliance				low		high	none
implausibly high	*20y lognorm*	high	non-compliance				very high		high	none
implausibly low	*20y lognorm*	very low*	very early				very low		high	none
plausible parameter combination for future scenario assessment	*20y lognorm*	low	late				low	x	very low	none
	20y lognorm	low	late				low	x	low	none
	20y lognorm	low	late				low	x	high	none
other configurations leading to plausible concentrations	*20y lognorm*	intermediate	early				low	x	low	none
	20y lognorm	intermediate	late				low	x	low	none
contaminant removal at recycling	*20y lognorm*	high	late				low	x	high	solvent-based
	20y lognorm	high	late				low	x	high	sort-out best
	20y lognorm	high	late				low	x	high	sort-out medium

*25%

Scenarios DiNP

	lifetime mean and distribution	*past*						*future*	
		**DEHP concentration**	**DEHP phaseout**	**DiNP concentration**	**DiNP phase-out**	**DEHT concentration**	**RR past**	**RR future**	**DEHP removal**
best estimate	*20y lognorm*			low	none		low	very low	none
	20y lognorm			low	none		low	low	none
	20y lognorm			low	none		low	high	none
different market shares	*20y lognorm*			intermediate	none		low	high	none
	20y lognorm			high	none		low	high	none
different recycling rate past	*20y lognorm*			low	none		high	high	none
	20y lognorm			low	none		very high	high	none
different phase-out timelines	*20y lognorm*			low	late		low	high	none
	20y lognorm			low	early		low	high	none
contaminant removal at recycling	*20y lognorm*			low	none		low	high	solvent-based
	20y lognorm			low	none		low	high	sort-out best
	20y lognorm			low	none		low	high	sort-out medium
	20y lognorm			low	early		low	high	solvent-based
	20y lognorm			low	early		low	high	sort-out best
	20y lognorm			low	early		low	high	sort-out medium
plausible -> test low future RR	*20y lognorm*			intermediate	none		low	low	none

Scenarios DEHT

	lifetime mean and distribution	*past*						*future*	
		**DEHP concentration**	**DEHP phaseout**	**DiNP concentration**	**DiNP phase-out**	**DEHT concentration**	**RR past**	**RR future**	**DEHP removal**
best estimate	*20y lognorm*					high	low	low	none
	20y lognorm					high	low	high	none
different market share	*20y lognorm*					intermediate	low	low	none
	20y lognorm					intermediate	low	high	none

S1.5 Validation

The validation of certain material flow model results based on real-world data is partly difficult, as, for instance on waste amounts, statistics are often not collected, and representative data from chemical analyzes are scarce due to the high effort related to such analyzes[68]. We had Swiss concentration data from measurements available for comparison (Section S1.5.1) and could compare our results to consumption, waste and concentrations values from a similar model for Germany (Section S1.5.2).

S1.5.1 Comparison measurement data

The measurement data used for comparison, while being related to a much larger market share than is the case for most other studies[68], are still not completely representative for the Swiss market. To allow fair comparison with the modelled concentrations, which refer to the market average, a weighted average of the measured concentrations was calculated. As many samples analyzed by Wiesinger et al.[68] stemmed from a professional seller distributing flooring that contained especially high shares of secondary materials[22], these samples were weighted with the market share of that seller (10%[22]). The remaining samples were assumed to be representative for the rest of the market, backed by the fact that several of the remaining companies on the Swiss market sell flooring via DIY stores[22], which coincided with how the rest of the samples were taken.

Measurement weighting may have also been done based on flooring types, which were specified by Wiesinger et al.[68]. However, little information was available regarding specifically high or low use of recycled material in certain flooring types, which is why we omitted such a weighting.

Regarding analysis of the sample data, when calculating mean concentration values, for samples with concentrations below the LOD, we considered concentrations of LOD/2 (which for DEHP amounts to 0.000032/2 weight% and for DiNP to 0.000504/2 weight%[68]). As the LODs are low compared to the resulting average values, the latter are not considerably affected when calculating the average assuming extreme concentrations of LOD or zero in samples below LOD instead. For DEHP, for instance, three significant digits of the percentage value are not affected (unweighted average of 0.324 weight% in all cases).

We did not do a distribution fitting for the measurement data, as the distribution of the concentration used as model input refers to the distribution of the market average concentration and is, thus, a different distribution than the distribution of the sample data.

Due to the weighting involved, the market average concentration based on measurements, used as a reference against which model results were compared, involves uncertainties as well. Therefore, we also considered scenarios leading to mean concentrations to some extent deviating from the weighted measurement average as plausible. Specifically, a deviation of plus/minus two-third of the weighted measurement average was allowed. The deviation allowed is less than 100% of the weighted average measured concentration, as else average DEHP concentrations close to zero resulted as plausible, which, however, seem unlikely, as in many market samples DEHP was found in considerable concentrations[68]. With this procedure, we still consider scenarios with a rather high deviation from the weighted average measured concentration as possible, due to the uncertainties related to the derivation of the market average concentration from the measurements.

Only for the model parameter configurations identified as plausible, we consider future scenarios. This selection of only plausible model configurations for future scenario assessment can be regarded as calibration of the model.

We also compared the market average concentration of phthalates in flooring, which we estimated as model input (Section S1.4.2), with the measured concentrations. For this, we only regarded samples with concentrations considerably above 2 weight%, which is the minimum concentration in which, based on our estimate, phthalates are used as plasticizers in flooring (Section S1.4.2). We assume that lower concentrations measured are due to

recycled content[22]. The concentrations from Wiesinger et al. may have been affected to a minor extent by the fact that the PVC amount was estimated via the chlorine content, which may, for instance, also be related to TiCl.

For DEHT, no quantitative comparison was conducted, as only semi-quantitative data were available from the previous measurements[68].

S1.5.2 Comparison Germany

Description of German model

In the following, the dynamic stock and flow model on DEHP in PVC flooring in Germany is described with regard to model structure and data sources. The model distinguishes between four different types of PVC flooring: compact flooring, cushion vinyl, luxury vinyl tiles, and vinyl-asbestos-flooring. The input of PVC flooring to the building and infrastructure sector is the driver of this model. PVC flooring is consumed and remains in the in-use stock over its lifetime. We model the lifetime of PVC flooring using lognormal and Weibull distributions (cf. Section S1.3.2). At the end of its lifespan, end-of-life (EOL) PVC window flooring undergoes collection and subsequent waste treatment, which includes recycling, incineration, and landfilling. Recycling products are utilized in closed loops to produce new PVC flooring products.

Data on the consumption of PVC flooring in Germany were sourced from the European Resilient Flooring Manufacturers' Institute (ERFMI), covering the period from 1960 to 2022[6]. To forecast future consumption of PVC flooring, we assumed an annual growth rate ranging from -1% to +1%. The differentiation between the four types of PVC flooring for the years 2002 to 2022 was provided in the ERFMI data. For the years preceding 2002, the share of vinyl-asbestos-flooring was calculated based on historical data on asbestos use. A share of 8% of the asbestos consumed in Germany was utilized in PVC flooring until 1993[87] with an asbestos concentration of 60% in vinyl-asbestos flooring (based on LUBW, 2017[88]). The shares of other PVC flooring types were calculated assuming a replacement of vinyl-asbestos flooring with asbestos-free cushion vinyl and a consistent distribution of the other flooring types until 2002. For the years beyond 2022, the distribution of PVC flooring across the four PVC flooring types is assumed to remain constant.

The amount of virgin PVC used in PVC flooring is calculated based on the mass balance of PVC flooring consumption and trade. Data on the trade of PVC flooring by Germany with the rest of the world have been gathered from the UN Comtrade Database for the years 2011 – 2022 (HS Code: 3918.10 "Floor Coverings And Wall Or Ceiling Coverings Of Vinyl Chloride Polymers"; cf. UN, 2022[89]). Within this period, the annual trade volumes of the four flooring types were determined by applying a consistent ratio between trade and consumption for all flooring types. For the period spanning from 1960 to 2010, trade volumes were extrapolated based on the ratio from 2011. Similarly, for the period from 2023 to 2100, the trade ratio calculated for the year 2022 was applied consistently.

After usage, EOL PVC flooring is collected and directed to waste management. Until 1990, all EOL PVC flooring was directed to landfilling. Subsequently, from 1991 to 2005, it is assumed that 50% of the material not destined for recycling is sent to landfilling, based on average data for mixed construction waste[90]. This practice changed in 2006, due to the ban of direct landfilling of such waste[91], resulting in a 0% landfilling rate for this waste stream thereafter.

The share of EOL PVC flooring collected for mechanical recycling is set to 0% until 1993. Since 1994, 2,500 Mg per year of EOL PVC flooring have been directed to a recycling plant in Germany[92]. Vinyl-asbestos-flooring is not accepted by the recycling plant because it is not eligible for mechanical recycling due to its contamination with asbestos fibers. For other PVC flooring types, a recycling yield of 87% for utilization in PVC flooring production is assumed[93]. Until 2005, rejects of the recycling process were directed to landfilling, while from 2006 onwards recycling rejects were incinerated, aligning with landfilling regulations[91]. Any EOL PVC flooring not directed to recycling or landfilling is assumed to be treated by municipal solid waste incineration.

The plasticizer content in new PVC flooring is assumed to remain constant over time within each of the four PVC flooring types. For each of the PVC flooring types, the plasticizer content was determined based on environmental product declarations. According to available references, the average plasticizer content is 19.5% in cushion vinyl and vinyl-asbestos flooring[5], 9.0% in luxury vinyl tiles[4], 13.6% in compact flooring (thereof 80% heterogenous compact flooring and 20% homogenous compact flooring with a plasticizers content of 8.8% in heterogeneous compact flooring[3] and 14.8% in homogeneous compact flooring[2]). The share of DEHP among the plasticizers and scenarios for the phase-out of DEHP are modelled in line with the model for Switzerland based on data for the European plasticizer market (cf. Section 2.3.2 of the main manuscript). Losses of DEHP during use and recycling are modelled following the approach used in the PVC flooring model for Switzerland. The concentration of DEHP in remaining PVC flooring flows is accounted through mass balances.

The rationales for assuming the same values for certain parameters in both the Swiss and the German models were the following. The lifetime distribution of PVC flooring was assumed to be similar due to assumingly similar product portfolios because of similar socio-economic situations[94]. The shares of DEHP among all plasticizers in the two countries, as well, were assumed to not differ considerably from each other due to close connection and similarity of the Swiss and European markets[95]. Regarding the market situation, applying legislations are often aligned between Switzerland and Germany, for instance on substance restriction[63,64]. Use and recycling losses were assumed to be the same due to identical or similar material characteristics and recycling processes. For instance, recycling of Swiss and German flooring wastes both take place in the same plant in Troisdorf (Germany)[24]. The same future recycling rate curves were chosen for comparability, as similar trends can be observed in both countries.

The dynamic substance flow model is implemented in ODYM[7]. A data quality assessment is conducted to characterize the uncertainty associated with model input parameters, employing an approach outlined by Laner et al.[96] for characterizing data uncertainty in material flow analysis. The data quality assessment serves as a basis to assign uncertainty distributions to the input values, assuming a log-normal distribution for their variability. These uncertainty distributions are further utilized in Monte Carlo simulations with 1,000 runs to quantify the uncertainty of model outputs.

S2 Further results and discussion

S2.1 Further MFA uncertainty discussion

Changes in the Swiss PVC flooring production landscape in the time period between 1990 and 2000 made an accurate estimate for individual years difficult, as outlined in the main paper. More specifically, the historical share of all PVC processed in Switzerland used for flooring production is particularly uncertain. This share likely changed from year to year in the period between 1990 and 2000 due to the industry changes taking place. While we have considered the capacities of the plants that closed down and potential plant operation at higher capacity before closure, as well as cross-checked the Swiss production with the gross export amounts, aspects such as short-term material storage were difficult to depict. Therefore, the strong consumption decrease and subsequent increase in the model may have been less distinct in reality, while the overall magnitude of the estimated consumption during that time period should be accurate. Meanwhile, the relevance of the consumption amounts before the year 2000 for future waste flows is lower compared to the consumption in more recent years.

The future consumption amounts are most uncertain due to both population and consumption per capita developments potentially having an either increasing or decreasing trend in the long run.

The utilization of a distribution for consumption modelling, in contrast to scenarios, leads to high fluctuations of consumption from year to year in individual simulation runs. This has the consequence that fluctuations in positive and negative direction compensate each other when waste amounts are calculated (as the total waste arising in a year stems from different consumption years), leading to a comparatively lower uncertainty of waste amounts. Considering scenarios of high, intermediate or low consumption over the whole time period, instead, would have resulted in a larger uncertainty of waste amounts, better representing the real situation (in which consumption is likely to fluctuate either on high, intermediate, or low level, but not from very high values in one year to very low ones in the next). However, the uncertainty distribution approach used allowed us to consider the same underlying material flows for all substance flow analysis (SFA) scenarios. If one was to consider different MFA scenarios as a basis for the SFA scenarios, one would have to model each SFA scenarios with a high, an intermediate and a low MFA future consumption scenario, respectively, and summarize the range of resulting SFA concentrations.

Different lifetime distributions lead to a maximum deviation of the resulting mean waste amounts from the lognormal distribution with a mean of 20 years, used for the best-estimate scenarios, in 2020 by 31%. A possibility that was not assessed was that the lifetime follows a bi-modal distribution, with frequent flooring changes in shops and long lifetimes for schools or hospitals[22,42]. However, as waste amounts from different years even out, we do not assume that a related modelling approach would result in considerably different waste amounts. While the flooring lifetimes may have changed over time, we have not found related evidence and thus assumed that no systematic change occurred.

S2.2 Validation

S2.2.1 Measurement comparison DEHP

For the parameter plausibility check, we also considered further scenarios besides the ones described in the main paper (Section S1.4.5). Even if the DEHP market share had been low in the past, a high recycling rate would have resulted in too high concentrations in comparison with the measurements (0.23%; lying at the upper boundary of the concentration range considered as plausible; cf. Figure 2 of the main paper). Similarly, also non-compliance, even for a low DEHP market share in the past, would have resulted in too high concentrations in comparison with the model (0.5%, i.e. still far above the upper boundary of the concentration range considered as plausible; cf. Figure 2 of the main paper).

A late phase out seems possible to have happened: while the respective scenario with a high DEHP market share, even for a low past recycling rate ends up above the plausible range (0.25%), for a low DEHP market share, plausible concentrations result (0.14%). Still, the model results suggest that phase-out rather occurred early.

We also tested the model by assuming implausibly high (high market share, non-compliance, very high past recycling rate) and low (very low market share, very early phase-out, very low past recycling rate) parameter values (Section S1.4.5). These system configurations resulted in average concentrations in 2021/2022 of 1.41% and 0.02%, respectively, meaning that the model does give concentrations far away from the plausible range for extremer values.

One of the model configurations (low DEHP market share, late phase-out, low past recycling rate) that was considered for future scenario assessment because it results in plausible concentrations (Figure 4 of the main paper), is not shown in Figure 2 of the main paper. This scenario leads to an average DEHP concentration of 0.14 weight% in 2021/2022.

The unweighted average measurement concentration of DEHP amounts to 0.32 weight%, i.e. is more than twice as high as the weighted average concentration.

S2.2.2 Measurement comparison DiNP

For DiNP, model concentrations in 2021/2022 almost three times as high as the weighted measurement average result, independent of the past DEHP market share (Table S2). This is still comparatively close, considering that due to the weighting required also the measurement values are attached to uncertainties. However, it indicates that possibly either DiNP substituted only for a lower proportion of phased-out DEHP than one third as assumed in the model or/and DiNP constituted a lower share of all non-DEHP *ortho*-phthalates in the past than the about one third assumed. It may also be that the plasticizer concentration in floorings has been slightly overestimated (cf. Section S1.4.2).

Table S2: DiNP measured vs modelled concentrations in 2021/2022. RR: recycling rate.

**Value origin**	**DiNP concentration**
Measurements	
Unweighted measurement average	2.13 weight%
Weighted measurement average	1.58 weight%

Model	
High DEHP market share (early phase-out, low past RR)	4.20 weight%
Intermediate DEHP market share (early phase-out, low past RR)	4.42 weight%
Low DEHP market share (early phase-out, low past RR)	4.65 weight%

S2.3 Insights into recycling patterns

While recycled amounts of DEHP until the year 2030 mainly stem from waste with high DEHP concentrations as used until 2015, in later years, DEHP increasingly originates from waste with lower concentrations, stemming from years after 2015 (Figure S22). Recycled DEHP from this waste still results in considerable amounts, as respective waste streams are recycled in large amounts at increasing recycling rates.

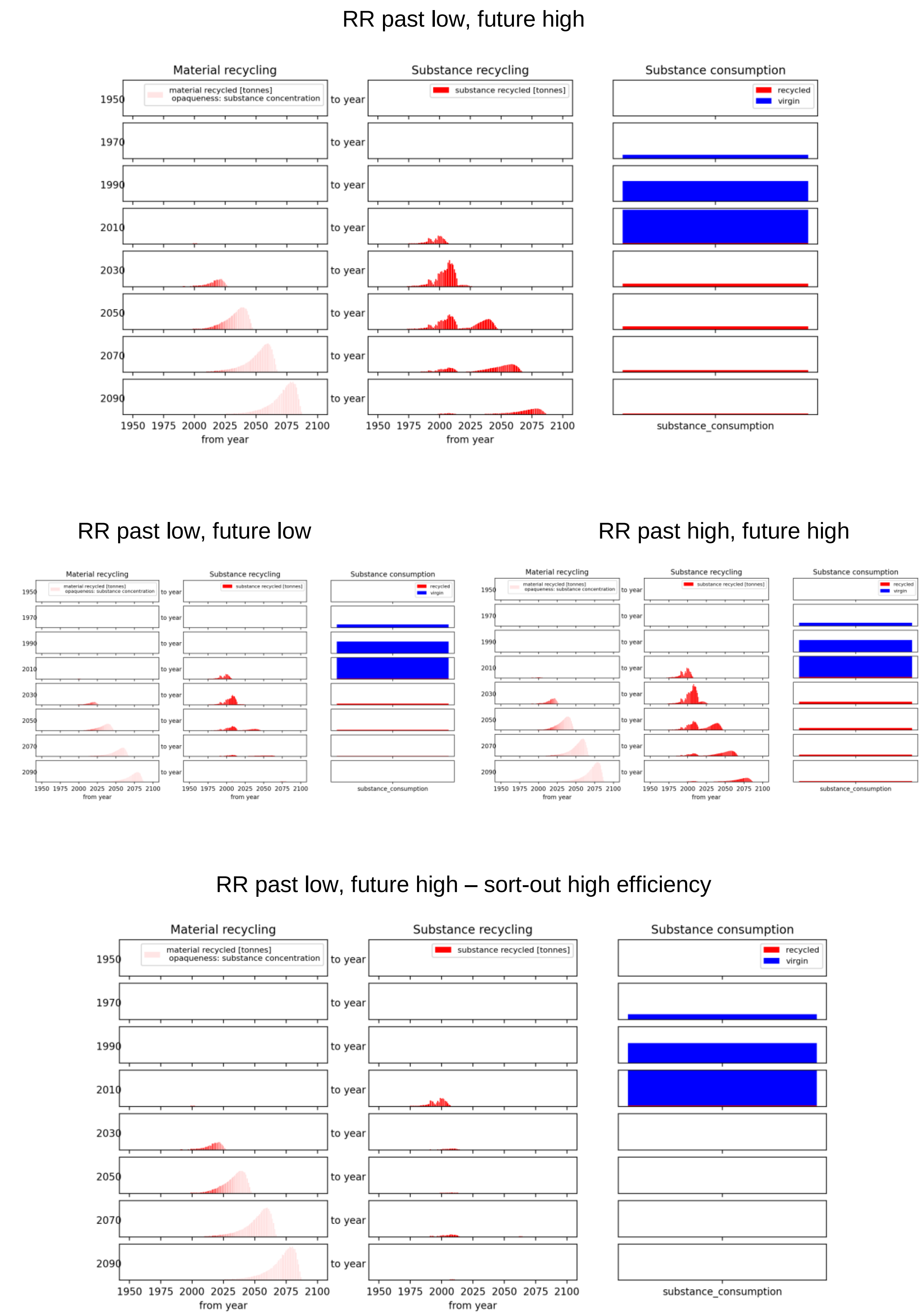

Figure S22: Recycled material and substance amounts of different age cohorts over time. Recycled substance amounts (middle subplot) result from recycled material amounts multiplied with their concentration (minus recycling substance losses or removal). Recycled substance consumption in a year (furthest right subplot) is the sum of the recycled substance amounts of all age cohorts in that year. RR: recycling rate.

The average DEHP concentration in Swiss PVC flooring waste (cf. furthest left subplot in Figure S22), for a high DEHP market share, early phase-out, low past and high future RR and no DEHP removal at recycling, decreases from a value of 20% – as for consumption in the past – down to the values below 1% after around 2045 (Figure S22). In a German project, flooring waste contained an average concentration of 14 weight% of phthalates around 2020[62], which is similar to the modelled situation, reaching this value in 2017.

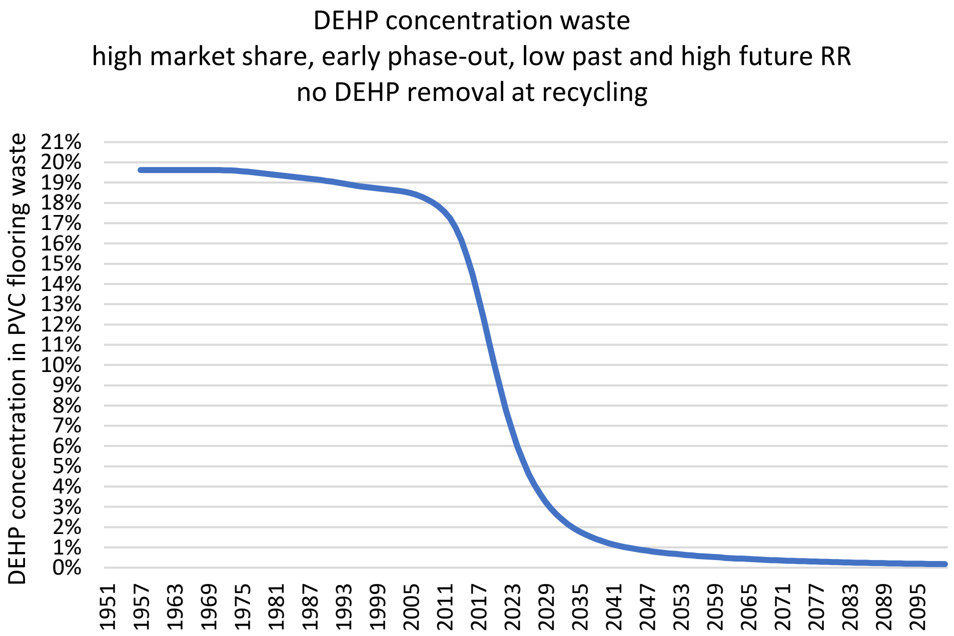

Figure S23: DEHP concentration in waste in a scenario of high past DEHP market shares, early phase-out, a low past and high future recycling rate and no DEHP removal at recycling. RR: recycling rate.

The recycled content resulting from the recycling rate curves considered increases along with the recycling rate (cf. Figure S24 for low past and high future recycling rates).

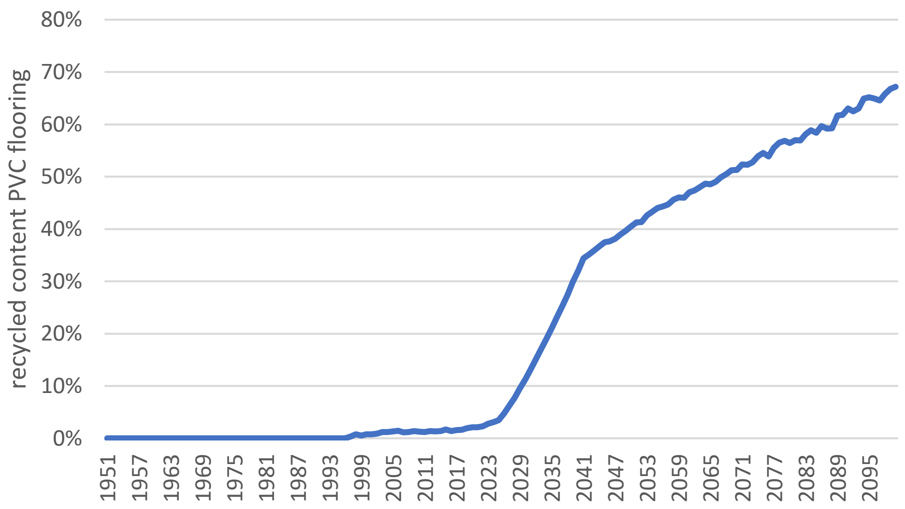

Figure S24: Recycled content in PVC flooring in a scenario of high past DEHP market shares, early phase-out, a low past and high future recycling rate and no DEHP removal at recycling.

An aspect that is not depicted in the model is the distribution of DEHP among different flooring types. For instance, patterns such as a specifically high use of secondary material in luxury vinyl tiles[22,97], which originally contain lower plasticizer amounts than other flooring types[2–5], is not shown. Luxury vinyl tiles are specifically suitable for taking up secondary material, used below the visible cover layer they contain.

If the use of recycled material could be restricted to as few floorings as possible, i.e. use of high secondary material contents are achieved, this would allow to limit the spread of phthalates to a large number of floorings. With this, it could be avoided that many floorings need to be sorted out in future for removing phthalates (Section S1.4.4). Even in case that a sort-out of contaminated waste at recycling is implemented, it would be beneficial to limit the use of secondary PVC to confined products due to non-removable phthalate shares in secondary material (Section S1.4.4).

S2.4 Further results for other substances

S2.4.1 DiNP

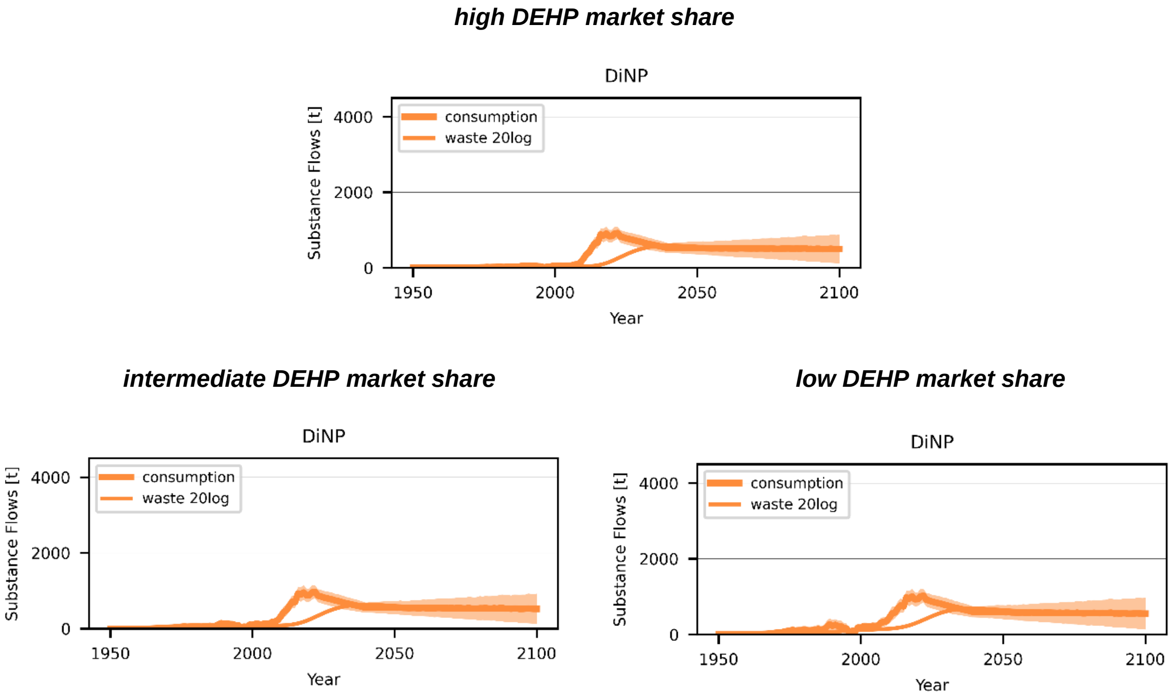

Figure S25: DiNP substance flows for different DEHP market shares, for low past and high future recycling rates, no phase-out as well as an early phase-out of DEHP.

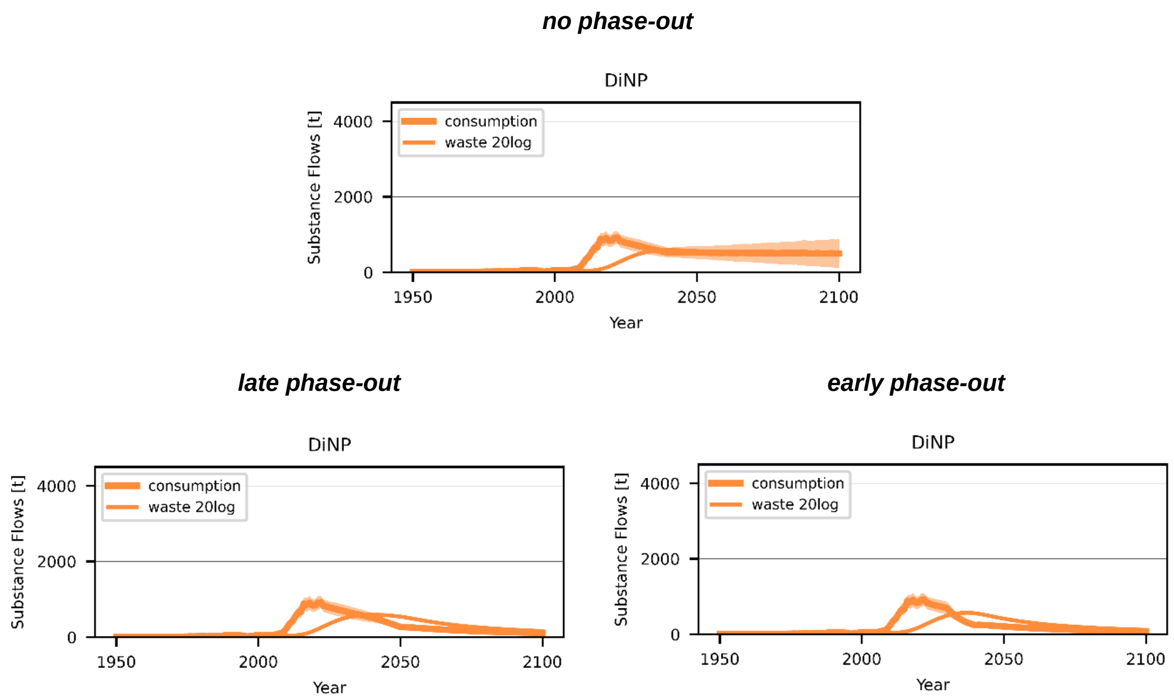

Figure S26: DiNP substance flows for different phase-out scenarios, for low past and high future recycling rates, as well as a high market share and early phase-out of DEHP.

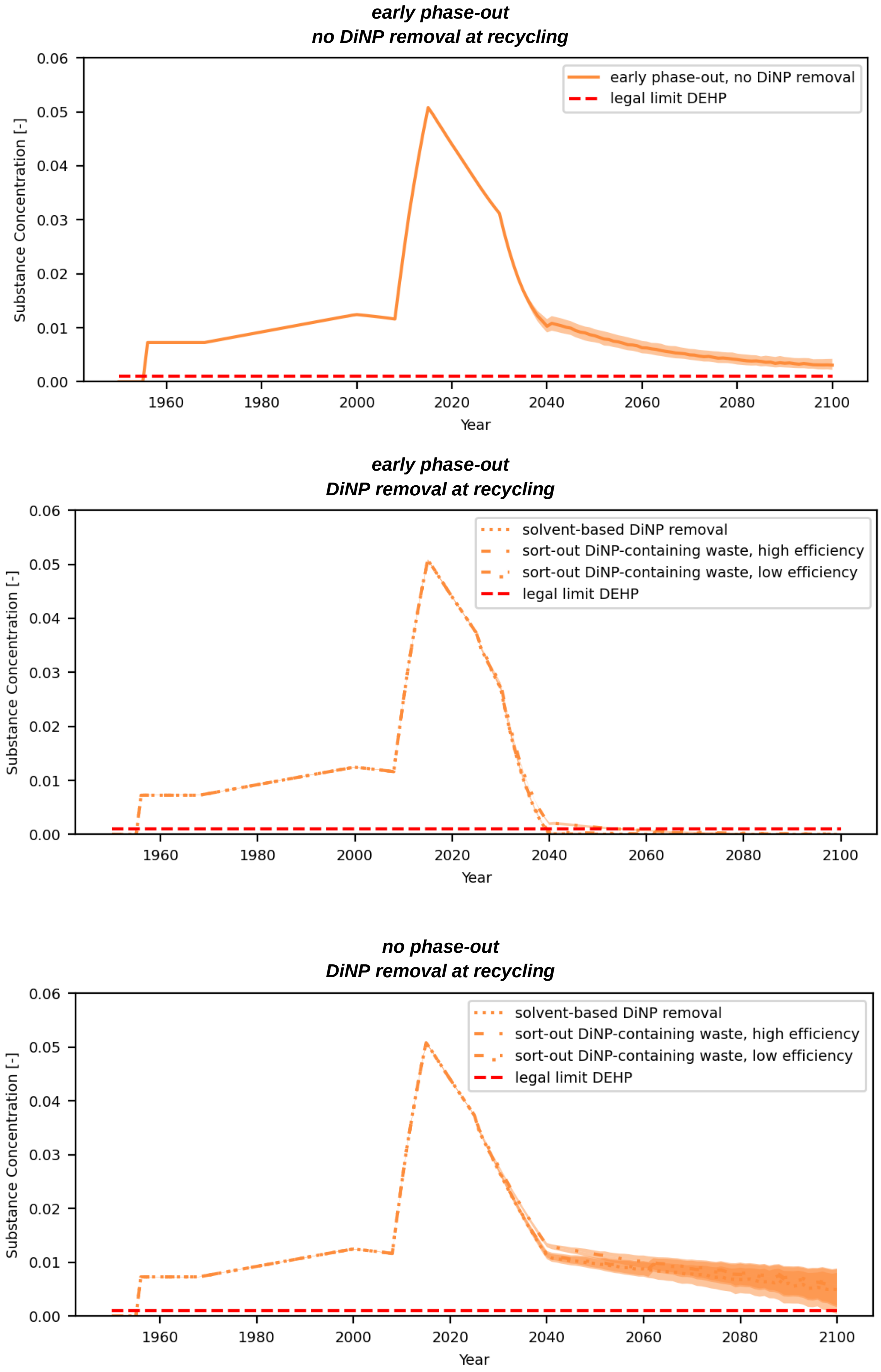

Figure S27: DiNP concentrations for different phase-out and sort-out scenarios, for low past and high future recycling rates, as well as a high market share and early phase-out of DEHP.

S2.4.2 DEHT

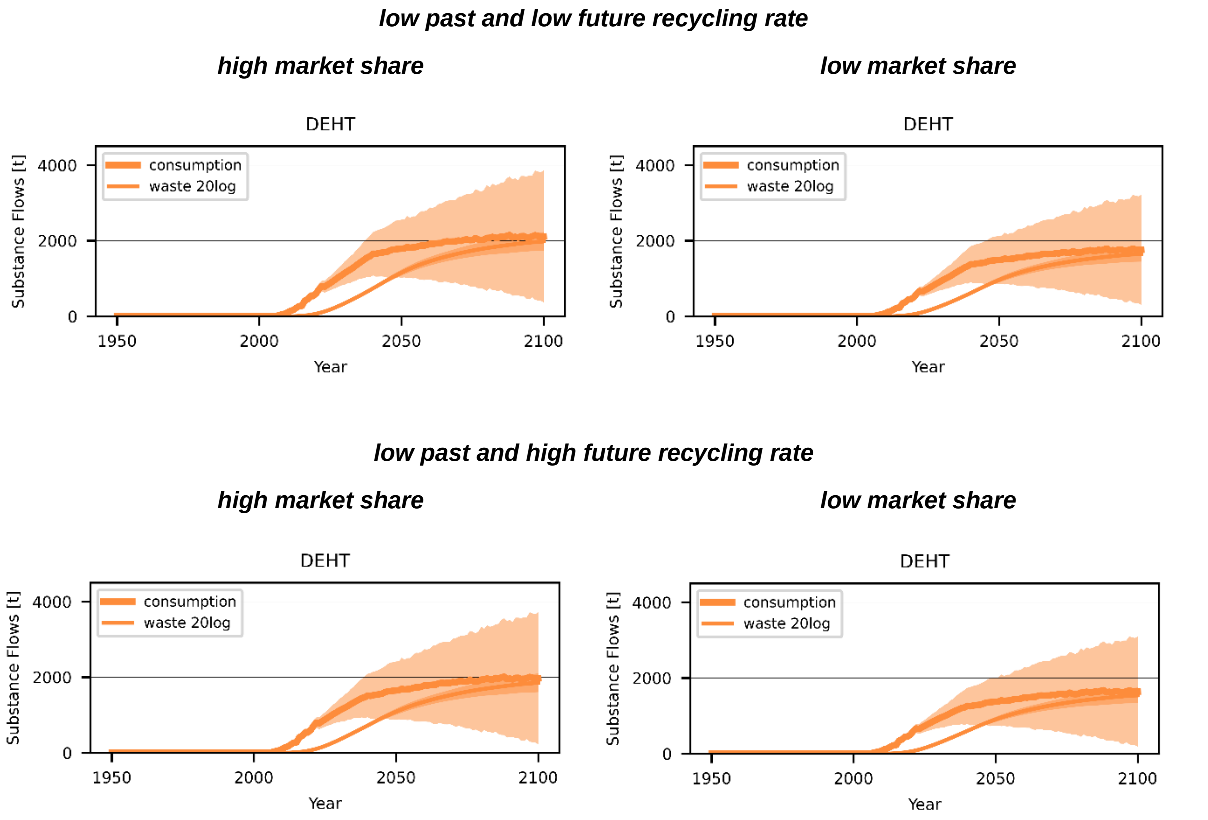

Figure S28: DEHT substance flows for different market shares and recycling rates.The upper left plot corresponds to the DEHT subplot in Figure 1 of the main paper.

S3 References

(1) Branchenradar. *Elastische Bodenbeläge & Laminat in der Schweiz 2023*. https://www.branchenradar.com/de/marktstudien/bodenbelaege-und-technik/elastische-bodenbelaege-und-laminat-in-der-schweiz-2023/ (accessed 2021-04-24).

(2) Tarkett. Environmental Product Declaration. IQ Range Homogeneous Vinyl Flooring. 2018.

(3) Tarkett. Environmental Product Declaration. ACCZENT and RUBY Heterogeneous Vinyl Flooring. 2018.

(4) Resilient Floor Covering Institute. Environmental Product Declaration. Vinyl Tile. 2019.

(5) Tarkett. Environmental Product Declaration. ICONIK Heterogeneous Vinyl Flooring (Expanded (Cushioned) Poly(Vinyl Chloride) Floor Covering). 2020.

(6) ERFMI. Sales of PVC Flooring in Germany. Provided by Jochen Zimmermann (AgPR, Arbeitsgemeinschaft PVC-Bodenbelag Recycling) on 23-10-27. **2023**.

(7) Pauliuk, S.; Heeren, N. ODYM—An Open Software Framework for Studying Dynamic Material Systems: Principles, Implementation, and Data Structures. *J. Ind. Ecol.* **2019**, 1–13. https://doi.org/10.1111/jiec.12952.

(8) Knot, M.; Mulder, K. PVC Plastic: A History of Systems Development and Entrenchment. *Technol. Soc.* **2001**, *23* (2), 265–286.

(9) Westermann, A. *Plastik Und Politische Kultur in Westdeutschland*; 2007. https://doi.org/10.33057/chronos.0849.

(10) Kawecki, D.; Wu, Q.; Gonçalves, J. S. V.; Nowack, B. Polymer-Specific Dynamic Probabilistic Material Flow Analysis of Seven Polymers in Europe from 1950 to 2016. *Resour. Conserv. Recycl.* **2021**, *173* (October 2020). https://doi.org/10.1016/j.resconrec.2021.105733.

(11) Kawecki, D.; Scheeder, P. R. W.; Nowack, B. Probabilistic Material Flow Analysis of Seven Commodity Plastics in Europe. *Environ. Sci. Technol.* **2018**, *52* (17), 9874–9888. https://doi.org/10.1021/acs.est.8b01513.

(12) Federal Statistical Office (FSO). Key Population Figures, 1950-2022, BFS-Nummer Je-e-01.01.01. **2023**.

(13) Statista. *Europäische Union & Eurozone: Entwicklung der Einwohnerzahl in EU und Eurozone im Zeitraum 1960 bis 2022*. https://de.statista.com/statistik/daten/studie/14035/umfrage/europaeische-union-bevoelkerung-einwohner/ (accessed 2021-04-24).

(14) PE Europe GmbH; Institut für Kunststoffkunde und Kunststoffprüfung (IKP); Instituttet for Produktudvikling (IPU) of DTU; RANDA GROUP. *Life Cycle Assessment of PVC and of Principal Competing Materials. Commissioned by the European Commission. Final Report*; Leinfelden-Echterdingen, Lyngby, Barcelona, 2004.

(15) Ciacci, L.; Passarini, F.; Vassura, I. The European PVC Cycle: In-Use Stock and Flows. *Resour. Conserv. Recycl.* **2017**, *123*, 108–116. https://doi.org/10.1016/j.resconrec.2016.08.008.

(16) 0.632.11 Internationales Übereinkommen Über Das Harmonisierte System Zur Bezeichnung Und Codierung Der Waren. Abgeschlossen in Brüssel Am 14. Juni

1983. Von Der Bundesversammlung Genehmigt Am 10. Juni 1986. Schweizerische Ratifikationsurkunde Hinterlegt A. **2022**.

(17) Swiss Federal Office for Customs and Border Security (FOCBS). Swiss-Impex. **2024**.

(18) Chemical Week. Product Focus: Polyvinyl Chloride. *Mater. Sci. Eng. Collect.* **1995**, *156* (13), 64.

(19) Chemical Week. Product Focus: Polyvinyl Chloride. *Mater. Sci. Eng. Collect.* **1997**, *159* (48), 31.

(20) Helminiak, N. Previous Managing Director of Arbeitsgemeinschaft Der Schweizerischen PVC-Industrie (PVCH), Aarau, Switzerland. Personal Communication, 24-02-09 and 24-03-08. **2024**.

(21) Bundesamt für Statistik (BFS). *Produktion, Aufträge, Umsatz*. https://www.bfs.admin.ch/bfs/de/home/statistiken/industrie-dienstleistungen/produktion-auftraege-umsatz.html (accessed 2021-04-24).

(22) Fässler, M. CEO of Gerflor FEAG, Stäfa, Switzerland and President of Arbeitsgemeinschaft Recycling PVC-Bodenbeläge (ARP) Schweiz, Oberentfelden, Switzerland. Personal Communication, 23-12-01. **2023**.

(23) Schiller, M. *PVC Additives. Performance, Chemistry, Developments, and Sustainability*, 2nd ed.; Hanser: München, 2022.

(24) Klotz, M.; Haupt, M. A High-Resolution Dataset on the Plastic Material Flows in Switzerland. *Data Br.* **2022**, *41*, 108001. https://doi.org/10.1016/j.dib.2022.108001.

(25) Swiss Federal Statistical Office. Szenarien Zur Bevölkerungsentwicklung Der Kantone Der Schweiz 2020-2050 - Ständige Wohnbevölkerung Nach Kanton Gemäss Dem Referenzszenario AR-00-2020, 2020-2050. FSO Number Je-d-01.03.02.01. 2020.

(26) United Nations Environment Programme (UNEP). *Global Resources Outlook 2024. Summary for Policymakers: Bend the Trend – Pathways to a Liveable Planet as Resource Use Spikes*; International Resource Panel: Nairobi, 2024.

(27) IPCC. *Climate Change 2022 - Mitigation of Climate Change - Full Report*; 2022.

(28) European Chemicals Agency (ECHA). *Investigation Report on PVC and PVC Additives*; Helsinki, 2023.

(29) Julie Raynaud; UNEP. *VALUING Plastic*.

(30) Bakker, V.; Verburg, P. H.; van Vliet, J. Trade-Offs between Prosperity and Urban Land per Capita in Major World Cities. *Geogr. Sustain.* **2021**, *2* (2), 134–138. https://doi.org/10.1016/j.geosus.2021.05.004.

(31) Fortune Business Insights. *Vinyl Flooring Market Size, Share & Industry Analysis, By Type (Vinyl Sheets, Vinyl Tiles, and Luxury Vinyl Tiles), By End-Use (Residential and Non-Residential), and Regional Forecast, 2024-2032*. https://www.fortunebusinessinsights.com/vinyl-flooring-market-103059 (accessed 2006-07-24).

(32) Grand View Research. *Europe Vinyl Flooring Market Size, Share & Trends Analysis Report By Product (LVT, Vinyl Tiles), By Application (Residential, Commercial), By Country (U.K., Germany, Russia), And Segment Forecasts, 2020 - 2027. Report Overview*. https://www.grandviewresearch.com/industry-analysis/europe-vinyl-flooring-market (accessed 2006-07-24).

(33) Statista. *Entwicklung der Gesamtbevölkerung Deutschlands von 1871 bis 2023*.

(34) Statista. *Prognose der Einwohnerzahl von Deutschland von 2022 bis 2070*. https://de.statista.com/statistik/daten/studie/1446/umfrage/bevoelkerungsvorausberechnung-deutschland/ (accessed 2006-07-24).

(35) Growth Market Reports. *Global Polyvinyl Chloride (PVC) Floor Market*. https://growthmarketreports.com/report/polyvinyl-chloride-pvc-floor-market-global-industry-analysis (accessed 2006-07-24).

(36) Wiprächtiger, M.; Rapp, M.; Hellweg, S.; Shinde, R.; Haupt, M. Turning Trash into Treasure: An Approach to the Environmental Assessment of Waste Prevention and Its Application to Clothing and Furniture in Switzerland. *J. Ind. Ecol.* **2022**, *26* (4), 1389–1405. https://doi.org/10.1111/jiec.13275.

(37) Zink, T.; Geyer, R. Circular Economy Rebound. *J. Ind. Ecol.* **2017**, *21* (3), 593–602. https://doi.org/10.1111/jiec.12545.

(38) Kleijn, R.; Huele, R.; van der Voet, E. Dynamic Substance Flow Analysis: The Delaying Mechanism of Stocks, with the Case of PVC in Sweden. *Ecol. Econ.* **2000**, *32* (2), 241.

(39) Glöser, S.; Soulier, M.; Tercero Espinoza, L. A. Dynamic Analysis of Global Copper Flows. Global Stocks, Postconsumer Material Flows, Recycling Indicators, and Uncertainty Evaluation. *Environ. Sci. Technol.* **2013**, *47* (12), 6564–6572. https://doi.org/10.1021/es400069b.

(40) Geyer, R.; Jambeck, J. R.; Law, K. L. Production, Use, and Fate of All Plastics Ever Made. *Sci. Adv.* **2017**, *3* (7), e1700782. https://doi.org/10.1126/sciadv.1700782.

(41) Muchangos, L. dos; Xue, M.; Zhou, L.; Kojima, N.; Machimura, T.; Tokai, A. Flows, Stocks, and Emissions of DEHP Products in Japan. *Sci. Total Environ.* **2019**, *650*, 1007–1018. https://doi.org/10.1016/j.scitotenv.2018.09.077.

(42) Kleijn, R.; Huele, R.; Van Der Voet, E. Dynamic Substance Flow Analysis: The Delaying Mechanism of Stocks, with the Case of PVC in Sweden. *Ecol. Econ.* **2000**, *32* (2), 241–254. https://doi.org/10.1016/S0921-8009(99)00090-7.

(43) ELSHKAKI, A. Dynamic Stock Modelling: A Method for the Identification and Estimation of Future Waste Streams and Emissions Based on Past Production and Product Stock Characteristics*1. *Energy* **2005**, *30* (8), 1353–1363. https://doi.org/10.1016/j.energy.2004.02.019.

(44) Pivnenko, K.; Laner, D.; Astrup, T. F. Material Cycles and Chemicals: Dynamic Material Flow Analysis of Contaminants in Paper Recycling. *Environ. Sci. Technol.* **2016**, *50* (22), 12302–12311. https://doi.org/10.1021/acs.est.6b01791.

(45) Melo, M. T. Statistical Analysis of Metal Scrap Generation: The Case of Aluminium in Germany. *Resour. Conserv. Recycl.* **1999**, *26* (2), 91–113. https://doi.org/10.1016/S0921-3449(98)00077-9.

(46) Lawless, J. F. *Statistical Models and Methods for Lifetime Data*; 1983; Vol. 25. https://doi.org/10.2307/1267739.

(47) Tukker, A.; Kleijn, R.; Van Oers, L.; Smeets, E. Combining SFA and LCA : The Swedish PVC Analysis. *J. Ind. Ecol.* **1997**, *1* (4), 93–116. https://doi.org/10.1162/jiec.1997.1.4.93.

(48) Jonsson, A.; Fridén, U.; Thuresson, K.; Sörme, L. Substance Flow Analyses of Organic Pollutants in Stockholm. *Water, Air, Soil Pollut. Focus* **2008**, *8* (5–6), 433–443. https://doi.org/10.1007/s11267-008-9185-7.

(49) Association of Plastics Manufacturers in Europe (APME). *Plastics. A Material of Choice in Building and Construction. Plastics Consumption and Recovery in Western Europe 1995*; Brussels, 1995.

(50) Schweizerischer Mieterschutz. *Lebensdauertabelle*. https://www.schweizerischer-mieterschutz.ch/mietrecht/lebensdauertabelle.html/53 (accessed 2011-04-24).

(51) Tarkett. Professional Sales – Eingeschränkte Garantie. Frankenthal 2009.

(52) Arbeitsgemeinschaft Recycling PVC-Bodenbeläge (ARP) Schweiz. PVC-Bodenbelagsrecycling. Zum Vorteil von Umwelt, Bauherr Und Unternehmer. 1996. https://arpschwe.myhostpoint.ch/wp-content/uploads/2012/12/PVC-Bodenbelagsrecycling.pdf.

(53) Plinke, E.; Wenk, N.; Wolff, G.; Castiglione, D.; Palmark, M. *Mechanical Recycling of PVC Wastes. Study for DG XI of the European Commission. Final Report*; Prognos, Plastic Consult, COWI: Basel, Milan, Lyngby, 2000.

(54) Vinyl Plus. *Progress Report 2023*; Brussels, 2023. https://www.vinylplus.eu/wp-content/uploads/2023/07/VinylPlus_ProgressReport_Digital_2023.pdf.

(55) Fässler, M. CEO of Gerflor FEAG, Stäfa, Switzerland and President of Arbeitsgemeinschaft Recycling PVC-Bodenbeläge (ARP) Schweiz, Oberentfelden, Switzerland. Personal Communication, 21-06-03. 2021.

(56) Zimmermann, J. Jochen Zimmermann, Managing Director, AG PVC-Bodenbelag Recycling (AgPR), Personal Communication, 23-11-10. **2023**.

(57) Statista. *European Union: total population from 2010 to 2022*. https://www.statista.com/statistics/253372/total-population-of-the-european-union-eu/ (accessed 2021-04-24).

(58) Gerflor. *Second life*. https://www.gerflor.com/environment/second-life.html (accessed 2021-04-24).

(59) Klotz, M.; Haupt, M.; Hellweg, S. Potentials and Limits of Mechanical Plastic Recycling. *J. Ind. Ecol.* **2023**, *27* (4), 1043–1059. https://doi.org/10.1111/jiec.13393.

(60) Van der Haegen, P. Patric Van Der Haegen, Eberhard, Personal Communication, 22-01-25. **2022**.

(61) Klotz, M.; Haupt, M.; Hellweg, S. Limited Utilization Options for Secondary Plastics May Restrict Their Circularity. *Waste Manag.* **2022**, *141*, 251–270. https://doi.org/10.1016/j.wasman.2022.01.002.

(62) Circular Flooring. Circular Flooring. Neue Produkte Aus Gebrauchten PVC Bodenbelägen Und Sichere Behandlung von Altweichmachern. Presentation, 21-10-06. 2021.

(63) The European Parliment and the Council of the European Union. Regulation (EC) No 1907/2006 of the European Parliament and of the Council of 18 December 2006 Concerning the Registration, Evaluation, Authorisation and Restriction of Chemicals (REACH), Establishing a European Chemicals Agency, Amending Directive 1999/4. 2006.

(64) Swiss Federal Council. SR 814.81 Verordnung Zur Reduktion von Risiken Beim Umgang Mit Bestimmten Besonders Gefährlichen Stoffen, Zubereitungen Und Gegenständen (Chemikalien-Risikoreduktions-Verordnung, ChemRRV) Vom 18. Mai 2005 (Stand Am 1. Januar 2024). 2024.

(65) Gärtner, E. Chancen Für Das PVC-Recycling. *SwissPlastics* **2008**, *4*, 16–17.

(66) Helminiak, N. Managing Director of Arbeitsgemeinschaft Recycling PVC-Bodenbeläge (ARP) Schweiz, Oberentfelden, Switzerland. Personal Communication, 19-10-25. **2019**.

(67) Ooms, J.; Cuperus, J. G. Impact of Lead Restrictions on the Recycling of PVC. **2013**, No. July, 9.

(68) Wiesinger, H.; Bleuler, C.; Christen, V.; Favreau, P.; Hellweg, S.; Langer, M.; Pasquettaz, R.; Schönborn, A.; Wang, Z. Legacy and Emerging Plasticizers and Stabilizers in PVC Floorings and Implications for Recycling. *Environ. Sci. Technol.* **2024**, *58* (4), 1894–1907. https://doi.org/10.1021/acs.est.3c04851.

(69) Carroll, W. F.; Johnson, R. W.; Moore, S. S.; Paradis, R. A. Poly(Vinyl Chloride). In *Applied Plastics Engineering Handbook*; Elsevier, 2011; pp 61–76. https://doi.org/10.1016/B978-1-4377-3514-7.10005-4.

(70) Wypych, G. PVC Additives. In *PVC Formulary*; 2020; pp 47–94. https://doi.org/10.1016/b978-1-927885-63-5.50006-9.

(71) European Plasticisers; IHS. *Plasticisers*. https://www.plasticisers.org/plasticisers/ (accessed 2018-03-24).

(72) S&P Global. *Plasticizers. Chemical Economics Handbook*. https://www.spglobal.com/commodityinsights/en/ci/products/plasticizers-chemical-economics-handbook.html (accessed 2018-03-24).

(73) Grossman, R. F. *Handbook of Vinyl Formulating*, 2nd ed.; John Wiley & Sons: Hoboken, 2008.

(74) Uwe Storzum. BASF Plasticizer Presentation. Presentation, March 2010, Pasadena, Texas. 2010. https://www.researchgate.net/publication/280051725_BASF_Plasticizer_Presentation.

(75) SciFinder. *References for Substance with CAS Registry Number 117-81-7, Substance Role: Adverse Effect*. https://scifinder-n.cas.org/search/reference/662bf459026ff92732af0339/1 (accessed 2011-07-24).

(76) European Chemicals Agency (ECHA). *Candidate List of substances of very high concern for Authorisation*. https://echa.europa.eu/candidate-list-table (accessed 2016-04-24).

(77) European Chemicals Agency (ECHA). *Verzeichnis der zulassungspflichtigen Stoffe. Bis(2-ethylhexyl) phthalate (DEHP)*. https://echa.europa.eu/de/authorisation-list/-/dislist/details/0b0236e1807e0026 (accessed 2016-04-24).

(78) European Chemicals Agency (ECHA). *Authorisation List*. https://echa.europa.eu/authorisation-list (accessed 2018-03-24).

(79) Slob, W. Uncertainty Analysis in Multiplicative Models. *Risk Anal.* **1994**, *14* (4), 571–576. https://doi.org/10.1111/j.1539-6924.1994.tb00271.x.

(80) Umwelt-Probenbank des Bundes. *Diethylhexylterephthalat (DEHTP)*. https://www.umweltprobenbank.de/de/documents/profiles/analytes/27747 (accessed 2018-03-24).

(81) Fantke, P.; Huang, L.; Overcash, M.; Griffing, E.; Jolliet, O. Life Cycle Based Alternatives Assessment (LCAA) for Chemical Substitution. *Green Chem.* **2020**, *22* (18), 6008–6024. https://doi.org/10.1039/D0GC01544J.

(82) Bartle, K. D.; Myers, P. History of Gas Chromatography. *TrAC Trends Anal. Chem.* **2002**, *21* (9–10), 547–557. https://doi.org/10.1016/S0165-9936(02)00806-3.

(83) Kuki, Á.; Nagy, L.; Zsuga, M.; Kéki, S. Fast Identification of Phthalic Acid Esters in Poly(Vinyl Chloride) Samples by Direct Analysis in Real Time (DART) Tandem Mass Spectrometry. *Int. J. Mass Spectrom.* **2011**, *303* (2–3), 225–228. https://doi.org/10.1016/j.ijms.2011.02.011.

(84) P S, A.; Vinod, V.; Harathi, P. B. A Critical Review on Extraction and Analytical Methods of Phthalates in Water and Beverages. *J. Chromatogr. A* **2022**, *1675*, 463175. https://doi.org/10.1016/j.chroma.2022.463175.

(85) Strobl, L.; Diefenhardt, T.; Schlummer, M.; Leege, T.; Wagner, S. Recycling Potential for Non-Valorized Plastic Fractions from Electrical and Electronic Waste. *Recycling* **2021**, *6* (2), 33. https://doi.org/10.3390/recycling6020033.

(86) Pelto, J.; Barreto, C.; Anwar, H.; Strobl, L.; Schlummer, M. Compatibilized PC/ABS Blends from Solvent Recycled PC and ABS Polymers from Electronic Equipment Waste. *Polym. Test.* **2023**, *120*, 107969. https://doi.org/10.1016/j.polymertesting.2023.107969.

(87) BAuA. National Asbestos Profile for Germany. Dortmund, Berlin, Dresden: Federal Institute for Occupational Safety and Health. **2014**.

(88) LUBW. Steckbrief „Vinyl-Asbest-Platten (Auch Floor-Flex Oder Flex-Platten)“. **2017**.

(89) UN. *Trade Data*. https://comtradeplus.un.org/TradeFlow (accessed 2024-10-23).

(90) Arbeitsgemeinschaft Kreislaufwirtschaftsträger Bau. Monitoring-Bericht Baubfälle (Monitoring Report on Construction Waste). **2000**.

(91) Bundesrat. Abfallablagerungsverordnung (Waste Disposal Ordinance). **2001**.

(92) Zimmermann. Recyclinginitiativen Der Europäischen Hersteller von PVC-Fußbodenbelägen. Presentation Held at PVC-Plastisole, Würzburg, 2022. *2022*.

(93) Zimmermann, A. Teams Meeting on November 10th, 2023. **2023**.

(94) World Bank. *Indicators*. https://data.worldbank.org/indicator (accessed 2024-04-24).

(95) State Secretariat for Economic Affairs (SECO). *Europäische Union (EU)*. https://www.seco.admin.ch/seco/de/home/Aussenwirtschaftspolitik_Wirtschaftliche_Zusammenarbeit/Wirtschaftsbeziehungen/Wirtschaftsbeziehungen_mit_der_EU.html (accessed 2018-03-24).

(96) Laner, D.; Feketitsch, J.; Rechberger, H.; Fellner, J. A Novel Approach to Characterize Data Uncertainty in Material Flow Analysis and Its Application to Plastics Flows in Austria. *J. Ind. Ecol.* **2016**, *20* (5), 1050–1063. https://doi.org/10.1111/jiec.12326.

(97) Tarkett. *Corporate Social & Environmental Responsibility Report 2023*; Paris La Défense, 2023.
